# Burden of urogenital congenital anomalies: findings from the global burden of disease study 2021

**DOI:** 10.3389/fped.2025.1584280

**Published:** 2025-09-25

**Authors:** Yiwei Yue, Qian Zhang, Ji Li, Lei Wang, Lihua Guo, Quan Sun

**Affiliations:** Department of Pediatric Surgery, The First Affiliated Hospital of Zhengzhou University, Zheng Zhou, China

**Keywords:** annual percentage change, average annual percentage change, frontier analysis, urogenital congenital anomalies, decomposition analysis, cross-country health inequality analysis

## Abstract

**Background:**

Urogenital congenital anomalies (UCAs) are among the most common organ system abnormalities in the neonate. This study employs Global Burden of Disease, Injury, and Risk Factor Study 2021 (GBD 2021) to systematically quantify the global epidemiological burden of UCAs from 1990 to 2021, examining geographic variations and temporal trends.

**Materials and methods:**

Data were extracted from the GBD 2021. We focused on UCAs-related metrics: prevalence, incidence, deaths, and disability-adjusted life years (DALYs) across 204 countries and territories, grouped into 5 or 21 GBD regions by the socio-demographic index (SDI). Data analysis encompassed relative change calculations, as well as annual percentage change (APC) and average annual percentage change (AAPC), both of which are based on joinpoint regression analysis. The study additionally employed decomposition analysis, frontier analysis and cross-country health inequality analysis. Analyses utilized R version 4.3.1.

**Results:**

From 1990 to 2021, the global prevalence of UCAs cases surged by 21%, exceeding 6.34 million, accompanied by an increase in age-standardized rates. Incidence, mortality, and DALYs experienced declines in both absolute figures and age-standardized rates. Gender-specific trends from 1990 to 2021 revealed that females dominated in prevalence, while males had higher mortality and DALYs burden. Regionally, Southern Sub-Saharan Africa exhibited the highest prevalence, while Oceania and East Asia showed significant increases and decreases, respectively. Decomposition analysis of change in prevalence indicated that, globally, population contributed the most to changes in prevalence, followed by aging and epidemiological change. Frontier analysis of 204 countries and regions linked higher SDIs to lower UCAs prevalence. However, unexpected deviations were observed in some high SDI countries, such as Singapore and South Korea. Health inequality analysis revealed that the health inequality in prevalence between high-income and low-income countries decreased in 2021, although the burden became more concentrated in low-income countries.

**Conclusion:**

Our analysis highlights the intricate relationship between socio-demographic factors and UCAs trends, underscoring the urgent need for targeted and context-specific healthcare interventions. While significant advancements have been made, sustained vigilance and ongoing research remain critical to achieving effective global management of UCAs.

## Introduction

Urogenital congenital anomalies (UCAs) are defined as “any live birth with a urinary or genital condition.” These conditions are often detected either prenatally or immediately after birth, though a significant proportion is identified in older children, presenting with varying degrees of severity (1). UCAs frequently manifest as urinary incontinence, recurrent urinary tract infections, atypical genitalia, or other abdominal complications resulting from congenital abnormalities of the urinary system (2). Unfortunately, UCAs are not only a significant cause of postnatal mortality but also contribute to poor long-term health outcomes (3). With the global population increasing and demographic structures shifting, understanding the comprehensive burden of these conditions has become critically important.

The Global Burden of Disease (GBD) study provides a valuable platform for assessing the health impact of a wide range of diseases and injuries (4). By leveraging this resource, researchers and policymakers can gain insights into the prevalence, incidence, mortality, and disability-adjusted life years (DALYs) associated with specific conditions, offering a panoramic view of their global, regional, and national impacts (5–7). Although the impact of urogenital congenital anomalies on global health is well-established, systematic analyses of their comprehensive burden, particularly those utilizing GBD data, remain sparse.

Furthermore, while statistical assessments provide a foundational understanding, complementary analyses such as decomposition, frontier, and health inequality analyses offer deeper insights into the complexities of the data, painting a more nuanced picture of disease impacts. Decomposition analysis disaggregates the changes in key indicators of UCAs from 1990 to 2021 into three explanatory factors: population growth, aging, and epidemiological changes (8). Frontier analysis establishes a benchmark based on countries with the lowest disease burden at each respective SDI level, defining an efficiency frontier. The greater the distance between a country's actual disease burden and this frontier, the larger the potential for improvement in reducing its burden (9). Health inequality analysis assesses both absolute and relative disparities in UCAs burden by calculating the slope index of inequality and the concentration index, respectively (10). These advanced analyses benchmark health performance against the best-performing counterparts, providing insights into potential areas for improvement and driving more effective health responses.

Given these gaps in research and the immense value such studies can bring, our research aims to systematically elucidate the global, regional, and national burdens of urogenital congenital anomalies. Utilizing the GBD dataset, this study offers a comprehensive perspective on the prevalence, incidence, mortality, and DALYs associated with these conditions. We identified and discussed critical trends, conducted decomposition analyses, and performed cross-country health inequality analyses that account for the sociodemographic index (SDI) to gain a deeper understanding of the global burden of UCAs. Through this rigorous exploration, our work seeks to reveal the scale and intricacies of UCAs worldwide, providing evidence to inform healthcare decisions and contributing significantly to the existing body of knowledge. This cross-sectional study has been reported in line with the STROCSS guidelines (11).

## Methods

### Research population and data compilation

Data on the UCAs burden from 1990 to 2021 were obtained from the Global Health Data Exchange GBD Results Tool (http://ghdx.healthdata.org/gbd-results-tool) (date of data extraction, November 12, 2024). In this study, UCAs were defined as any live births with a urinary or genital condition, including congenital malformation of the collecting system, ureter, bladder, and kidney; bladder exstrophy and epispadias; hypospadias; ambiguous or indeterminate sex; and other genital malformations according to GBD (https://www.healthdata.org).

Our study focused on four key metrics: prevalence, incidence, mortality, and DALYs associated with UCAs. In accordance with the GBD framework, 95% uncertainty intervals (UIs) were computed for all final estimates as the 2.5th and 97.5th percentiles values of 500 or 1,000 draws. The analysis covered the time span from 1990 to 2021, including annual statistics across different age groups, sexes, and regions. Our study encompassed 204 countries and territories, grouped into 21 GBD regions based on geographic proximity and further categorized into five categories according to the socio-demographic index (SDI).

### SDI

The SDI is a composite metric developed by GBD researchers to assess the socio-economic status of a region. It integrates per capita income, educational attainment, and fertility rates into a single index ranging from 0 to 1, reflecting the socio-economic health and progress of a region or country (4, 12). Higher SDI values correspond to better socio-economic conditions and improved health outcomes. Based on SDI values, regions are divided into five quintiles: low-SDI, low-middle-SDI, middle-SDI, high-middle-SDI, and high-SDI.

### Data analysis

Our data analysis began with an exploration of the dataset's structure, calculating counts and rates for key measures such as prevalence, incidence, mortality, and DALYs of UCAs at the global, regional, and national levels. We then analyzed changes in these metrics across different regions from 1990 to 2021. To determine relative changes, we used the formula:

Relativechange(%)=[(Valuein2021−Valuein1990)/Valuein1990]×100%.

This formula was applied to both case numbers and age-standardized rates (ASRs) per 100,000 population (13).

To analyze trends in UCAs, we used statistical methods, including annual percentage change (APC) to assess year-to-year variations and average annual percentage change (AAPC) to capture average trend over specified periods (14, 15).

Significant changes in data trends over time were identified using joinpoint regression analysis, which differentiates genuine trends from random fluctuations. This method uses statistical criteria to determine the minimum number of linear segments required to describe a trend. The software calculates the APC for each segment, as well as the AAPC over the entire study period, along with the corresponding 95% CIs, allowing us to assess the direction, magnitude, and significance of the observed trends. Pearson correlation was applied to explore the relationship between the SDI and UCAs indicators, assessing the strength and direction of these relationships across regions.

Decomposition analysis was performed to examine the underlying factors contributing to changes in UCAs epidemiology over the defined time periods at the global, regional and national levels (8). Changes in prevalence, mortality, and DALYs due to UCAs were decomposed into population, aging and epidemiological change. This allowed for a quantified understanding of the drivers behind these trends.

To benchmark UCAs burdens, frontier analysis compared the performance of countries and regions against the best-performing counterparts, identifying gaps and opportunities for improvement (9). We calculated the “effective difference,” representing the disparity between observed and potential burden, while accounting for SDI.

Health inequalities in UCAs burdens were assessed using the slope index of inequality and the concentration index of health inequality (10). Regression analyses were performed with incidence, prevalence, mortality and DALYs as dependent variables and socioeconomic status (defined as the midpoint of the cumulative class interval of the population sorted by gross domestic product per capita) as the independent variable. Heteroskedasticity was calculated using a weighted regression model. The concentration index of health inequality was calculated by fitting a Lorenz curve, with the area under the curve reflecting the degree of concentration of health burden among income groups. Statistical analyses were executed using R version 4.3.1.

## Results

### Global trends

From 1990 to 2021, the global UCAs prevalence increased from 5.22 million to 6.34 million, representing a 21% rise in number (Table 1). The age-standardized prevalence rates also increased, from 86.34 per 100,000 to 89.76 per 100,000, a growth of 3.96%. However, the global incidence decreased from 1.16 million to 1.09 million, reflecting a 6.25% absolute reduction (Table 2). The age-standardized incidence rate declined from 18.21 per 100,000 to 17.49 per 100,000, a 2.86% decrease. Mortality dropped by 22.56%, with the age-standardized mortality rate falling by 20% (Table 3). Similarly, DALYs, a measure of global disease burden, decreased by 15.44% in absolute numbers, with age-standardized DALYs dropping by 18.46% (Table 4).

**Table 1:** Numbers and ASRs per 100,000 cases of prevalence of urogenital congenital anomalies in 1990 and 2021, along with the relative changes and AAPC in ASRs per 100 000 cases from 1990 to 2021, categorized by global, SDI, and GBD regions.

Characteristic	Number in 1990 (95% UI)	Age-standardized rate in 1990 (95% UI)	Number in 2021 (95% UI)	Age-standardized rate in 2021 (95% UI)	Relative change of numbers from 1990 to 2021 (%)	Relative change of age-standardized rate from 1990 to 2021 (%)	AAPC (age-standardized rate, 95% CI)	*p*
Global	5,221,076 (4,188,738, 6,516,684)	86.34 (69.25, 107.63)	6,343,413 (5,069,614 7,900,494)	89.76 (71.86, 111.25)	0.21	0.04	0.13 (0.11 to 0.15)	<0.001
High SDI	547,268 (449,255, 662,805)	78.01 (63.67, 94.73)	531,934 (437,031, 640,507)	78.44 (64.2, 94.52)	−0.03	0.01	0.07 (−0.01 to 0.15)	0.068
High-middle SDI	833,380 (669,798, 1,021,852)	83.76 (67.68, 102.54)	677368 (549,598, 825,121)	78.53 (63.84, 96.03)	−0.19	−0.06	−0.21 (−0.23 to −0.18)	<0.001
Middle SDI	1,464,798 (1,178,877, 1,819,355)	73.05 (58.83, 90.63)	1,540,119 (1,232,248, 1,915,869)	76.46 (61.36, 94.62)	0.05	0.05	0.15 (0.10 to 0.21)	<0.001
Low-middle SDI	1,543,625 (1,207,293, 1,961,597)	98.13 (77.41, 123.85)	2,006,134 (1,580,291, 2,553,132)	100.02 (79.12, 126.79)	0.3	0.02	0.06 (0.04 to 0.08)	<0.001
Low SDI	828,395 (649,878, 1,043,574)	108.34 (85.93, 135.7)	1,583,659 (1,246,162, 1,997,216)	105.02 (82.63, 131.94)	0.91	−0.03	−0.09 (−0.11 to −0.08)	<0.001
Andean Latin America	24,720 (20,047, 29,929)	50.03 (40.57, 60.5)	38,156 (31,238 46157)	59.05 (48.15, 71.57)	0.54	0.18	0.54 (0.52 to 0.57)	<0.001
Australasia	13948 (11268,17193)	82.05 (66.09, 101.33)	18134 (14724,21927)	84.92 (68.82, 102.91)	0.3	0.03	0.09 (−0.12 to 0.30)	0.416
Caribbean	25,342 (20,453, 30,575)	62.94 (50.96, 75.86)	28,196 (22,799, 33,983)	67.48 (54.4, 81.58)	0.11	0.07	0.23 (0.21 to 0.25)	<0.001
Central Asia	72,192 (57,227, 88,565)	83.78 (66.84, 102.87)	88,449 (70,461, 108,127)	90.9 (72.32, 111.45)	0.23	0.08	0.27 (0.23 to 0.30)	<0.001
Central Europe	78,463 (62,077, 97,394)	76.67 (60.93, 95.24)	54734 (44018,66750)	80.9 (65.11, 98.93)	−0.3	0.06	0.17 (0.13 to 0.22)	<0.001
Central Latin America	175,178 (141,285, 212,812)	81.68 (65.95, 98.61)	187,651 (154,501,b223,340)	82.33 (67.56, 97.96)	0.07	0.01	0.02 (0.01 to 0.04)	0.01
Central Sub-Saharan Africa	110,343 (85,919, 138,585)	128.95 (100.73, 161.65)	239113 (185,957,n305,249)	126.65 (99.95, 161.45)	1.17	−0.02	−0.05 (−0.20 to 0.10)	0.481
East Asia	721.````572 (567,859, 882,490)	58.98 (46.51, 72.09)	486,107 (390,781, 597,275)	51.5 (41.37, 63.64)	−0.33	−0.13	−0.43 (−0.50 to −0.37)	<0.001
Eastern Europe	260,259 (209,346, 321,115)	139.53 (111.98, 172.52)	167,491 (135,239, 204,944)	127.49 (102.38, 155.45)	−0.36	−0.09	−0.30 (−0.35 to −0.25)	<0.001
Eastern Sub-Saharan Africa	359,857 (283,078, 451,715)	119.28 (95.39, 148.23)	649,125 (509,242, 819,187)	111.03 (87.81, 139.3)	0.8	−0.07	−0.23 (−0.29 to −0.18)	<0.001
High-income Asia Pacific	139,339 (110,408, 173,153)	107.55 (85.47, 132.49)	98,358 (79,789, 121,119)	107.25 (86.3, 132.07)	−0.29	0	−0.01 (−0.04 to 0.02)	0.683
High-income North America	148,100 (120,727, 176,691)	63 (51.37, 75.18)	155,033 (126,063, 186,131)	62.51 (50.64, 75.45)	0.05	−0.01	0.04 (−0.28 to 0.37)	0.794
North Africa and Middle East	463,111 (364,161, 585,729)	100.25 (79.16, 126.43)	639,153 (507,974, 803,575)	100.25 (79.82, 126.38)	0.38	0	−0.00 (−0.03 to 0.03)	0.806
Oceania	6,549 (5,137, 8,184)	72.69 (57.34, 91.71)	15,036 (11,777, 19,232)	85.83 (67.29, 110.21)	1.3	0.18	0.53 (0.49 to 0.57)	<0.001
South Asia	1,607,537 (1,241,699, 2,056,850)	111.46 (86.37, 141.96)	1,996,136 (1,555,342, 2,568,175)	111.97 (87.39, 143.43)	0.24	0	0.02 (0.00 to 0.03)	0.014
Southeast Asia	261,398 (204,418, 332,386)	45.31 (35.45, 57.41)	297,511 (233,395, 375,698)	48.11 (37.7, 60.46)	0.14	0.06	0.19 (0.16 to 0.22)	<0.001
Southern Latin America	48,933 (39,433, 59,651)	95.47 (77.11, 116.17)	57,171 (45,680, 70,151)	106.11 (85.69, 130.22)	0.17	0.11	0.36 (0.32 to 0.39)	<0.001
Southern Sub-Saharan Africa	103,915 (82,504, 132,003)	151.8 (121.21, 191.56)	137,330 (107,872, 172,735)	164.25 (129.29, 206.02)	0.32	0.08	0.24 (0.18 to 0.30)	<0.001
Tropical Latin America	97,787 (78,700, 119,057)	55.42 (44.63, 67.23)	105,221 (85,036, 126,193)	55.63 (44.71, 67.14)	0.08	0	0.02 (−0.04 to 0.08)	0.443
Western Europe	214,114 (175,751, 258,880)	77.11 (63.59, 93.24)	207,749 (170,498, 250,401)	78.66 (64.92, 95.24)	−0.03	0.02	0.06 (0.03 to 0.08)	<0.001
Western Sub-Saharan Africa	288,419 (229,374, 358,437)	96.48 (77.58, 118.95)	677,557 (538,492, 841,523)	96.2 (76.79, 119.34)	1.35	0	−0.01 (−0.03 to 0.02)	0.653

**Table 2:** Numbers and ASRs per 100,000 cases of incidence of urogenital congenital anomalies in 1990 and 2021, along with the relative changes and AAPC in ASRs per 100 000 cases from 1990 to 2021, categorized by global, SDI, and GBD regions.

Characteristic	Number in 1990 (95% UI)	Age-standardized rate in 1990 (95% UI)	Number in 2021 (95% UI)	Age-standardized rate in 2021 (95% UI)	Relative change of numbers from 1990 to 2021 (%)	Relative change of age-standardized rate from 1990 to 2021 (%)	AAPC (age-standardized rate, 95% CI)	*p*
Global	11,67,139 (887,687, 1,554,170)	18.21 (13.85, 24.25)	10,94,236 (823,143, 1,438,205)	17.69 (13.31, 23.25)	−0.06	−0.03	−0.08 (−0.16 to 0.00)	0.053
High SDI	92,805 (72,523, 118,254)	15.44 (12.07, 19.67)	71,235 (54,782, 91,380)	14.4 (11.07, 18.47)	−0.23	−0.07	−0.15 (−0.33 to 0.04)	0.13
High-middle SDI	145,347 (110273, 189,578)	16.56 (12.56, 21.6)	82,520 (62,048, 108,329)	14.67 (11.03, 19.26)	−0.43	−0.11	−0.38 (−0.50 to −0.27)	<0.001
Middle SDI	323,762 (244,056,v428,023)	16.16 (12.18, 21.36)	238,029 (176,352, 314,091)	15.55 (11.52, 20.52)	−0.26	−0.04	−0.10 (−0.20 to 0.00)	0.055
Low-middle SDI	371,688 (279,457, 497,608)	20 (15.04, 26.77)	359,652 (268,523, 481,250)	19.28 (14.39, 25.79)	−0.03	−0.04	−0.12 (−0.19 to −0.06)	<0.001
Low SDI	232,774 (175,112, 315,309)	21.92 (16.49, 29.69)	342,073 (256,408, 453,936)	19.8 (14.84, 26.27)	0.47	−0.1	−0.32 (−0.37 to −0.27)	<0.001
Andean Latin America	6,031 (4,562, 7,857)	10.71 (8.1, 13.96)	7,318 (5,491, 9654)	12.31 (9.24, 16.24)	0.21	0.15	0.45 (0.38 to 0.51)	<0.001
Australasia	2,805 (2,211, 3655)	18.38 (14.49, 23.95)	2759 (1,985, 3,751)	16.04 (11.54, 21.81)	−0.02	−0.13	−0.37 (−0.52 to −0.22)	<0.001
Caribbean	5,716 (4,295, 7554)	13.23 (9.94, 17.49)	4,909 (3,625, 6,578)	12.87 (9.5, 17.25)	−0.14	−0.03	−0.07 (−0.16 to 0.02)	0.108
Central Asia	15,984 (11,571, 21,400)	16.94 (12.26, 22.67)	1,7674 (12,773, 23,656)	17.99 (13.01, 24.09)	0.11	0.06	0.18 (0.14 to 0.22)	<0.001
Central Europe	13,572 (10,363, 17,716)	16.44 (12.56, 21.46)	6,855 (5,097, 8,941)	13.62 (10.13, 17.77)	−0.49	−0.17	−0.60 (−0.64 to −0.57)	<0.001
Central Latin America	39,972 (29,083, 52,209)	16.68 (12.14, 21.79)	28,541 (21,122, 37,196)	15.23 (11.27, 19.85)	−0.29	−0.09	−0.28 (−0.34 to −0.23)	<0.001
Central Sub-Saharan Africa	32,484 (24,225, 43,171)	26.36 (19.66, 35.04)	52,943 (39,030, 72,252)	24.77 (18.26, 33.8)	0.63	−0.06	−0.21 (−0.25 to −0.16)	<0.001
East Asia	161,164 (119,842, 212,867)	14.05 (10.45, 18.56)	60,774 (44,055, 82,158)	11.02 (7.99, 14.89)	−0.62	−0.22	−0.73 (−0.92 to −0.55)	<0.001
Eastern Europe	35,777 (26,605, 47961)	24.93 (18.54, 33.41)	18,731 (13,470, 25,167)	21.72 (15.62, 29.18)	−0.48	−0.13	−0.42 (−0.51 to −0.33)	<0.001
Eastern Sub-Saharan Africa	104,536 (77,987, 143,182)	24.3 (18.13, 33.29)	137,987 (103,037, 183,612)	21.03 (15.7, 27.99)	0.32	−0.13	−0.44 (−0.57 to −0.31)	<0.001
High-income Asia Pacific	20,622 (15,675, 27,390)	21.72 (16.51, 28.84)	9,832 (7,473, 12,990)	17.21 (13.08, 22.73)	−0.52	−0.21	−0.73 (−0.85 to −0.62)	<0.001
High-income North America	28,153 (21,237, 36,556)	12.82 (9.67, 16.64)	26,095 (19,243, 34,853)	13.39 (9.87, 17.88)	−0.07	0.04	0.39 (−0.24 to 1.02)	0.222
North Africa and Middle East	102,879 (78,650, 136,656)	19.58 (14.97, 26.01)	86,214 (65,312, 111,016)	15.07 (11.42, 19.4)	−0.16	−0.23	−0.84 (−0.88 to −0.79)	<0.001
Oceania	1,611 (1,197, 2,165)	14.94 (11.1, 20.09)	3,565 (2,675, 4,848)	17.36 (13.03, 23.61)	1.21	0.16	0.48 (0.45 to 0.52)	<0.001
South Asia	368,030 (276,628, 496,090)	22.33 (16.78, 30.09)	338,108 (247,792, 456,233)	22.33 (16.37, 30.14)	−0.08	0	0.02 (−0.11 to 0.15)	0.753
Southeast Asia	61,655 (46,190, 83,609)	10.4 (7.79, 14.11)	53,439 (39,490, 71,738)	9.91 (7.32, 13.3)	−0.13	−0.05	−0.16 (−0.18 to −0.13)	<0.001
Southern Latin America	8,359 (6,321, 11,120)	16.48 (12.46, 21.93)	6,746 (5,059, 8,890)	18.12 (13.59, 23.88)	−0.19	0.1	0.29 (0.15 to 0.43)	<0.001
Southern Sub-Saharan Africa	21,313 (15,645, 28,478)	27.43 (20.14, 36.65)	22,210 (16,443, 30,376)	28.41 (21.04, 38.86)	0.04	0.04	0.14 (−0.00 to 0.28)	0.056
Tropical Latin America	20,971 (15,607, 27,338)	13.01 (9.69, 16.97)	20,205 (1,5186, 26,827)	12.22 (9.19, 16.23)	−0.04	−0.06	−0.17 (−0.38 to 0.04)	0.116
Western Europe	30,944 (25,394, 37,943)	13.91 (11.41, 17.05)	27,549 (22,277, 33,949)	13.98 (11.3, 17.22)	−0.11	0.01	0.01 (−0.02 to 0.05)	0.454
Western Sub-Saharan Africa	84,562 (63,473, 112,350)	19.72 (14.8, 26.2)	161,783 (120,590, 213,250)	18.99 (14.15, 25.03)	0.91	−0.04	−0.14 (−0.26 to −0.01)	0.034

**Table 3:** Numbers and ASRs per 100,000 cases of deaths of urogenital congenital anomalies in 1990 and 2021, along with the relative changes and AAPC in ASRs per 100,000 cases from 1990 to 2021, categorized by global, SDI, and GBD regions.

Characteristic	Number in 1990 (95% UI)	Age-standardized rate in 1990 (95% UI)	Number in 2021 (95% UI)	Age-standardized rate in 2021 (95% UI)	Relative change of numbers from 1990 to 2021 (%)	Relative change of age-standardized rate from 1990 to 2021 (%)	AAPC (age-standardized rate, 95% CI)	*p*
Global	96,79 (6,857, 15,280)	0.15 (0.11, 0.24)	7,495 (5,175, 11,712)	0.12 (0.08, 0.19)	−0.22564	−0.2	−0.86 (−0.99 to −0.74)	<0.001
High SDI	1,020 (7,911,433)	0.17 (0.13, 0.23)	498 (335, 652)	0.09 (0.06, 0.12)	−0.51176	−0.47059	−1.84 (−2.06 to −1.63)	<0.001
High-middle SDI	1,333 (911, 2,022)	0.15 (0.1,0.23)	462 (344, 620)	0.07 (0.05, 0.1)	−0.65341	−0.53333	−2.29 (−2.59 to −1.99)	<0.001
Middle SDI	2,444 (1,841, 3,508)	0.12 (0.09, 0.18)	1,745 (1,240, 2,297)	0.1 (0.07, 0.14)	−0.28601	−0.16667	−0.57 (−0.83 to −0.31)	<0.001
Low-middle SDI	3,429 (1,833, 5,616)	0.19 (0.1,0.31)	2,816 (1,600, 4,897)	0.15 (0.09, 0.26)	−0.17877	−0.21053	−0.77 (−0.91 to −0.63)	<0.001
Low SDI	1,447 (782, 3272)	0.15 (0.08, 0.33)	1968 (1,162, 3,952)	0.12 (0.07, 0.23)	0.360055	−0.2	−0.67 (−0.74 to −0.59)	<0.001
Andean Latin America	81 (43,147)	0.15 (0.08, 0.27)	83 (46, 146)	0.14 (0.08, 0.24)	0.024691	−0.06667	−0.19 (−0.59 to 0.21)	0.349
Australasia	24 (15, 51)	0.15 (0.09, 0.33)	13 (9, 27)	0.07 (0.04, 0.15)	−0.45833	−0.53333	−2.61 (−3.63 to −1.58)	<0.001
Caribbean	68 (42, 113)	0.16 (0.1,0.27)	52 (28, 118)	0.13 (0.07, 0.3)	−0.23529	−0.1875	−0.67 (−0.87 to −0.46)	<0.001
Central Asia	58 (40,82)	0.06 (0.04, 0.09)	52 (36,76)	0.05 (0.04, 0.08)	−0.10345	−0.16667	−0.52 (−0.97 to −0.07)	0.023
Central Europe	132 (74, 198)	0.16 (0.09, 0.24)	38 (20, 52)	0.07 (0.04, 0.1)	−0.71212	−0.5625	−2.55 (−3.09 to −2.01)	<0.001
Central Latin America	515 (386, 853)	0.23 (0.17, 0.38)	581 (405, 743)	0.27 (0.19, 0.35)	0.128155	0.173913	0.59 (0.35 to 0.84)	<0.001
Central Sub-Saharan Africa	120 (43, 337)	0.1 (0.04, 0.29)	160 (59, 454)	0.08 (0.03, 0.22)	0.333333	−0.2	−0.93 (−1.03 to −0.82)	<0.001
East Asia	280 (94, 677)	0.02 (0.01, 0.06)	133 (54, 209)	0.01 (0, 0.03)	−0.525	−0.5	−1.76 (−2.30 to −1.22)	<0.001
Eastern Europe	203 (136, 266)	0.13 (0.08, 0.17)	58 (38, 98)	0.05 (0.03, 0.08)	−0.71429	−0.61538	−3.32 (−3.80 to −2.84)	<0.001
Eastern Sub-Saharan Africa	382 (155, 1275)	0.1 (0.04, 0.32)	473 (195, 1173)	0.07 (0.03, 0.18)	0.23822	−0.3	−0.85 (−0.90 to −0.80)	<0.001
High-income Asia Pacific	137 (93, 180)	0.14 (0.09, 0.19)	35 (21, 58)	0.06 (0.04, 0.09)	−0.74453	−0.57143	−2.83 (−3.51 to −2.14)	<0.001
High-income North America	451 (331, 634)	0.2 (0.15, 0.29)	258 (179, 341)	0.13 (0.09, 0.17)	−0.42794	−0.35	−1.47 (−1.87 to −1.06)	<0.001
North Africa and Middle East	2,646 (1,396, 4,752)	0.51 (0.27, 0.92)	1,363 (852, 2,157)	0.23 (0.15, 0.37)	−0.48488	−0.54902	−2.52 (−2.63 to −2.40)	<0.001
Oceania	5 (1, 18)	0.04 (0.01, 0.17)	20 (4, 55)	0.1 (0.02, 0.27)	3	1.5	2.67 (2.52 to 2.81)	<0.001
South Asia	3,072 (1,358, 5,258)	0.19 (0.09, 0.33)	2,439 (1,060, 4,871)	0.16 (0.07, 0.32)	−0.20605	−0.15789	−0.61 (−0.81 to −0.40)	<0.001
Southeast Asia	265 (147, 632)	0.05 (0.03, 0.11)	169 (100,,333)	0.03 (0.02, 0.06)	−0.36226	−0.4	−1.45 (−1.64 to −1.26)	<0.001
Southern Latin America	138 (100, 227)	0.27 (0.2,0.45)	95 (54, 132)	0.25 (0.14, 0.35)	−0.31159	−0.07407	−0.23 (−0.81 to 0.36)	0.446
Southern Sub-Saharan Africa	35 (22, 55)	0.05 (0.03, 0.07)	44 (22, 73)	0.06 (0.03, 0.09)	0.257143	0.2	0.49 (0.20 to 0.78)	<0.001
Tropical Latin America	312 (217, 546)	0.19 (0.13, 0.34)	380 (196, 509)	0.22 (0.12, 0.3)	0.217949	0.157895	0.46 (0.26 to 0.66)	<0.001
Western Europe	340 (240, 547)	0.15 (0.1,0.24)	165 (104, 217)	0.08 (0.05, 0.1)	−0.51471	−0.46667	−2.08 (−2.26 to −1.89)	<0.001
Western Sub-Saharan Africa	416 (166, 1181)	0.11 (0.05, 0.31)	885 (447, 2,123)	0.11 (0.06, 0.26)	1.127404	0	0.12 (0.04 to 0.20)	0.004

**Table 4:** Numbers and ASRs per 100,000 cases of DALYs of urogenital congenital anomalies in 1990 and 2021, along with the relative changes and AAPC in ASRs per 100 000 cases from 1990 to 2021, categorized by global, SDI, and GBD regions.

Characteristic	Number in 1990 (95% UI)	Age-standardized rate in 1990 (95% UI）	Number in 2021 (95% UI）	Age-standardized rate in 2021 (95% UI）	Relative change of numbers from 1990 to 2021 (%)	Relative change of age-standardized rate from 1990 to 2021 (%)	AAPC (age-standardized rate, 95% CI)	*p*
Global	1,043,302 (775,258, 1,567,968)	16.63 (12.38, 24.89)	882,185 (659,007, 1,258,015)	13.56 (10.08, 19.57)	−0.15	−0.18	−0.63 (−0.68 to −0.59)	<0.001
High SDI	108,352 (84,999, 144,909)	17.35 (13.64, 23.33)	60,842 (44,194, 77,576)	10.88 (7.73, 13.95)	−0.44	−0.37	−1.49 (−1.66 to −1.32)	<0.001
High-middle SDI	145,490 (102,689, 207,726)	15.95 (11.23, 22.94)	62,065 (46,188, 81,511)	8.99 (6.65, 11.83)	−0.57	−0.44	−1.83 (−2.09 to −1.56)	<0.001
Middle SDI	265,771 (204,185, 367,257)	13.33 (10.24, 18.42)	201,238 (151,067, 254,356)	11.63 (8.61, 14.65)	−0.24	−0.13	−0.44 (−0.62 to −0.25)	<0.001
Low-middle SDI	363,558 (224,125, 568,826)	20.6 (12.88, 31.9)	324,373 (212,233, 519,705)	17.03 (11.07, 27.49)	−0.11	−0.17	−0.63 (−0.75 to −0.51)	<0.001
Low SDI	159,376 (100,175, 3,26,982)	16.88 (10.85, 33.81)	233,055 (155,686, 409,449)	14.26 (9.52, 24.57)	0.46	−0.16	−0.53 (−0.58 to −0.48)	<0.001
Andean Latin America	7,934 (4,653, 14,003)	14.57 (8.6, 25.6)	8,368 (5,062, 14,204)	13.78 (8.3, 23.53)	0.05	−0.05	−0.12 (−0.47 to 0.24)	0.516
Australasia	2,539 (1,673, 4,914)	16.12 (10.58, 31.66)	1,638 (1,154, 2,890)	8.49 (5.94, 15.64)	−0.35	−0.47	−1.84 (−2.77 to −0.90)	<0.001
Caribbean	6,752 (4,424, 10,667)	16.05 (10.53, 25.19)	5,343 (3,225, 10,928)	13.56 (8.12, 27.83)	−0.21	−0.16	−0.53 (−0.71 to −0.35)	<0.001
Central Asia	7,350 (5,331, 9,805)	8.13 (5.92, 10.85)	7,413 (5,500, 10,303)	7.56 (5.6, 10.52)	0.01	−0.07	−0.19 (−0.47 to 0.09)	0.18
Central Europe	14,550 (9,379, 20,602)	16.73 (10.39, 23.91)	5,279 (3,324, 7,232)	9.27 (5.7, 12.77)	−0.64	−0.45	−1.91 (−2.32 to −1.50)	<0.001
Central Latin America	49,363 (37,922, 79,785)	21.66 (16.74, 34.78)	51542 (37,553, 65,316)	24.61 (17.78, 31.68)	0.04	0.14	0.45 (0.24 to 0.65)	<0.001
Central Sub-Saharan Africa	1,4807 (7,718, 35,592)	14.06 (7.87, 32.35)	23,162 (12,297, 47,502)	11.57 (6.24, 23.19)	0.56	−0.18	−0.60 (−0.69 to −0.50)	<0.001
East Asia	46,192 (25,887, 85,096)	3.87 (2.16, 7.15)	24,665 (15,895, 37,561)	2.77 (1.65, 4.35)	−0.47	−0.28	−1.10 (−1.35 to −0.86)	<0.001
Eastern Europe	26,136 (19,855 33,112)	15.85 (12.06, 20.29)	9,945 (7,037, 13,900)	8.08 (5.55, 11.55)	−0.62	−0.49	−2.20 (−2.58 to −1.82)	<0.001
Eastern Sub-Saharan Africa	4,7217 (25,920, 127,123)	12.92 (7.45, 32.75)	65,637 (36,824, 124,935)	10.62 (6.08, 19.79)	0.39	−0.18	−0.64 (−0.69 to −0.59)	<0.001
High-income Asia Pacific	17,260 (12,929, 22,107)	16.5 (12.19, 20.88)	6,705 (4,572, 9,286)	9.06 (6.25, 12.41)	−0.61	−0.45	−1.92 (−2.36 to −1.48)	<0.001
High-income North America	44,622 (32,882, 60,169)	20.04 (14.77, 27.11)	27,252 (19,660, 34,052)	13.1 (9.36, 16.59)	−0.39	−0.35	−1.31 (−1.68 to −0.95)	<0.001
North Africa and Middle East	254,441 (140,904, 445,538)	49.58 (27.49, 86.44)	146,040 (98,315, 216,102)	24.73 (16.63, 36.79)	−0.43	−0.5	−2.25 (−2.35 to −2.16)	<0.001
Oceania	649 (281, 1,878)	6.64 (3.14, 18.22)	2,361 (854, 5,397)	12.15 (4.68, 26.94)	2.64	0.83	1.98 (1.88 to 2.08)	<0.001
South Asia	335,848 (178,417, 535,430)	21.3 (11.51, 33.66)	293,670 (158,751, 518,527)	18.42 (9.73, 33.33)	−0.13	−0.14	−0.47 (−0.64 to −0.31)	<0.001
Southeast Asia	32,730 (21,382, 65,471)	5.64 (3.7, 11.22)	25,045 (16,437, 39,485)	4.27 (2.79, 6.85)	−0.23	−0.24	−0.88 (−1.05 to −0.72)	<0.001
Southern Latin America	14005 (10127,21878)	27.57 (19.93, 43.1)	10,449 (6443, 13,917)	26.21 (15.84, 35.22)	−0.25	−0.05	−0.13 (−0.65 to 0.39)	0.614
Southern Sub-Saharan Africa	6,941 (4,850, 9,720)	9.82 (6.82, 13.85)	8,950 (6,073, 12,801)	10.97 (7.51, 15.64)	0.29	0.12	0.37 (0.19 to 0.55)	<0.001
Tropical Latin America	30,586 (21,448, 51,469)	18.8 (13.16, 31.76)	36,217 (19,833, 47,961)	21.41 (11.62, 28.43)	0.18	0.14	0.39 (0.22 to 0.57)	<0.001
Western Europe	36,554 (26,369, 54,929)	15.44 (11.11, 23.44)	20,875 (14,896, 27,276)	9.32 (6.49, 12.15)	−0.43	−0.4	−1.70 (−1.94 to −1.45)	<0.001
Western Sub-Saharan Africa	46,825 (24,028, 114,170)	12.68 (6.98, 29.64)	101,631 (61,457, 207,289)	12.94 (7.97, 25.49)	1.17	0.02	0.07 (−0.02 to 0.16)	0.106

**Figure 1:**
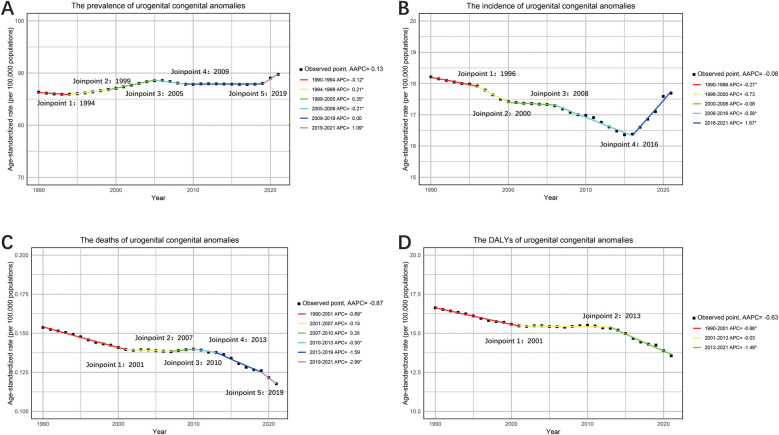
The APC and AAPC of ASR for prevalence **(A)**, incidence **(B)**, deaths **(C)** and DALYs **(D)** in urogenital congenital anomalies at the global level based on the joinpoint regression analysis model.

Further analysis using APC and AAPC revealed varying trends. Prevalence showed notable increases during 1999–2005 and 2019–2021, with APC values of 0.35% (95% CI: 0.32%, 0.39%) and 1.09% (95% CI: 0.95%, 1.23%), respectively, and an overall AAPC of 0.13% (95% CI: 0.11%, 0.15%) (Figure 1A). Incidence exhibited a declining trend from 1990 to 2016, with the largest decrease between 2006 and 2016 (APC: −0.58%; 95% CI: −0.66%, −0.50%), but it increased from 2016 to 2021 (APC: 1.67%; 95% CI: 1.46%, 1.88%), resulting in an overall AAPC of −0.08% (95% CI: −0.16%, 0.00%) (Figure 1B). Mortality demonstrated a consistent downward trend from 1990 to 2021, with a slight increase during 2007–2010 (APC: 0.35%; 95% CI: −0.44%, 1.14%), yielding an overall AAPC of −0.86% (95% CI: −0.98%, −0.74%) (Figure 1C). DALYs also declined steadily over the period, with a notable reduction between 2013 and 2021 (APC: −1.49%; 95% CI: −1.49%, −1.61%) and an overall AAPC of −0.63% (95% CI: −0.68%, −0.58%) (Figure 1D).

### Global trends by sex

**Figure 2:**
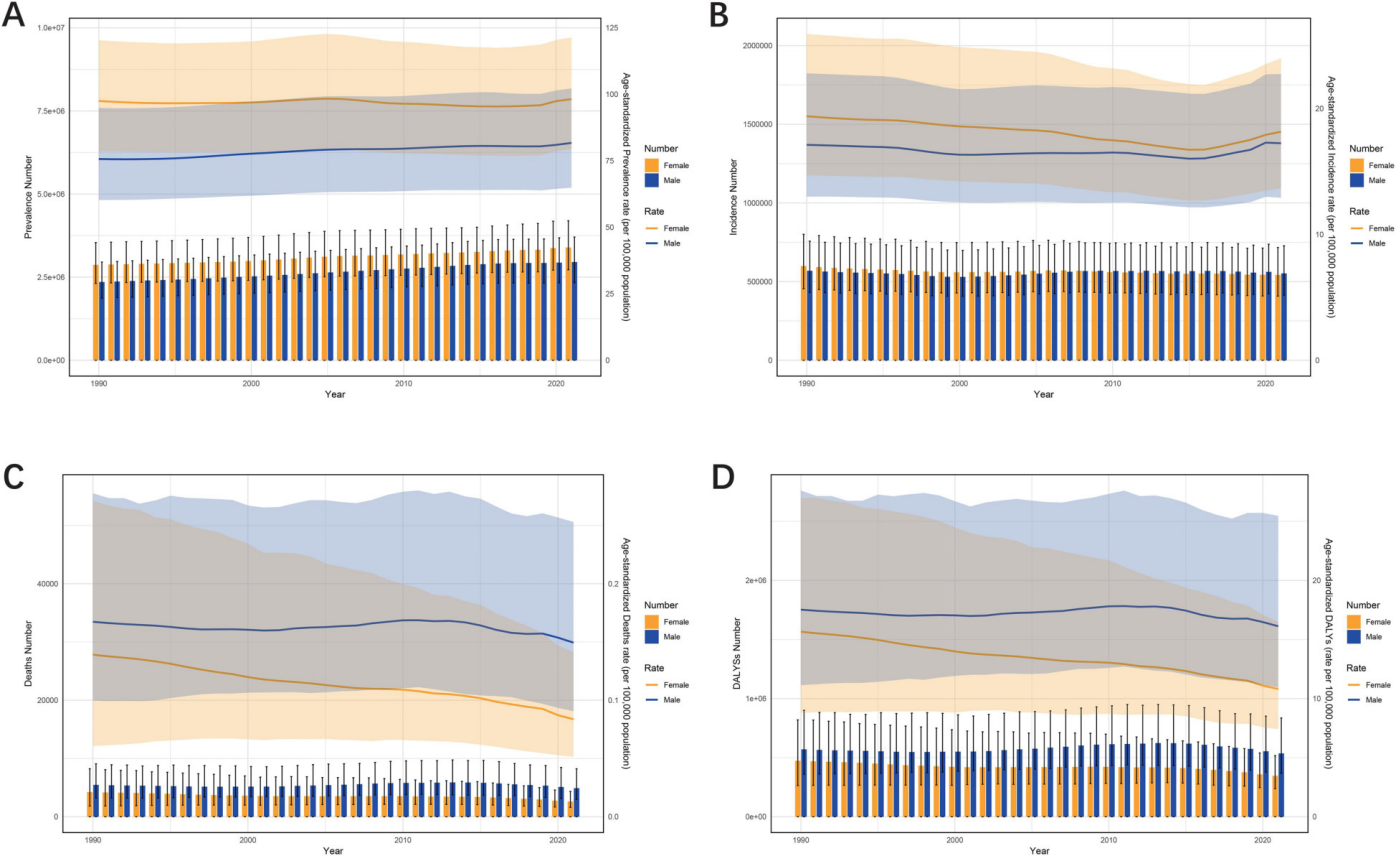
The trends in numbers and ASR of prevalence **(A)**, incidence **(B)**, deaths **(C)**, and DALYs **(D)** for urogenital congenital anomalies hernias among different sexes, female and male, from 1990 to 2021.

Over the past three decades, ASRs indicate that males consistently had higher rates in mortality and DALYs, while females had higher rates in prevalence and incidence. However, when focusing on absolute numbers, the incidence among males gradually surpassed that of females (Figures 2A–D).

**Figure 3:**
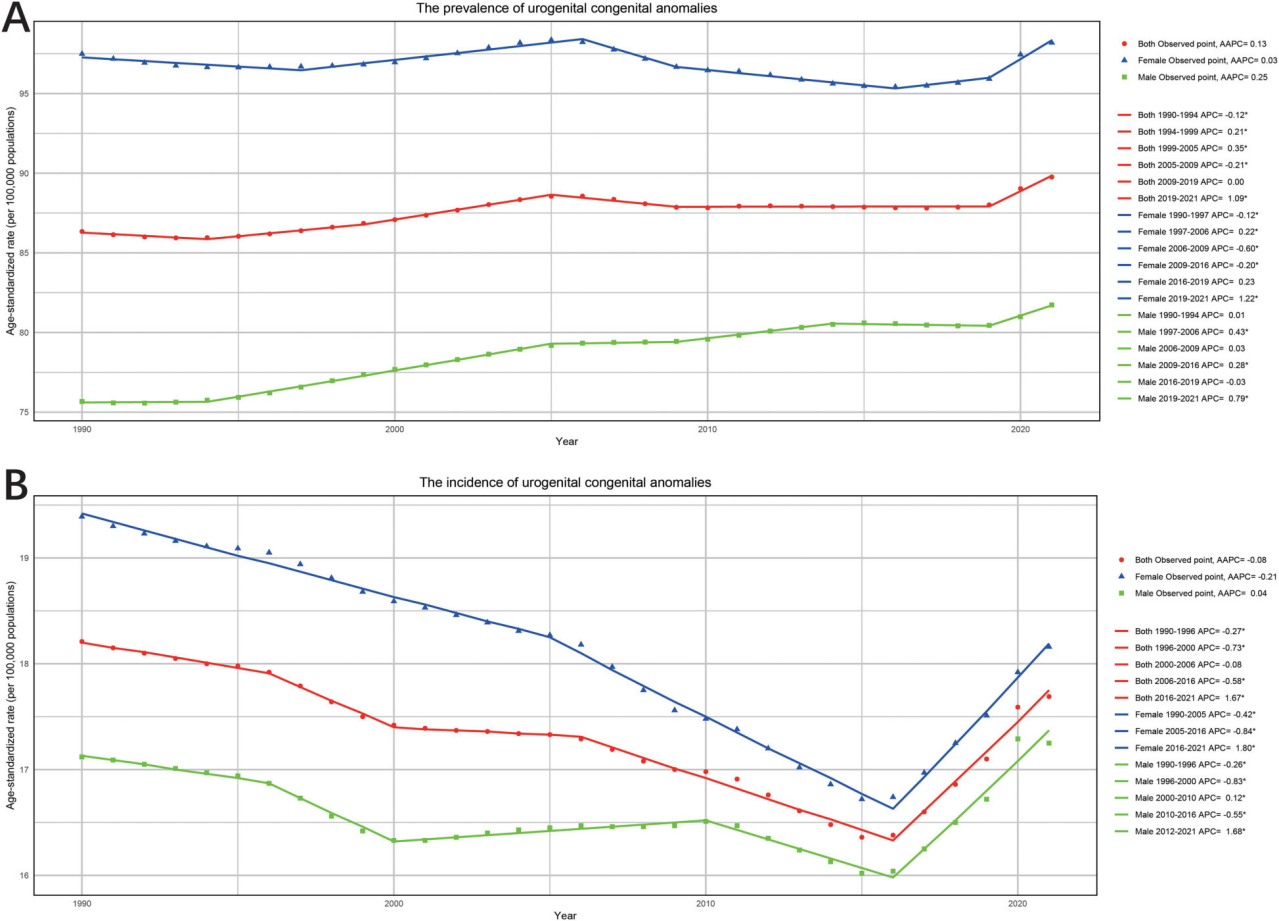
The APC and AAPC of ASR for prevalence **(A)** and incidence **(B)** by gender (both, female and male) in urogenital congenital anomalies at the global level based on the joinpoint regression analysis model.

From 1990 to 2021, a gender-specific analysis of UCAs prevalence showed that prevalence in females remained relatively stable (AAPC: 0.03%; 95% CI: −0.02%, 0.88%), while prevalence in males increased significantly (AAPC: 0.25%; 95% CI: 0.23%, 0.27%) (Figure 3A). Notably, both sexes experienced a rapid increase in age-standardized prevalence rates between 2019 and 2021. Incidence rates in females declined from 1990 to 2016, with significant decreases during 1990–2005 (APC: −0.42%; 95% CI: −0.45%, −0.38%) and 2005–2016 (APC: −0.84%; 95% CI: −0.89%, −0.78%), but showed an increasing trend from 2016 to 2021 (APC: 1.8%; 95% CI: 1.63%, 1.96%) (Figure 3B). For males, incidence rates fluctuated over the 30 years, with increases during 2000–2012 and 2016–2021, and declines in other periods. Compared to 1990, male incidence showed a slight overall increase (AAPC: 0.04%; 95% CI: −0.05%, 0.13%), while female incidence declined (AAPC: −0.21%; 95% CI: −0.25%, −0.17%).

**Figure 4:**
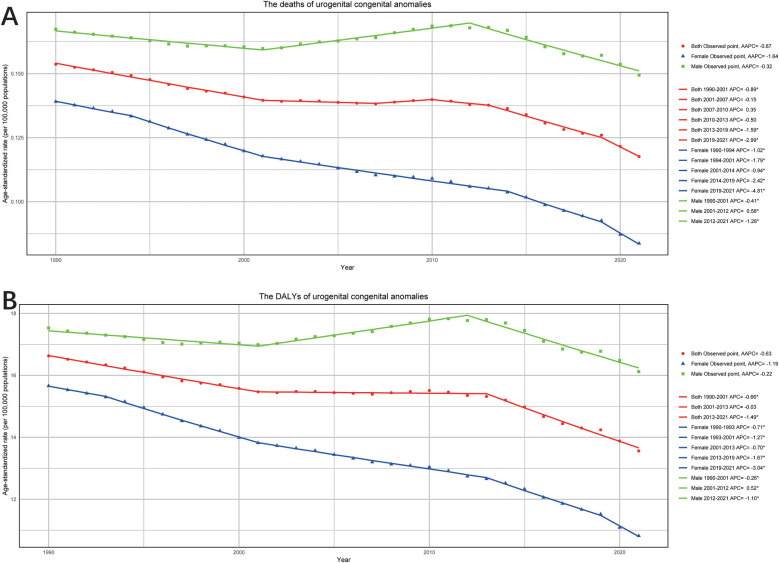
The APC and AAPC of ASR for deaths **(A)** and DALYs **(B)** by gender (both, female and male) in urogenital congenital anomalies at the global level based on the joinpoint regression analysis model.

Significant trends were also observed in ASRs for mortality and DALYs between genders. For mortality, males experienced a decline during 1990–2001(APC: −0.41%; 95% CI: −0.52%, −0.31%) and 2012–2021 (APC: −1.28%; 95% CI: −1.43%, −1.14%), but an increase during 2001–2012 (APC: 0.58%; 95% CI: 0.46%, 0.70%), with an overall AAPC of −0.31% (95% CI: −0.38%, −0.25%) (Figure 4A). In contrast, female mortality declined consistently over the 30 years, with the sharpest decline occurring from 2019 to 2021(APC: −4.82%; 95% CI: −5.73%, −3.90%), resulting in an overall AAPC of −1.63% (95% CI: −1.71%, −1.53%). Similarly, trends in DALYs mirrored this positive trajectory, with reductions observed in both sexes (Figure 4B). Females experienced the greatest decline in DALYs during 1993–2001 (APC: −1.27%; 95% CI: −1.38%, −1.16%). For males, DALYs declined during 1990–2001 (APC: −0.26%; 95% CI: −0.35%, −0.17%) and 2012–2021 (APC: −1.10%; 95% CI: −1.21%, −0.98%), but showed an upward trend from 2001 to 2012 (APC: 0.52%; 95% CI: 0.42%, 0.62%). The overall downward trend in DALYs was reflected by an AAPC of −0.23% (95% CI: −0.28%, −0.17%) for male and −1.19% (95% CI: −1.25%, −1.12%) for female.

### Regional trends

From 1990 to 2021, the prevalence of UCAs showed significant variation across 21 regions. In 2021, the ASR was observed in Southern Sub-Saharan Africa at 164.25 (95% UI: 129.29–206.02) per 100,000 cases, while the lowest was in Southeast Asia at 48.1 (95% UI: 37.7–60.46) per 100,000 cases. The ASPR in East Asia demonstrated a significant decline, with a relative change of −12.68% and an AAPC of −0.73% (95% CI: −0.54% to −0.91%). In contrast, Oceania experienced an increasing trend, with a relative change of 18.07% and an AAPC of 0.48% (95% CI: −0.45% to −0.52%). For incidence, Southern Sub-Saharan Africa also recorded the highest ASR at 28.41 (95% UI: 21.04–38.86) per 100,000 cases, while Southeast Asia had the lowest at 9.91 (95% UI: 7.32–13.3) per 100,000 cases (Tables 1, 2).

In terms of mortality, Eastern Europe experienced a substantial 61.54% reduction in ASR over the 30 years, with an AAPC of −3.32% (95% CI: −2.84% to −3.80%). Conversely, Oceania's ASR increased by 150%, with the absolute number of deaths rising by 300% and an AAPC of 2.67% (95% CI: 2.52%–2.81%). Regions such as High-Income Asia Pacific, Central Europe, and North Africa showed significant declines in both numbers of deaths and ASRs. The trends in DALYs associated with UCAs varied across regions. For example, Oceania exhibited the largest relative increase, with DALYs rising by 263.79% in numbers and 82.98% in ASR, with an AAPC of 1.98% (95% CI: 1.88% to 2.08%). In sharp contrast, Central Europe and Northern Europe experienced substantial declines in DALYs, with absolute numbers decreasing by −63.72% and −61.95%, respectively. Their AAPCs were −2.20% (95% CI: −1.82% to −2.58%) and −1.70% (95% CI: −1.45% to −1.94%), respectively (Tables 3, 4).

### National trends

From 1990 to 2021, significant changes were observed in the number of UCAs across 204 countries and territories. In terms of prevalence, 137 countries experienced an increase, with Qatar showing the most significant growth at 476.47%. Conversely, 67 countries, including the United States Virgin Islands at −54.44% and the Republic of Moldova at −55.65%, exhibited a decline. For incidence, 187 out of 204 countries showed an upward trend, with Qatar again leading the increase at 184.40%. However, Puerto Rico recorded the largest decline, at −72.19%. Mortality trends varied widely, with Papua New Guinea at 402.45% experiencing the largest increase, while Puerto Rico showed the greatest decline at −96.23%. Regarding DALYs, Papua New Guinea saw the most significant increase at 334.20%, while Puerto Rico again exhibited the largest reduction at −89.42% (Supplementary Table S1–S4).

**Figure 5:**
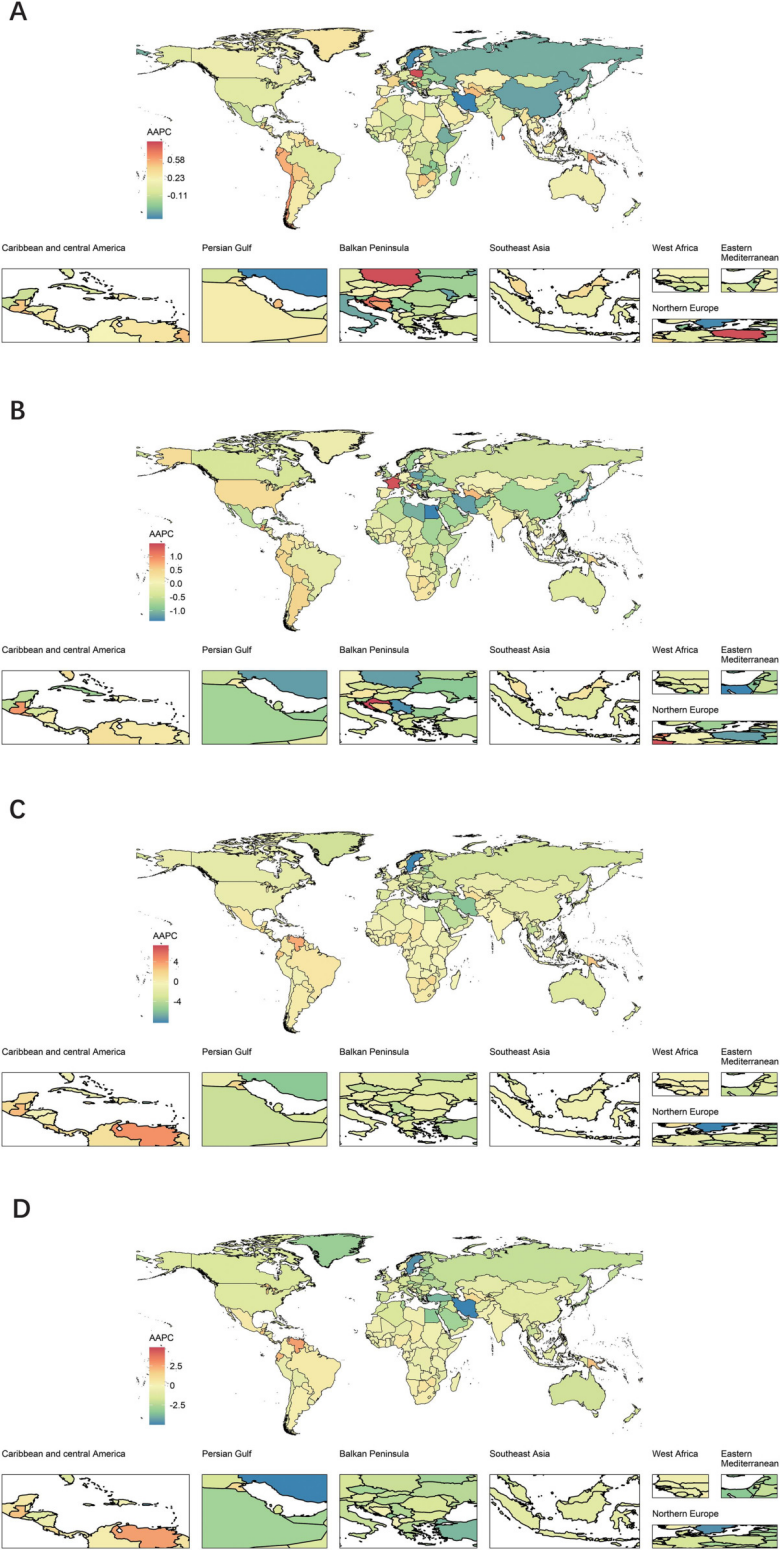
Global map of AAPC of ASR for prevalence **(A)**, incidence **(B)**, deaths **(C)** and DALYs **(D)** of urogenital congenital anomalies for both sexes by 204 countries and territories in 2021.

From the perspective of ASRs, 51 countries consistently demonstrated declines across different parameters. For example, Sweden showed a 90% reduction in mortality, the Islamic Republic of Iran experienced a 79% decline in DALYs and a 16% reduction in prevalence, while the Republic of Serbia reported a 33.9% decrease in incidence. In contrast, 37 countries consistently displayed increases in different parameters. Tokelau recorded a 600% rise in mortality, Niue experienced a 54% increase in DALYs and a 33.4% increase in prevalence, while American Samoa saw a 43.3% rise in incidence (Supplementary Tables S1–S4; Supplementary Figures S1–S8). Moreover, the AAPC of incidence, prevalence, mortality, and DALYs for 204 countries and territories from1990 to 2021 provided deeper insights (Figures 5A–D).

### Decomposition analysis of change in UCAs

**Figure 6:**
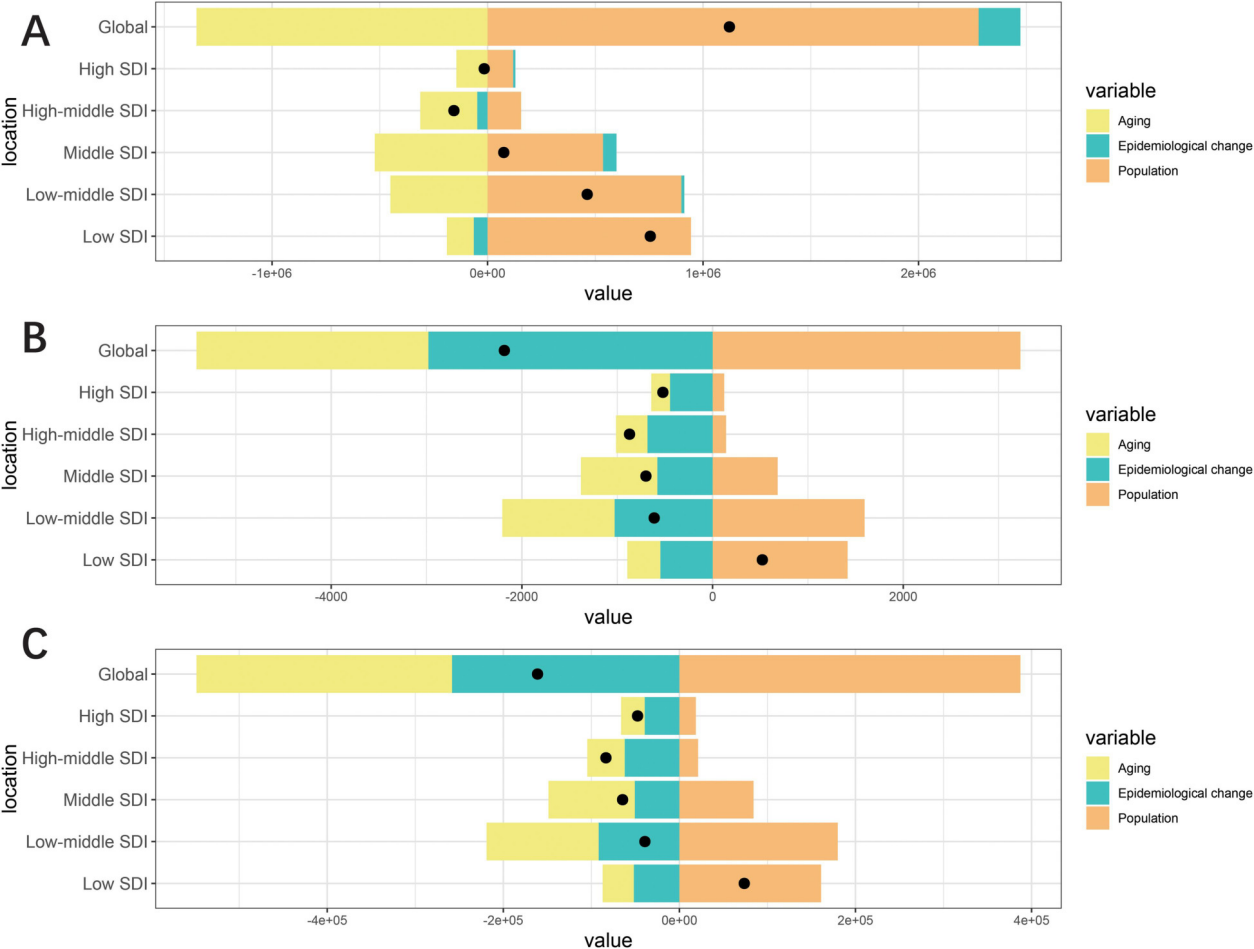
Change in prevalence **(A)**, deaths **(B)** and DALYs **(C)** of urogenital congenital anomalies decomposed by three population-level determinants: aging, population and epidemiological change at the global level and various regions. The black dots indicate the total value of change attributable to all three components.

The decomposition analysis examined changes in prevalence, deaths, and DALYs at the global level and across five SDI strata, attributing these changes to three population-level determinants: aging, population growth, and epidemiological change. Globally, the change in prevalence was primarily driven by population (203.03%), followed by epidemiological change (17.28%), while aging contributed negatively (−120.32%). Changes in deaths were largely attributable to population (−147.82%), followed by epidemiological change (136.47%) and aging (111.35%). Similarly, the change in DALYs was influenced primarily by population (−240.38%), followed by epidemiological change (180.14%) and aging (160.24%). The contributions of these three determinants to changes in the UCAs burden varied significantly across different SDI regions. Population was the main driver of increased disease burden in most SDI regions, whereas aging was identified as the key factor reducing disease burden in these regions (Figures 6A–C).

### Correlation of ASR with SDI

**Figure 7:**
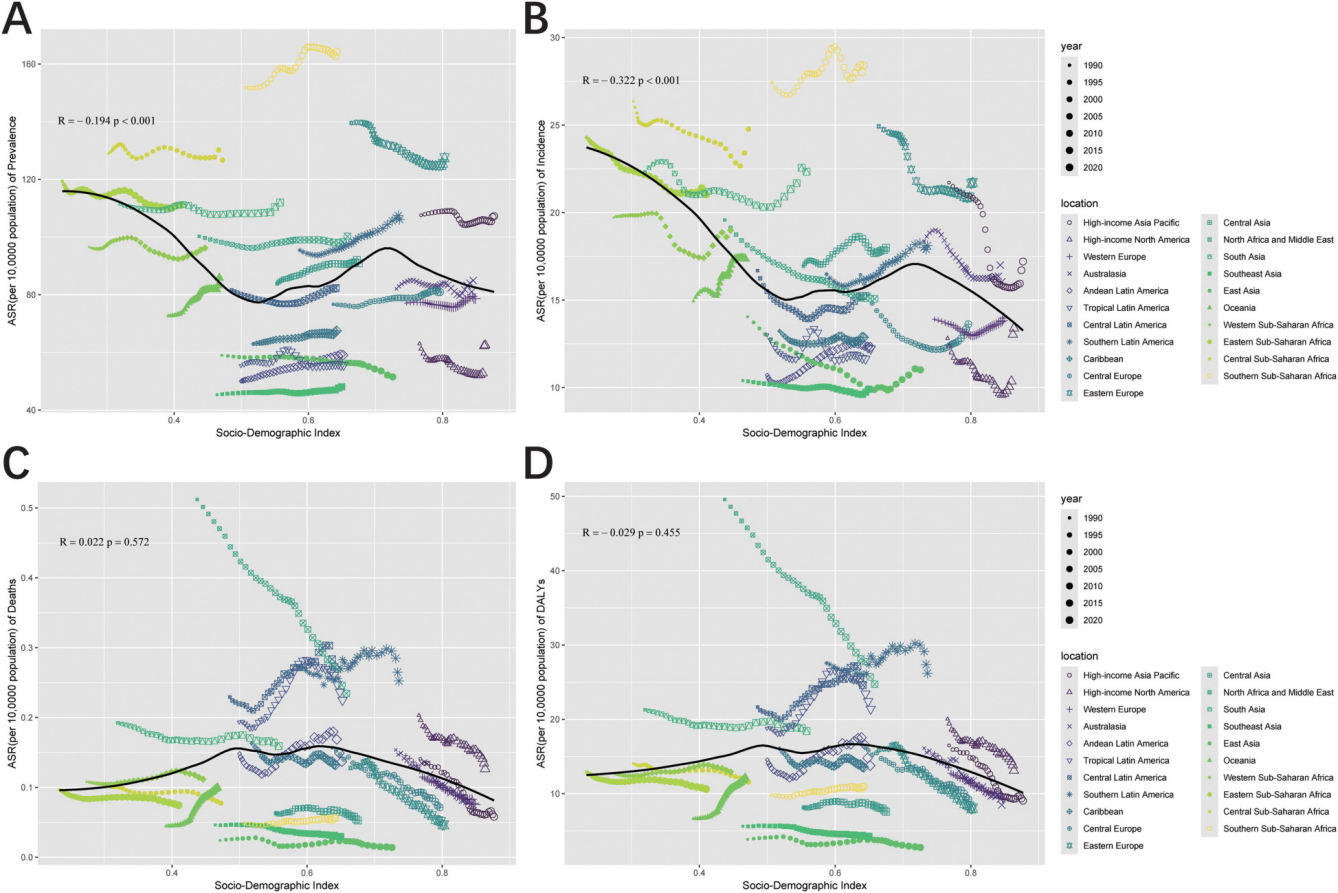
Pearson correlation analysis between the SDI and ASR of prevalence **(A)**, incidence **(B)**, deaths **(C)**, and DALYs **(D)** for urogenital congenital anomalies across 21 regional levels from 1990 to 2021 (The cases of prevalence, incidence, deaths, and DALYs from 21 regions from 1990 to 2021 are represented by different shapes. The size of the shapes increased with the year of prevalence, incidence, deaths, and DALYs.).

**Figure 8:**
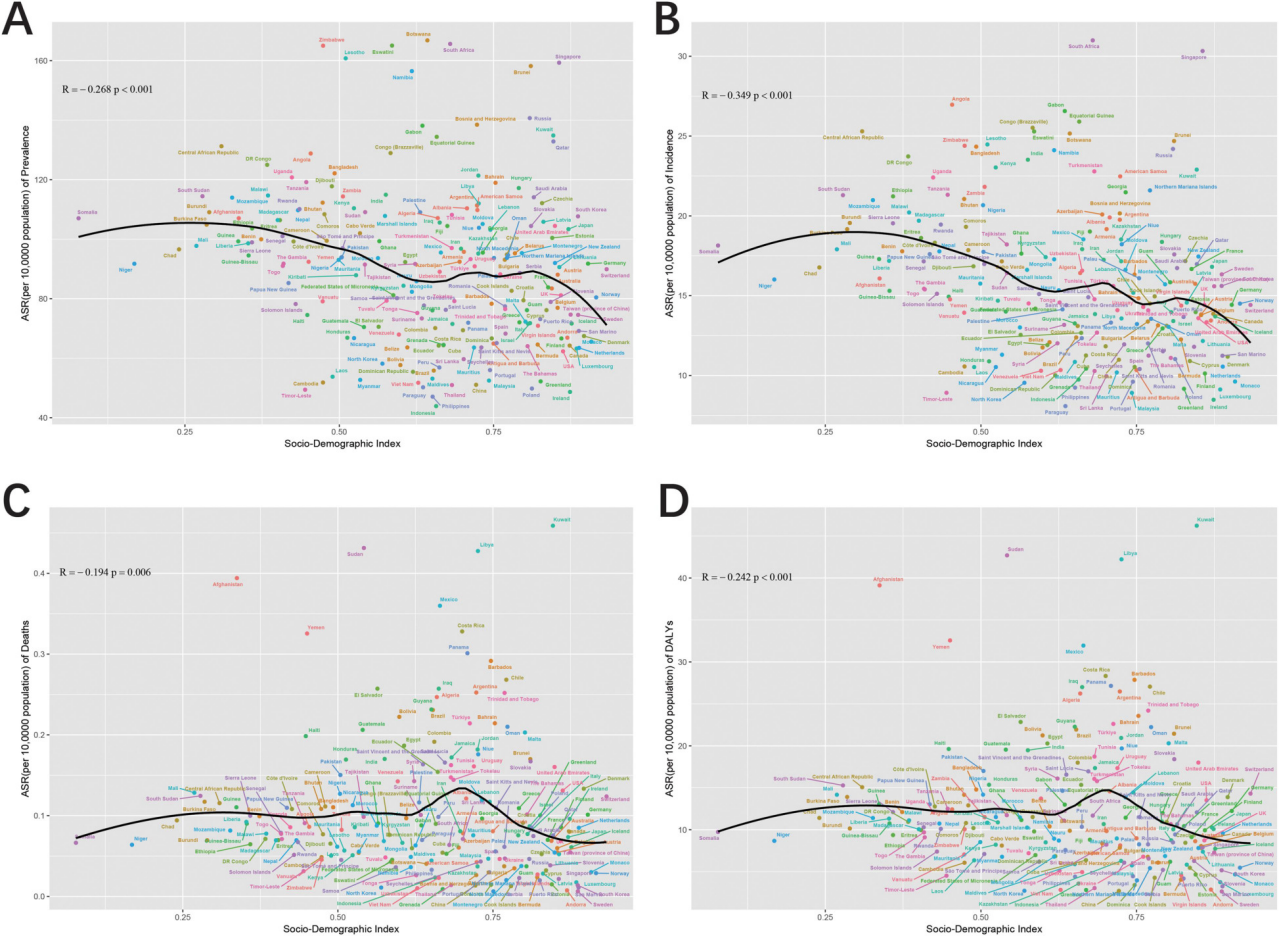
Pearson correlation analysis between the SDI and ASR of prevalence **(A)**, incidence **(B)**, deaths **(C)**, and DALYs **(D)** for urogenital congenital anomalies at the country and territorial levels in 2021 (The cases of prevalence, incidence, deaths, and DALYs from 204 countries and territories in 2021 are represented by different color.

By investigating UCAs metrics across 21 regions, a slight correlation with the SDI was observed. Mortality and DALYs ASRs showed no significant correlation with SDI, with slightly positive (*R* = 0.022, *P* = 0.572) and weak negative correlations (*R* = −0.029, *P* = 0.455), respectively, both failing to reach statistical significance (Figures 7C,D). In contrast, a strong negative correlation was observed between incidence ASR and SDI (*R* = −0.322, *P* < 0.001) (Figure 7A). This trend persisted for prevalence ASR, which also demonstrated a negative correlation with SDI (*R* = −0.194, *P* < 0.001) (Figure 7B). A broader analysis of 204 countries and territories revealed different results for mortality and DALYs (Figures 8C,D). Both were negatively correlated with SDI (Mortality: *R*R = −0.194, *P* = 0.006; DALYs: *R* = −0.242, *P* < 0.001). Similarly, incidence and prevalence ASRs also exhibited negative correlations with SDI (Incidence: *R* = −0.349, *P* < 0.001; Prevalence: *R* = −0.268, *P* < 0.001) (Figures 8A,B).

### Frontier analysis

In the comprehensive frontier analysis of SDI and ASRs for congenital UCAs across 204 countries and territories from 1990 to 2021, varying trends were observed. For prevalence, ASRs generally showed a declining trend as SDI values increased from 0.0 to 1.0. Similarly, incidence, mortality, and DALYs for UCAs also decreased with rising SDI values, indicating that as societies develop, the burden of UCAs tends to lessen.

**Figure 9:**
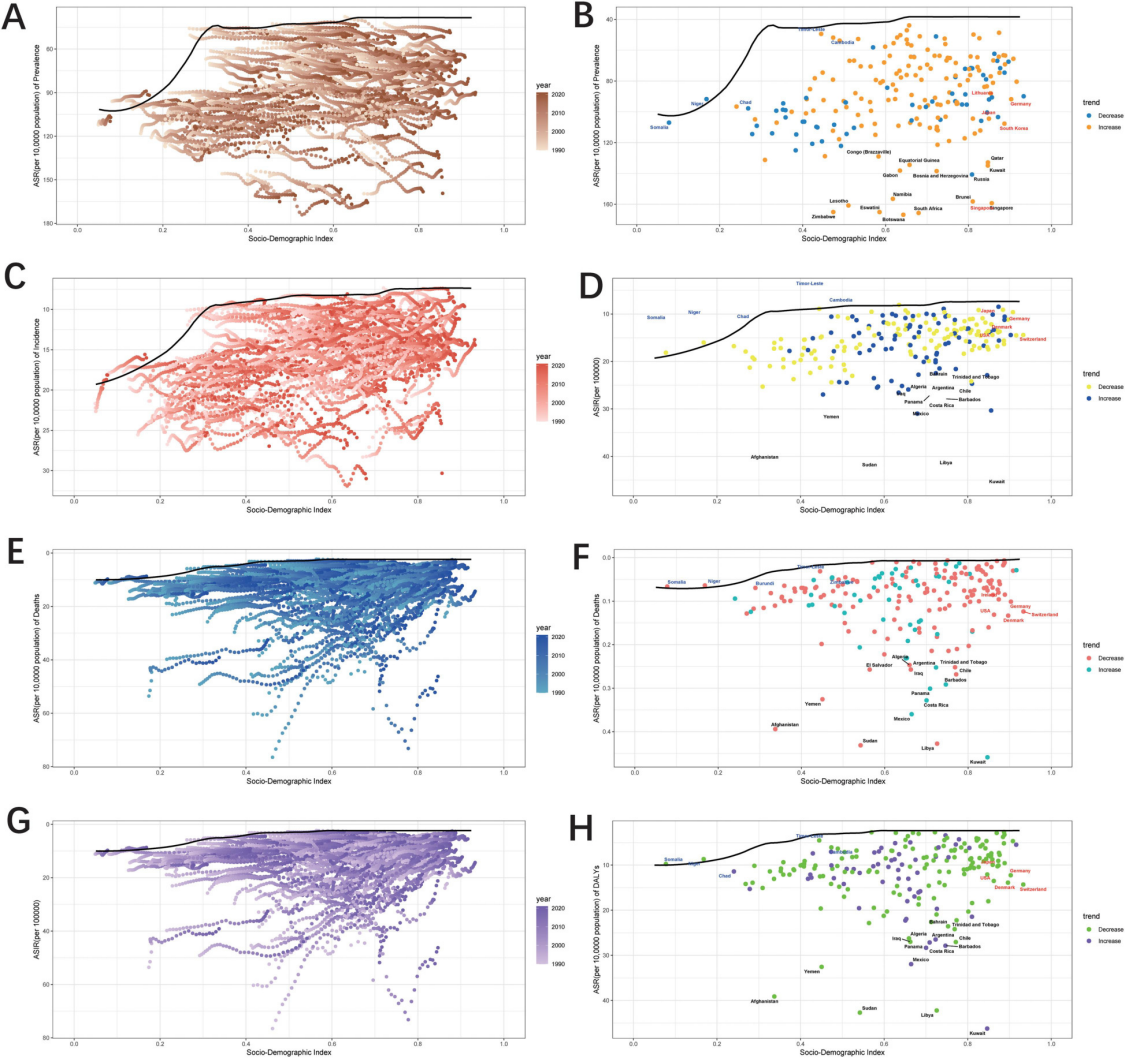
Frontier analysis, represented by the solid black lines, explores the relationship between Socio-Demographic Index (SDI) and Age-Standardized Rate (ASR) for prevalence **(A,B)**, incidence **(C,D)**, deaths **(E,F)**, and DALYs **(G,H)** in the context of urogenital congenital anomalies. The color gradient in graphs **A,C,E**, and G illustrates the progression of years, ranging from light shades representing 1990 to the darkest shades denoting 2021. In graphs **B,D,F**, and **H** each dot signifies a specific country or territory for the year 2021, with the top 15 countries displaying the most significant deviation from the frontier labeled in black. Countries with low SDI and minimal deviation from the frontier are highlighted in blue, while those with high SDI and notable deviation for their developmental level are emphasized in red. The direction of change from 1990 to 2021 in ASR is indicated by the color of the dots: decrease dots represents a decrease, while increase dots signifies an increase.

Focusing on the frontier analysis results since 2021, clear distinctions between countries and territories became evident. For prevalence, 15 countries, including Botswana, Zimbabwe, and South Africa, among others, exhibited significantly higher rates, far from the frontier (Figures 9A,B). In contrast, countries like Somalia, Niger, Chad, and Timor-Leste were closer to the frontier, suggesting optimal outcomes relative to their SDI levels. In mortality analysis, countries such as Afghanistan, Yemen, and Sudan showed substantial gaps from the frontier. Interestingly, some high-SDI countries, including Switzerland, Germany, and Denmark, demonstrated relatively high effective differences at their stage of development (Figures 9E,F). Finally, for incidence and DALYs, countries like Somalia, Niger, Chad, and Timor-Leste displayed rates closer to the expected benchmarks set by the frontier, indicating better alignment with expectations based on their SDI levels (Figures 9C,D,G,H).

### Cross-country health inequality analysis

**Figure 10:**
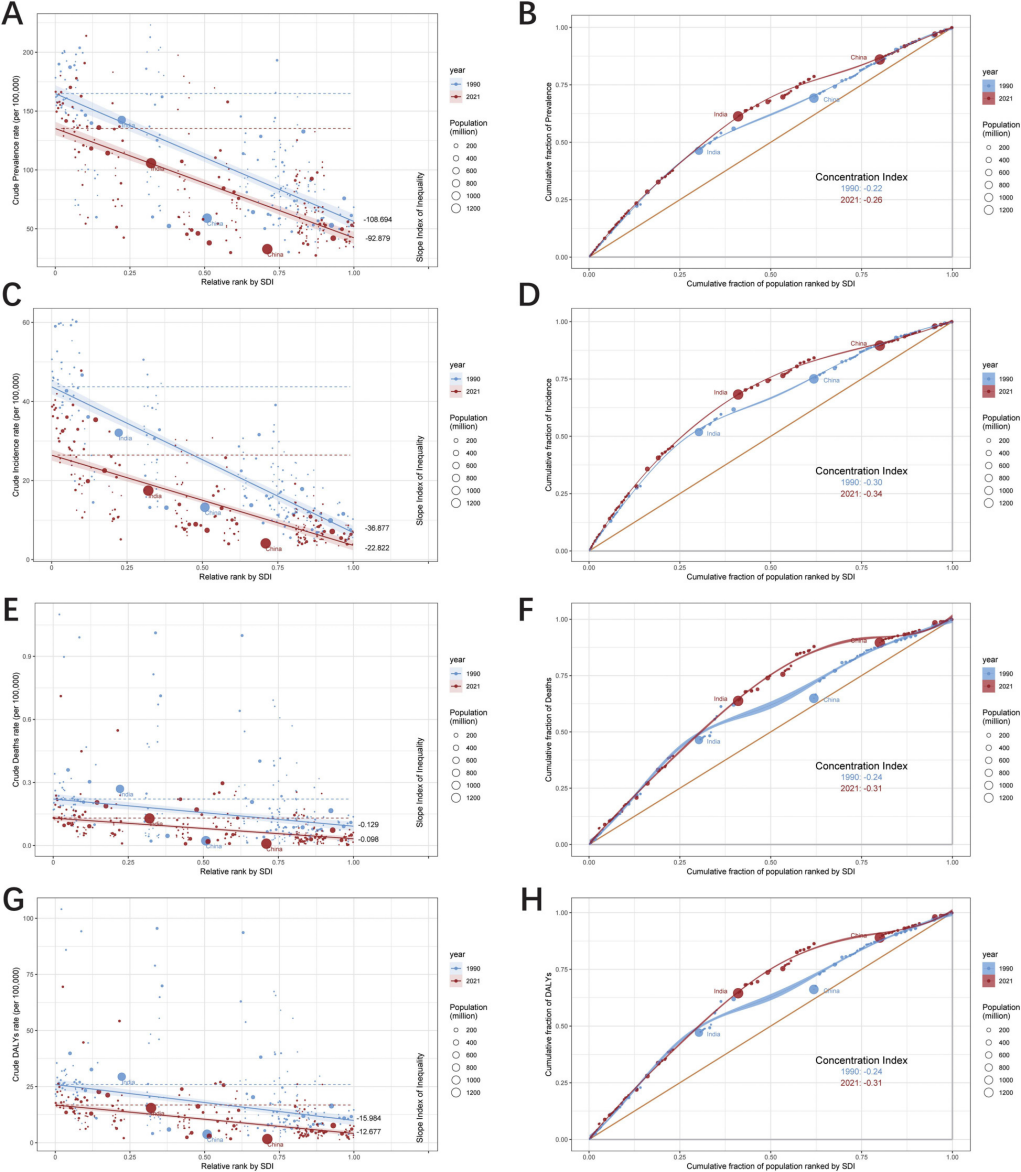
Health inequality regression curves and concentration curves for the prevalence **(A,B)**, incidence **(C,D)**, deaths **(E,F)** and DALYs **(G,H)** of urogenital congenital anomalies.

The results of the study revealed significant absolute and relative income inequality in the incidence, prevalence, mortality and DALYs burden of UCAs, with the burden heavily concentrated in poor regions. Comparing data from 1990 to 2021, the degree of health inequality between high-income and low-income countries has decreased over time. The concentration index for prevalence shifted from −0.22 in 1990 to −0.26 in 2021, while the slope index of inequality between low- and high-SDI regions decreased from −108.694 in 1990 to −92.879 in 2021 (Figures 10A,B). Similarly, the concentration index for incidence changed from −0.30 in 1990 to −0.34 in 2021, with the slope index of inequality reducing from −36.877 in 1990 to −22.822 in 2021 (Figures 10C,D). For mortality, the concentration index ranged from −0.24 in 1990 to −0.31 in 2021. However, the slope index of inequality for mortality did not show a significant reduction by 2021 (Figures 10E,F). Regarding DALYs, the concentration index shifted from −0.24 in 1990 to −0.31 in 2021, with the slope index of inequality decreasing from −15.984 in 1990 to −12.677 in 2021 (Figures 10G,H). These findings collectively indicate a reduction in the unequal burden of UCAs over time.

## Discussion

From 1990 to 2021, the global prevalence of UCAs cases increased by 21% to 6.34 million, with ASRs rising by 3.69%. In contrast, incidence, deaths, and DALYs experienced declines in both absolute numbers and ASRs. Females exhibited higher rates of prevalence and incidence, whereas males had higher rates of mortality and DALYs. In 2021, Southern Sub-Saharan Africa recorded the highest prevalence, with significant regional disparities observed. High SDI levels were associated with better UCAs outcomes. Aging and population contributed more significantly to changes in prevalence. The frontier analysis of 204 countries and territories from 1990 to 2021 revealed a general decline in UCAs prevalence as SDI levels increased. Countries such as Botswana and Zimbabwe reported higher prevalence rates, while Somalia and Niger performed better relative to their SDI levels. Interestingly, high-SDI countries like Singapore and Kuwait showed elevated prevalence rates. Additionally, the extent of health inequality across high-income and low-income decreased over the study period.

From 1990 to 2021, the global UCAs cases increased by 21%, while the incidence decreased by 6.25%. Simultaneously, mortality decreased by 23% and DALYs fell by 15%, collectively revealing a complex epidemiological profile for UCAs. This contrast suggests that, despite a declining incidence, the total number of UCAs cases has risen. Antenatal care and interventions likely contributed to reduced incidence (16, 17), whereas advancements in minimally invasive surgeries, including laparoscopic and robot-assisted procedure may lowered mortality and DALYs, extending patient survival and thereby expanding the prevalent patient population (18, 19). These insights provide a nuanced narrative of UCAs management: despite an overall rise in cases, there are encouraging signs of progress, highlighting opportunities for targeted healthcare strategies and further research.

Between 1990 and 2021, males consistently showed higher ASRs for mortality and DALYs, while females exhibited higher rates of incidence and prevalence, potentially due to anatomical differences and differences in the spectrum of UCAs (20). The observed divergence may be partially explained by the renoprotective effects of estrogen, which has been shown to mitigate progression of renal pathologies (21). Additionally, severe malformations more prevalent in males, such as posterior urethral valves associated with early-onset renal dysfunction, could contribute to this pattern (22, 23). Both sexes experienced a decline in incidence from 1990 to 2016, possibly linked to more effective medical interventions and heightened health awareness. However, the upward trend in incidence between 2016 and 2021 warrants further investigation. Chen et al. identified significant positive correlations between UCAs burden and several air pollutants, notably sulfur dioxide (SO₂), ammonia, and nitrogen oxides, which may underlie this recent epidemiological shift. Their findings recommend minimizing maternal exposure to ammonia and SO₂ and emphasize prioritizing air pollution control in public health monitoring policies (24). While global health strategies have successfully reduced the disease burden for both genders, females experienced greater declines in mortality and DALYs, suggesting that they may have benefited more from these medical advancements. Nonetheless, the rise in prevalence underscores the influence of population growth and incidence fluctuations, emphasizing the need for continued gender-specific research to optimize prevention and treatment strategies.

Over the past three decades, UCAs trends across regions and countries have highlighted the complex interplay of sociodemographic, economic, and healthcare determinants. The high prevalence in Southern Sub-Saharan Africa, compared to the much lower prevalence in Southeast Asia, underscores regional disparities driven by factors such as genetic predisposition, healthcare accessibility, and environmental influences (25–27). The substantial decline in East Asia may reflect improvements in healthcare systems, advancements in UCAs management strategies, and the effects of aging (28). China, as a major East Asian economy, demonstrated this progress through its 2016 Healthy China 2030. The nationally implemented measures, including universal preconception screening, subsidized genetic counseling and diagnostic testing for high-risk pregnancies, and expanded newborn screening coverage with broader disease panels, collectively achieved effective reduction in child health burdens attributable to congenital birth defects (29). Conversely, the rise in Oceania is likely attributable to population growth and improved screening capabilities (30). Eastern Europe's sharp decline in mortality points to the effectiveness of its healthcare programs and the accessibility of timely treatment, whereas the surge in mortality in Oceania suggests potential gaps in healthcare services or emerging risk factors requiring further investigation.

At the national level, island nations in Oceania have seen increases in UCAs measures, potentially driven by factors such as child poverty, limited healthcare access, inadequate funding for pediatric research, and poor living conditions (31, 32). In contrast, countries like the Islamic Republic of Iran and the Kingdom of Sweden experienced significant reductions in mortality and DALYs, reflecting improvements in healthcare accessibility and sanitation (33, 34). The varying patterns observed across regions and countries underscore the importance of tailored health strategies that address the unique challenges and strengths of each region.

The relationship between SDI and UCAs outcomes showed a significant negative correlation, emphasizing the favorable impact of higher SDI on reducing incidence, prevalence, mortality, and DALYs for UCAs. This correlation likely reflects the benefits of stronger healthcare systems, reduced exposure to risk factors, and greater access to medical services in higher-SDI regions (35). The data affirm the critical role of sociodemographic progress in improving UCAs outcomes, highlighting the importance of prioritizing SDI improvements as a central goal in health policy (36).

The frontier analysis of SDI and ASRs for UCAs from 1990 to 2021 provides key insights into global UCAs trends. The overall narrative illustrates a clear trajectory: as sociodemographic indicators improve, the burden of UCAs—whether measured by prevalence, mortality, or DALYs—tends to diminish. However, a closer examination of 2021 data reveals subtle differences between countries. Notably, countries like Singapore, Botswana, and Zimbabwe, despite their differing sociodemographic contexts, experienced declining prevalence over 30 years but remain far from the optimal frontier. In contrast, countries like Somalia, Niger, and Chad—often facing significant sociopolitical challenges—appear to perform better than expected relative to their SDI levels, coming closer to the frontier benchmarks. This divergence highlights the multifaceted nature of healthcare outcomes, indicating that while SDI is a critical determinant, other factors—whether environmental, genetic, or healthcare system-related—also play key roles in shaping UCAs trends.

Our study has some limitations. While we have endeavored to provide a comprehensive analysis, data accuracy and consistency across regions may vary, with particular challenges arising from data gaps in low-resource settings, introducing potential bias or inaccuracies. Additionally, our historical scope, incorporating data from 1990, may be influenced by evolving diagnostic criteria and medical technologies over time. Furthermore, the modeling assumptions used in data synthesis and estimation carry inherent potential biases, and the lack of primary data precludes direct clinical validation of the estimates. Thus, while our findings offer valuable insights into global UCAs trends, they should be interpreted with caution, acknowledging the subtle complexities and potential biases inherent in the broader context of our research.

## Conclusion

Our study underscores significant patterns associating UCAs with socio-demographic factors, providing valuable insights for shaping health policies and practices. The findings reveal that regions with higher socio-demographic indices experience fewer UCAs-related mortalities and improved outcomes, although inequities remain evident. Given the persistent global burden of UCAs, the importance of ongoing surveillance and adaptable healthcare strategies is clear. Achieving meaningful reductions in the impact of UCAs will necessitate sustained research efforts and proactive healthcare interventions.

## Data Availability

Publicly available datasets were analyzed in this study. This data can be found here: http://ghdx.healthdata.org/gbd-results-tool.
