# Supplementary material for: Burden of urogenital congenital anomalies: findings from the global burden of disease study 2021
Source: Front Pediatr. 2025 Sep 25;13:1584280. doi: 10.3389/fped.2025.1584280 (PMC12507638; doi:10.3389/fped.2025.1584280)
Supplement: Supplementary file 1 [file Supplementaryfile1.docx]

**Table S1** **Numbers** **and ASRs per 100,000 Cases of prevalence of urogenital congenital anomalies in 1990 and 2021, along with the relative changes and AAPC in ASRs per 100,000 Cases from 1990-2021, Categorized by 204 countries and territories**

| Characteristic | Number in 1990  （95% CI） | Age-standardized  Rate in 1990  （95% CI） | Number in 2021  （95% CI） | Age-standardized  Rate in 2021  （95% CI） | Relative Change of numbers from 1990 to 2021（%） | Relative Change of age-standardized  rate from 1990 to 2021（%） | AAPC  (Age-standardized  Rate, 95% CI) | *P* value |
| --- | --- | --- | --- | --- | --- | --- | --- | --- |
| American Samoa | 56.3692 (43.3226,72.4895) | 85.6027 (65.4722,110.1788) | 49.8764 (38.4427,64.145) | 109.7885 (85.3476,140.6234) | -0.11518 | 0.282535 | 0.80 (0.74 to 0.87) | <0.001 |
| Antigua and Barbuda | 35.138 (27.9901,42.9186) | 55.473 (44.0721,67.7251) | 43.4504 (34.8584,53.2909) | 64.9308 (51.8812,78.9186) | 0.236564 | 0.170494 | 0.46 (0.33 to 0.59) | <0.001 |
| Arab Republic of Egypt | 69296.0473 (52082.6888,87890.0124) | 92.2992 (69.5258,116.9952) | 113303.431 (87024.3696,146302.8822) | 92.3193 (70.672,118.5575) | 0.635063 | 0.000218 | -0.02 (-0.04 to 0.01) | 0.167 |
| Argentine Republic | 35271.5483 (28341.5897,43663.6378) | 103.5437 (83.3215,127.8364) | 41445.6476 (32766.8338,51670.5833) | 111.3428 (88.5152,137.7641) | 0.175045 | 0.075322 | 0.26 (0.22 to 0.30) | <0.001 |
| Australia | 11098.2988 (8967.1976,13768.9031) | 79.221 (64.0896,98.5191) | 14799.7595 (11935.7189,17954.0932) | 83.4417 (67.5638,101.9403) | 0.333516 | 0.053278 | 0.12 (-0.09 to 0.34) | 0.252 |
| Barbados | 157.8762 (126.6872,192.831) | 70.0806 (56.1968,85.7137) | 143.2094 (114.883,175.3131) | 78.3134 (62.4878,95.6165) | -0.0929 | 0.117476 | 0.37 (0.24 to 0.49) | <0.001 |
| Belize | 160.6372 (129.0849,197.0423) | 59.8221 (48.2674,72.9433) | 274.8342 (216.905,336.9495) | 63.6324 (50.6002,78.2769) | 0.7109 | 0.063694 | 0.23 (0.16 to 0.30) | <0.001 |
| Bermuda | 29.4085 (23.767,35.564) | 61.4551 (49.2486,75.0611) | 21.4983 (17.2733,26.0796) | 64.5843 (51.1995,77.935) | -0.26898 | 0.050918 | 0.16 (0.14 to 0.19) | <0.001 |
| Bolivarian Republic of Venezuela | 14687.641 (11666.3403,17704.5319) | 62.2056 (49.6222,74.9922) | 15997.3019 (12912.5778,19194.7389) | 67.9563 (54.5961,81.6289) | 0.089168 | 0.092447 | 0.29 (0.26 to 0.32) | <0.001 |
| Bosnia and Herzegovina | 4629.4491 (3621.3738,5951.593) | 115.0311 (90.2591,148.2243) | 2693.0672 (2102.5498,3436.6942) | 138.4693 (106.1123,176.8651) | -0.41827 | 0.203755 | 0.59 (0.47 to 0.71) | <0.001 |
| Brunei Darussalam | 420.221 (328.4805,531.5004) | 131.6103 (102.3728,165.8336) | 597.6174 (469.9572,755.4828) | 158.185 (123.0891,203.19) | 0.42215 | 0.20192 | 0.59 (0.55 to 0.63) | <0.001 |
| Burkina Faso | 15524.6381 (12123.3054,19315.2745) | 101.3396 (81.0324,125.6574) | 35931.5803 (28260.04,45094.3502) | 104.8772 (83.1432,131.6298) | 1.314487 | 0.034908 | 0.11 (0.03 to 0.18) | 0.006 |
| Canada | 13592.1017 (11209.2955,16063.2826) | 61.9692 (51.4271,73.3129) | 15246.8059 (12163.3638,18365.2913) | 64.2518 (51.476,77.1349) | 0.12174 | 0.036834 | 0.11 (-0.11 to 0.34) | 0.326 |
| Central African Republic | 5257.8635 (4051.865,6605.1857) | 125.9813 (98.0402,158.9787) | 9877.2177 (7741.1221,12535.4302) | 131.2397 (103.6605,166.4228) | 0.878561 | 0.04174 | 0.12 (0.10 to 0.14) | <0.001 |
| Commonwealth of Dominica | 48.2481 (37.7743,59.2019) | 57.1023 (44.643,69.9369) | 33.6116 (26.7279,40.5831) | 66.6547 (53.2262,81.1256) | -0.30336 | 0.167286 | 0.49 (0.43 to 0.55) | <0.001 |
| Commonwealth of the Bahamas | 167.0027 (131.203,204.8296) | 59.633 (47.009,72.9471) | 191.4826 (152.5296,232.2652) | 64.0362 (51.5439,78.4669) | 0.146584 | 0.073838 | 0.24 (0.22 to 0.27) | <0.001 |
| Cook Islands | 15.8415 (12.3815,20.3522) | 71.8892 (56.3123,91.5929) | 11.1701 (8.7546,14.608) | 82.6658 (64.2559,106.7596) | -0.29488 | 0.149906 | 0.46 (0.42 to 0.50) | <0.001 |
| Czech Republic | 8454.5113 (6727.1887,10564.8882) | 107.8888 (86.2911,133.9142) | 7241.2594 (5762.9804,8987.315) | 112.1128 (89.0585,138.7533) | -0.1435 | 0.039151 | 0.08 (-0.05 to 0.22) | 0.215 |
| Democratic People's Republic of Korea | 13547.3281 (10791.0095,16617.5061) | 61.1111 (48.4499,74.9119) | 11220.7353 (8882.9952,13945.6096) | 58.1759 (46.5312,72.3287) | -0.17174 | -0.04803 | -0.15 (-0.22 to -0.08) | <0.001 |
| Democratic Republic of Sao Tome and Principe | 172.4142 (135.2163,216.8383) | 96.684 (77.1889,120.978) | 247.2296 (195.692,308.7748) | 96.9572 (77.0691,120.7243) | 0.433928 | 0.002826 | 0.01 (-0.05 to 0.08) | 0.666 |
| Democratic Republic of the Congo | 77739.2504 (59929.2121,97839.8516) | 129.825 (100.3938,164.8095) | 153178.8541 (119916.9652,197621.6946) | 124.9894 (98.7551,160.1102) | 0.970418 | -0.03725 | -0.10 (-0.24 to 0.03) | 0.139 |
| Democratic Republic of Timor-Leste | 545.1488 (421.7688,681.1452) | 45.8044 (35.7006,57.5154) | 866.2517 (672.0677,1092.881) | 49.4123 (38.4547,62.286) | 0.589019 | 0.078768 | 0.21 (0.08 to 0.35) | 0.002 |
| Democratic Socialist Republic of Sri Lanka | 9163.2134 (6983.0179,12066.5717) | 48.8401 (37.3164,63.9392) | 10925.9718 (8385.9861,14010.1832) | 59.6834 (46.0828,76.1847) | 0.192373 | 0.222016 | 0.68 (0.57 to 0.79) | <0.001 |
| Dominican Republic | 4685.6841 (3695.9545,5808.5588) | 50.2127 (39.7433,62.3811) | 5931.8048 (4661.8199,7203.0567) | 55.4255 (43.6743,67.4099) | 0.265942 | 0.103814 | 0.31 (0.26 to 0.36) | <0.001 |
| Eastern Republic of Uruguay | 2429.5863 (1959.2401,3001.6572) | 84.9176 (68.2698,104.9147) | 2275.8931 (1829.5556,2779.688) | 90.8801 (72.3567,111.8498) | -0.06326 | 0.070215 | 0.23 (0.02 to 0.43) | 0.031 |
| Federal Democratic Republic of Ethiopia | 96052.638 (75476.402,120491.9964) | 118.1657 (93.7804,148.5983) | 154295.7726 (120837.487,193518.9461) | 103.6133 (81.8403,129.6557) | 0.606367 | -0.12315 | -0.41 (-0.49 to -0.33) | <0.001 |
| Federal Democratic Republic of Nepal | 31104.236 (23192.952,41332.9635) | 111.3604 (82.8421,147.2033) | 35859.0806 (26787.1208,47609.0993) | 109.728 (82.4025,145.2375) | 0.152868 | -0.01466 | -0.13 (-0.25 to -0.02) | 0.022 |
| Federal Republic of Germany | 49014.4167 (39230.196,59825.4701) | 90.5076 (72.7139,110.7392) | 45311.2856 (36114.0305,55929.5592) | 91.786 (73.3431,114.627) | -0.07555 | 0.014125 | 0.04 (-0.00 to 0.08) | 0.084 |
| Federal Republic of Nigeria | 131940.3566 (104803.9987,161910.9767) | 96.28 (77.1082,119.1178) | 314381.7911 (249177.6367,389093.9636) | 93.8458 (74.8789,116.0462) | 1.382757 | -0.02528 | -0.07 (-0.17 to 0.02) | 0.126 |
| Federal Republic of Somalia | 14158.2946 (10976.8552,17687.7616) | 112.1209 (88.2545,141.25) | 35955.6568 (28120.407,45075.166) | 107.0245 (84.6386,135.1757) | 1.539547 | -0.04545 | -0.11 (-0.30 to 0.08) | 0.25 |
| Federated States of Micronesia | 96.2367 (74.7059,123.3036) | 67.0022 (53.0334,85.1688) | 85.1586 (67.0275,106.7162) | 81.1715 (64.0624,101.6252) | -0.11511 | 0.211475 | 0.64 (0.55 to 0.72) | <0.001 |
| Federative Republic of Brazil | 95330.9673 (76756.9198,116066.0969) | 55.816 (44.9878,67.7056) | 101639.7445 (82116.5261,121964.1477) | 55.8471 (44.8828,67.4744) | 0.066178 | 0.000557 | 0.01 (-0.05 to 0.08) | 0.668 |
| French Republic | 32788.7115 (26815.1387,39961.9597) | 72.1566 (59.1612,86.7778) | 35831.6738 (29051.7572,43614.0405) | 83.8058 (68.4928,101.7519) | 0.092805 | 0.161443 | 0.42 (0.17 to 0.67) | <0.001 |
| Gabonese Republic | 1772.6145 (1357.4007,2268.7118) | 130.3321 (99.9727,165.4886) | 2929.1662 (2266.561,3727.4888) | 138.13 (107.526,174.7755) | 0.652455 | 0.059831 | 0.20 (0.09 to 0.30) | <0.001 |
| Georgia | 4105.2106 (3239.887,5044.6658) | 84.2142 (66.5018,103.7942) | 2499.2427 (2033.5545,3011.1659) | 95.4835 (77.2403,115.1531) | -0.3912 | 0.133817 | 0.35 (0.25 to 0.45) | <0.001 |
| Grand Duchy of Luxembourg | 157.4899 (122.9912,194.6541) | 58.0042 (45.4653,72.3718) | 266.4066 (211.6547,329.2934) | 63.2553 (50.7022,78.6164) | 0.691579 | 0.09053 | 0.29 (0.21 to 0.36) | <0.001 |
| Greenland | 26.8421 (21.3626,32.7039) | 47.5506 (37.9307,58.6098) | 23.3556 (18.5464,28.2021) | 52.2127 (41.5852,63.2884) | -0.12989 | 0.098045 | 0.30 (0.28 to 0.33) | <0.001 |
| Grenada | 60.7639 (48.0336,74.9469) | 55.6239 (43.995,68.4638) | 54.0533 (43.8545,65.8206) | 64.4776 (52.4037,78.3326) | -0.11044 | 0.159171 | 0.50 (0.44 to 0.56) | <0.001 |
| Guam | 98.2168 (77.4924,123.6542) | 62.938 (49.6539,79.4585) | 101.5263 (81.1956,127.1155) | 76.1471 (60.1505,95.365) | 0.033696 | 0.209875 | 0.64 (0.58 to 0.70) | <0.001 |
| Hashemite Kingdom of Jordan | 6191.4289 (4794.2041,7881.3291) | 115.8891 (90.6072,147.5624) | 15448.9474 (12309.87,19291.7646) | 121.3793 (96.6352,151.3536) | 1.495215 | 0.047375 | 0.15 (0.10 to 0.19) | <0.001 |
| Hellenic Republic | 5376.7541 (4233.1641,6688.8412) | 73.8152 (58.0084,90.8719) | 4024.7224 (3216.1707,4913.9929) | 72.0677 (57.5213,87.7281) | -0.25146 | -0.02367 | -0.08 (-0.10 to -0.06) | <0.001 |
| Hungary | 8507.1365 (6676.9585,10644.9444) | 112.3906 (87.8267,141.0937) | 6513.473 (5196.6438,8027.9838) | 117.1831 (92.3548,145.4503) | -0.23435 | 0.042641 | 0.16 (0.08 to 0.24) | <0.001 |
| Independent State of Papua New Guinea | 4081.9607 (3189.9181,5120.1268) | 70.9012 (55.4872,88.8676) | 11584.6903 (9008.6417,14775.4948) | 85.2594 (66.5882,109.4079) | 1.838021 | 0.20251 | 0.58 (0.56 to 0.61) | <0.001 |
| Independent State of Samoa | 170.0516 (132.0388,219.3275) | 73.2 (57.6296,93.3421) | 211.7931 (163.8546,272.4418) | 80.1751 (62.2622,103.0127) | 0.245464 | 0.095288 | 0.34 (0.23 to 0.44) | <0.001 |
| Ireland | 1401.9588 (1128.9544,1729.247) | 42.2481 (34.388,51.5363) | 1726.5164 (1361.1485,2143.4281) | 48.6735 (38.8134,60.1997) | 0.231503 | 0.152087 | 0.44 (0.34 to 0.53) | <0.001 |
| Islamic Republic of Afghanistan | 15208.3443 (11822.8384,19523.0154) | 106.0643 (82.9152,133.6565) | 48874.4438 (38802.3893,61802.3984) | 107.0431 (85.3816,134.6175) | 2.21366 | 0.009228 | 0.04 (0.00 to 0.07) | 0.029 |
| Islamic Republic of Iran | 92579.2021 (72310.2034,118005.9407) | 115.3452 (91.5905,146.1588) | 69415.0358 (55241.8093,87271.8853) | 96.7964 (77.4032,121.0614) | -0.25021 | -0.16081 | -0.56 (-0.59 to -0.53) | <0.001 |
| Islamic Republic of Mauritania | 2667.4165 (2097.6926,3262.9566) | 87.3691 (68.9757,106.956) | 5522.3387 (4305.1997,6975.0303) | 93.0406 (72.7957,117.3063) | 1.070295 | 0.064914 | 0.20 (0.13 to 0.27) | <0.001 |
| Islamic Republic of Pakistan | 155377.0747 (117723.7938,201358.5901) | 96.0888 (73.1571,125.1428) | 269309 (206940.97,348682.9213) | 93.9412 (72.533,121.7054) | 0.733261 | -0.02235 | -0.10 (-0.18 to -0.01) | 0.023 |
| Jamaica | 1810.5073 (1418.251,2224.496) | 64.7335 (51.0302,79.0982) | 1593.3123 (1277.2764,1958.3497) | 71.5133 (57.6943,88.4619) | -0.11996 | 0.104734 | 0.33 (0.29 to 0.38) | <0.001 |
| Japan | 95430.046 (75227.9843,117982.9386) | 113.0809 (89.742,139.4529) | 63460.3536 (51397.4724,77112.9193) | 103.3762 (83.7128,125.8011) | -0.33501 | -0.08582 | -0.29 (-0.35 to -0.23) | <0.001 |
| Kingdom of Bahrain | 617.1185 (485.4367,792.4428) | 104.1108 (81.6629,134.8058) | 1517.462 (1197.441,1917.121) | 118.9501 (94.1979,150.939) | 1.458948 | 0.142534 | 0.44 (0.38 to 0.50) | <0.001 |
| Kingdom of Belgium | 4993.9413 (4119.6988,6095.5617) | 69.4789 (57.7122,84.0373) | 5574.3062 (4533.6688,6820.1304) | 76.935 (62.7458,94.3198) | 0.116214 | 0.107315 | 0.33 (0.27 to 0.39) | <0.001 |
| Kingdom of Bhutan | 951.8479 (734.9016,1217.9863) | 108.5524 (83.9007,138.3171) | 789.6129 (606.6931,1031.5011) | 112.2766 (86.2284,147.2183) | -0.17044 | 0.034308 | 0.11 (0.07 to 0.15) | <0.001 |
| Kingdom of Cambodia | 7211.9531 (5574.6373,9099.5683) | 46.3197 (35.2317,58.1581) | 9265.3746 (7149.3089,11905.4195) | 51.8358 (40.1178,66.4462) | 0.284725 | 0.119088 | 0.38 (0.24 to 0.51) | <0.001 |
| Kingdom of Denmark | 2669.497 (2160.4952,3271.5864) | 74.6337 (59.7727,92.2765) | 2521.2714 (2028.7446,3067.3977) | 67.4545 (54.0207,81.754) | -0.05553 | -0.09619 | -0.31 (-0.38 to -0.23) | <0.001 |
| Kingdom of Eswatini | 1799.6973 (1383.2584,2295.4343) | 150.172 (117.2712,190.3723) | 2281.6031 (1795.8532,2870.9644) | 165.0583 (130.4845,207.5814) | 0.26777 | 0.099128 | 0.31 (0.24 to 0.38) | <0.001 |
| Kingdom of Lesotho | 3067.9054 (2391.2295,3866.6293) | 145.8559 (114.3618,184.6917) | 3431.7506 (2655.9275,4432.1644) | 160.7812 (125.2406,205.9602) | 0.118597 | 0.102329 | 0.31 (0.28 to 0.34) | <0.001 |
| Kingdom of Morocco | 27110.8346 (21230.1137,34343.5219) | 83.1487 (64.7136,105.0715) | 32966.1549 (26012.4582,41913.7904) | 93.6311 (73.7931,119.789) | 0.215977 | 0.126068 | 0.39 (0.34 to 0.43) | <0.001 |
| Kingdom of Norway | 2421.551 (1883.8458,3092.3196) | 77.3102 (60.3499,98.2622) | 2825.2713 (2240.7915,3507.4985) | 80.4404 (63.782,99.5744) | 0.16672 | 0.040489 | 0.13 (0.11 to 0.14) | <0.001 |
| Kingdom of Saudi Arabia | 22870.657 (17662.7602,29105.1717) | 105.0181 (81.6269,132.6385) | 36891.2238 (28918.5056,47360.2901) | 114.0851 (89.0731,146.181) | 0.613037 | 0.086337 | 0.24 (0.16 to 0.33) | <0.001 |
| Kingdom of Spain | 18444.199 (14580.7215,22998.262) | 64.5463 (51.8161,79.1281) | 17115.7064 (13363.2406,21603.5518) | 68.2577 (54.4083,84.3223) | -0.07203 | 0.0575 | 0.22 (-0.22 to 0.66) | 0.329 |
| Kingdom of Sweden | 5346.2976 (4290.0461,6490.8539) | 88.5819 (70.4594,108.4654) | 4940.8518 (3965.5658,5996.8305) | 74.56 (59.3272,90.9406) | -0.07584 | -0.15829 | -0.54 (-0.57 to -0.50) | <0.001 |
| Kingdom of Thailand | 28494.8117 (21504.3934,36054.8249) | 48.8179 (36.7573,61.6182) | 19860.2129 (15323.8146,25276.6337) | 50.9772 (39.6979,65.2704) | -0.30302 | 0.044232 | 0.17 (0.11 to 0.24) | <0.001 |
| Kingdom of the Netherlands | 6722.946 (5430.4105,8205.2092) | 59.0735 (48.633,71.2637) | 6748.243 (5381.6308,8362.1774) | 63.467 (51.275,78.2336) | 0.003763 | 0.074373 | 0.27 (0.22 to 0.31) | <0.001 |
| Kingdom of Tonga | 92.5725 (72.385,119.1462) | 68.1392 (53.9574,87.5543) | 97.5105 (76.1947,124.6967) | 75.2058 (58.7411,95.5973) | 0.053342 | 0.103708 | 0.33 (0.24 to 0.42) | <0.001 |
| Kyrgyz Republic | 4862.0521 (3787.4522,6010.0435) | 84.9694 (66.2397,104.8943) | 6462.7969 (5068.5732,8015.7379) | 84.3534 (66.5079,104.1272) | 0.329232 | -0.00725 | -0.04 (-0.11 to 0.03) | 0.292 |
| Lao People's Democratic Republic | 3113.1315 (2424.4436,3915.7927) | 50.3897 (39.2476,63.5603) | 4392.2777 (3342.6071,5711.0913) | 53.7805 (40.9083,69.8041) | 0.410887 | 0.067292 | 0.17 (0.05 to 0.28) | 0.007 |
| Lebanese Republic | 3456.6242 (2661.6039,4395.8903) | 96.9843 (74.9089,123.3566) | 4953.0363 (3815.9716,6332.1224) | 103.177 (79.1998,131.1179) | 0.432911 | 0.063853 | 0.20 (0.14 to 0.25) | <0.001 |
| Malaysia | 10362.2952 (7980.9239,13133.6869) | 46.438 (35.9046,59.2733) | 14891.4379 (11409.3812,19402.3624) | 52.4829 (40.6268,66.8789) | 0.437079 | 0.130171 | 0.36 (0.23 to 0.49) | <0.001 |
| Mongolia | 2444.588 (1912.8939,3085.4276) | 80.7459 (63.602,101.0276) | 3022.7814 (2390.2962,3725.6278) | 82.3174 (65.0196,101.4826) | 0.23652 | 0.019462 | 0.02 (-0.04 to 0.08) | 0.47 |
| Montenegro | 616.3128 (469.4567,781.2539) | 108.9112 (82.7513,138.2153) | 404.178 (317.2373,517.6858) | 95.3257 (75.2885,122.8331) | -0.3442 | -0.12474 | -0.41 (-0.44 to -0.38) | <0.001 |
| New Zealand | 2849.5539 (2263.041,3481.8261) | 95.1845 (75.0587,116.5451) | 3333.8265 (2676.0824,4058.5123) | 91.9991 (73.8745,112.2572) | 0.169947 | -0.03347 | -0.10 (-0.35 to 0.14) | 0.414 |
| North Macedonia | 1678.2249 (1310.4661,2076.4706) | 90.2865 (70.4146,111.1756) | 1279.8704 (1021.5876,1607.1743) | 93.6588 (74.6732,116.2856) | -0.23737 | 0.037351 | 0.10 (0.02 to 0.19) | 0.019 |
| Northern Mariana Islands | 39.614 (31.1978,49.745) | 80.9591 (62.9403,102.5124) | 36.0694 (28.064,46.3013) | 93.706 (72.2595,120.2371) | -0.08948 | 0.157449 | 0.46 (0.33 to 0.59) | <0.001 |
| Palestine | 3356.4401 (2593.5196,4232.8715) | 105.4313 (82.0035,133.288) | 6713.7162 (5207.8774,8448.6278) | 109.0413 (84.6644,136.6464) | 1.000249 | 0.03424 | 0.09 (-0.03 to 0.21) | 0.143 |
| People's Democratic Republic of Algeria | 36779.3975 (28468.4361,47317.7817) | 106.3391 (82.5776,136.2249) | 48797.4529 (37952.1896,61725.3212) | 107.0966 (83.2787,135.4348) | 0.326761 | 0.007123 | 0.02 (-0.00 to 0.05) | 0.096 |
| People's Republic of Bangladesh | 204402.4086 (155982.3938,268295.8422) | 127.4196 (98.0958,166.2383) | 194674.2833 (149352.543,253356.3785) | 122.1334 (93.527,157.5896) | -0.04759 | -0.04149 | -0.15 (-0.30 to -0.00) | 0.045 |
| People's Republic of China | 694482.5266 (546100.0952,849282.1927) | 58.7649 (46.2758,71.8161) | 465693.317 (373699.2904,571972.394) | 51.0647 (40.9818,63.1982) | -0.32944 | -0.13103 | -0.45 (-0.53 to -0.37) | <0.001 |
| Plurinational State of Bolivia | 4419.6825 (3542.8956,5395.084) | 49.6814 (39.7697,60.5473) | 7049.9094 (5630.5523,8589.0982) | 57.7006 (46.1375,70.0801) | 0.595117 | 0.161413 | 0.46 (0.42 to 0.50) | <0.001 |
| Portuguese Republic | 4101.9356 (3286.7727,5061.0198) | 54.9956 (44.4528,67.9579) | 3154.2514 (2509.7343,3784.447) | 56.0048 (45.2505,67.1853) | -0.23103 | 0.018351 | 0.04 (-0.04 to 0.13) | 0.298 |
| Principality of Andorra | 26.85 (20.8492,33.0154) | 70.0512 (55.1098,87.4797) | 28.7973 (22.6127,35.8043) | 66.8903 (53.2211,83.1614) | 0.072525 | -0.04512 | -0.16 (-0.25 to -0.07) | <0.001 |
| Principality of Monaco | 10.3677 (8.0949,12.9179) | 65.3053 (51.39,80.062) | 13.1777 (10.4253,16.4163) | 65.5667 (52.1677,81.8763) | 0.271034 | 0.004003 | 0.00 (-0.03 to 0.03) | 0.872 |
| Puerto Rico | 2297.207 (1817.118,2796.6507) | 66.6889 (52.802,81.501) | 1272.4082 (990.9472,1589.9223) | 73.1385 (57.8582,89.7911) | -0.44611 | 0.096712 | 0.30 (0.20 to 0.39) | <0.001 |
| Republic of Albania | 4138.406 (3253.3186,5111.9447) | 106.4885 (83.8185,131.4639) | 1925.009 (1540.4163,2351.3395) | 110.3684 (87.9105,135.7759) | -0.53484 | 0.036435 | 0.12 (0.05 to 0.19) | <0.001 |
| Republic of Angola | 20361.5171 (15870.092,25285.6479) | 126.7578 (100.2183,158.4802) | 62400.3044 (47888.0884,79062.7969) | 128.8042 (99.5572,164.5811) | 2.06462 | 0.016144 | 0.10 (-0.12 to 0.32) | 0.354 |
| Republic of Armenia | 3237.9401 (2543.7406,4026.0566) | 88.0212 (69.193,109.7045) | 2082.2932 (1667.0226,2544.5064) | 95.7543 (76.2579,117.9087) | -0.35691 | 0.087855 | 0.28 (0.23 to 0.33) | <0.001 |
| Republic of Austria | 4618.2235 (3794.5914,5613.0325) | 82.1614 (67.5467,100.958) | 4839.7741 (3945.817,5890.5612) | 88.0573 (71.1835,107.4424) | 0.047973 | 0.07176 | 0.22 (0.12 to 0.33) | <0.001 |
| Republic of Azerbaijan | 7599.0176 (5910.415,9434.189) | 88.6474 (69.1073,109.9354) | 7827.0551 (6097.4797,9604.6726) | 92.4143 (72.2445,114.7075) | 0.030009 | 0.042493 | 0.17 (0.10 to 0.23) | <0.001 |
| Republic of Belarus | 9099.112 (7142.1649,11344.8455) | 103.4384 (80.7081,128.3967) | 5794.0876 (4675.382,7143.798) | 95.2549 (76.463,117.9678) | -0.36322 | -0.07911 | -0.28 (-0.35 to -0.21) | <0.001 |
| Republic of Benin | 7814.8595 (6155.5962,9726.3475) | 99.199 (79.5235,123.6221) | 19805.192 (15659.4069,25053.097) | 99.9669 (78.6854,126.9655) | 1.534299 | 0.007741 | 0.02 (-0.03 to 0.06) | 0.473 |
| Republic of Botswana | 2663.3155 (2073.6338,3362.9789) | 144.11 (113.3615,180.3663) | 4163.13 (3234.6675,5206.297) | 166.8142 (129.4015,208.5684) | 0.563138 | 0.157548 | 0.47 (0.42 to 0.51) | <0.001 |
| Republic of Bulgaria | 6038.5779 (4719.3786,7752.5072) | 95.9604 (75.1864,121.7586) | 3549.1052 (2819.9785,4472.5144) | 93.1019 (72.6755,118.2762) | -0.41226 | -0.02979 | -0.12 (-0.16 to -0.08) | <0.001 |
| Republic of Burundi | 11106.6317 (8705.6967,13915.0368) | 127.0626 (100.3848,158.6336) | 20536.3801 (16120.2598,26233.6441) | 109.0641 (85.5182,138.7587) | 0.84902 | -0.14165 | -0.48 (-0.58 to -0.38) | <0.001 |
| Republic of Cabo Verde | 488.3412 (374.6777,627.9299) | 96.4217 (74.8966,122.8452) | 532.1943 (418.8444,672.2128) | 101.9885 (79.897,126.3637) | 0.0898 | 0.057734 | 0.19 (0.13 to 0.24) | <0.001 |
| Republic of Cameroon | 15690.2077 (12231.4028,19391.42) | 96.8593 (76.2767,120.2304) | 43494.1827 (35000.2307,54121.122) | 99.4659 (80.0026,123.8753) | 1.772059 | 0.026911 | 0.08 (0.07 to 0.10) | <0.001 |
| Republic of Chad | 9334.8213 (7394.7547,11713.9829) | 95.5967 (76.0385,119.7546) | 27910.4517 (21899.3887,34878.9533) | 96.5877 (76.1312,120.2698) | 1.989929 | 0.010366 | 0.03 (0.01 to 0.06) | 0.005 |
| Republic of Chile | 11229.3918 (9017.6583,13583.0889) | 78.306 (62.9123,94.7025) | 13446.3888 (11094.9576,15776.4837) | 94.833 (78.1208,112.7973) | 0.197428 | 0.211057 | 0.63 (0.59 to 0.67) | <0.001 |
| Republic of Colombia | 26382.9746 (20889.5073,32595.3901) | 66.2457 (52.6862,81.9248) | 28460.8112 (22711.6378,34178.7212) | 70.1252 (56.2676,84.1599) | 0.078757 | 0.058562 | 0.18 (0.10 to 0.26) | <0.001 |
| Republic of Costa Rica | 2427.3987 (1896.2158,2932.4619) | 64.4536 (50.5576,77.555) | 2644.6905 (2118.9691,3201.6408) | 69.3982 (55.1815,83.6362) | 0.089516 | 0.076716 | 0.23 (0.20 to 0.27) | <0.001 |
| Republic of C么te d'Ivoire | 17882.8349 (14031.3044,22193.2912) | 93.8546 (74.0175,116.7476) | 37881.6031 (29353.5189,47811.6863) | 99.241 (77.1107,125.8945) | 1.118322 | 0.057391 | 0.18 (0.08 to 0.27) | <0.001 |
| Republic of Croatia | 2278.2292 (1846.8585,2790.3161) | 62.2266 (50.5565,75.8922) | 1847.4031 (1442.6924,2291.3815) | 79.4602 (62.8783,98.6255) | -0.18911 | 0.276949 | 0.79 (0.74 to 0.83) | <0.001 |
| Republic of Cuba | 6393.4181 (5228.935,7731.6384) | 64.7831 (52.7158,79.5993) | 4516.445 (3679.3118,5399.794) | 64.3559 (51.9278,77.217) | -0.29358 | -0.00659 | -0.02 (-0.07 to 0.03) | 0.475 |
| Republic of Cyprus | 551.2801 (437.1098,673.479) | 77.8358 (61.4351,95.0764) | 702.6749 (570.3303,860.6375) | 76.1548 (60.4532,93.6097) | 0.274624 | -0.0216 | -0.14 (-0.20 to -0.07) | <0.001 |
| Republic of Djibouti | 680.6667 (532.6027,859.6685) | 116.3557 (91.8381,145.5138) | 1684.7774 (1290.3685,2188.2602) | 117.7746 (90.1645,152.966) | 1.475187 | 0.012195 | -0.03 (-0.17 to 0.11) | 0.698 |
| Republic of Ecuador | 6977.6457 (5624.0191,8290.0951) | 54.4303 (44.1183,64.4502) | 11363.4885 (9584.7826,13560.1214) | 64.2392 (54.1559,76.8492) | 0.628556 | 0.18021 | 0.57 (0.49 to 0.65) | <0.001 |
| Republic of El Salvador | 4202.8108 (3328.3151,5122.2852) | 59.7463 (47.3744,72.2751) | 4484.4304 (3562.1154,5461.4029) | 70.5607 (55.9477,85.6849) | 0.067007 | 0.181005 | 0.51 (0.46 to 0.57) | <0.001 |
| Republic of Equatorial Guinea | 811.9247 (632.051,1035.693) | 123.0937 (95.6378,156.9306) | 2566.3807 (2004.2407,3280.2566) | 134.3932 (105.2881,170.5642) | 2.16086 | 0.091796 | 0.29 (0.14 to 0.43) | <0.001 |
| Republic of Estonia | 1344.8859 (1073.9451,1686.1988) | 103.729 (83.0497,130.1127) | 839.7036 (670.9053,1039.1499) | 100.4324 (80.3936,124.2692) | -0.37563 | -0.03178 | -0.16 (-0.21 to -0.12) | <0.001 |
| Republic of Fiji | 825.8118 (630.5877,1060.6689) | 88.8129 (68.3925,113.167) | 973.4584 (758.8026,1260.5602) | 104.4347 (81.6943,134.8353) | 0.17879 | 0.175896 | 0.51 (0.44 to 0.57) | <0.001 |
| Republic of Finland | 2324.183 (1865.903,2919.5686) | 62.1714 (49.5528,78.0239) | 2188.2827 (1731.0442,2728.0679) | 66.6325 (52.7619,82.9315) | -0.05847 | 0.071755 | 0.22 (0.16 to 0.28) | <0.001 |
| Republic of Ghana | 20499.4747 (16122.86,25380.0962) | 93.1024 (73.3092,115.0813) | 42751.2077 (33146.4489,54010.4965) | 99.3254 (77.9064,125.2395) | 1.085478 | 0.06684 | 0.21 (0.17 to 0.25) | <0.001 |
| Republic of Guatemala | 7782.9186 (6174.1173,9607.898) | 61.2659 (49.0675,74.9077) | 11846.5516 (9549.4347,14493.6882) | 70.4744 (56.926,85.7554) | 0.522122 | 0.150304 | 0.46 (0.44 to 0.49) | <0.001 |
| Republic of Guinea | 9267.0331 (7369.1382,11470.126) | 100.1437 (79.632,125.4873) | 19299.707 (15001.5355,23771.2098) | 99.2756 (77.0785,123.9394) | 1.08262 | -0.00867 | -0.03 (-0.06 to -0.01) | 0.013 |
| Republic of Guinea-Bissau | 1503.4329 (1183.6534,1885.6966) | 95.9298 (75.9784,118.5807) | 2748.1347 (2148.0502,3422.685) | 94.5276 (74.8573,117.4992) | 0.827906 | -0.01462 | -0.04 (-0.06 to -0.03) | <0.001 |
| Republic of Guyana | 669.5226 (527.6153,821.7734) | 65.402 (51.4789,79.8818) | 583.2494 (466.5206,713.9645) | 76.0691 (60.858,92.6661) | -0.12886 | 0.163101 | 0.49 (0.46 to 0.52) | <0.001 |
| Republic of Haiti | 6411.852 (5141.8997,7871.0933) | 69.9811 (56.0406,85.5541) | 11226.581 (9052.0977,13788.5184) | 74.5226 (60.1234,91.4028) | 0.750911 | 0.064896 | 0.20 (0.16 to 0.25) | <0.001 |
| Republic of Honduras | 4520.8982 (3589.8448,5468.9577) | 65.0475 (52.2926,78.5099) | 7512.7636 (5995.5406,9182.7039) | 66.8751 (53.4197,81.3527) | 0.661786 | 0.028096 | 0.09 (0.06 to 0.13) | <0.001 |
| Republic of Iceland | 173.5323 (136.2537,217.4057) | 75.1101 (58.8974,94.6924) | 181.6173 (146.6363,223.0171) | 71.6555 (57.9069,87.9991) | 0.046591 | -0.04599 | -0.15 (-0.19 to -0.12) | <0.001 |
| Republic of India | 1215701.733 (942610.8241,1546867.831) | 111.5758 (86.7655,141.2396) | 1495503.5443 (1167384.8502,1921195.4888) | 114.9307 (89.904,146.6652) | 0.230157 | 0.030068 | 0.10 (0.06 to 0.14) | <0.001 |
| Republic of Indonesia | 96893.1875 (75093.0738,123715.0837) | 42.605 (33.1333,54.31) | 106117.4051 (82741.3628,133833.6697) | 43.9035 (34.2843,54.7892) | 0.0952 | 0.030478 | 0.07 (0.02 to 0.13) | 0.012 |
| Republic of Iraq | 28629.954 (22559.8709,36554.9187) | 106.0908 (83.2826,135.3552) | 48046.8055 (37870.3402,59624.3955) | 105.5828 (83.3556,130.8266) | 0.678201 | -0.00479 | -0.05 (-0.14 to 0.05) | 0.324 |
| Republic of Italy | 30195.0925 (24108.948,36468.5203) | 82.1465 (65.286,100.6271) | 21954.9531 (18339.4272,25669.106) | 71.5759 (59.2243,83.4752) | -0.2729 | -0.12868 | -0.43 (-0.49 to -0.37) | <0.001 |
| Republic of Kazakhstan | 16154.4274 (12588.1284,19958.9632) | 89.4342 (69.6183,110.2591) | 17945.699 (14174.9896,22423.4094) | 95.1619 (75.1449,119.093) | 0.110884 | 0.064044 | 0.21 (0.17 to 0.26) | <0.001 |
| Republic of Kenya | 43049.1863 (33840.9833,53267.9558) | 116.6728 (92.8859,144.4682) | 67447.9927 (52870.9856,83115.0913) | 110.4038 (87.34,136.6215) | 0.566766 | -0.05373 | -0.17 (-0.21 to -0.13) | <0.001 |
| Republic of Kiribati | 76.4437 (60.0997,96.3121) | 75.0947 (59.4476,93.9364) | 123.1008 (96.9959,156.7632) | 87.8871 (69.5827,111.412) | 0.610346 | 0.17035 | 0.50 (0.45 to 0.55) | <0.001 |
| Republic of Korea | 40240.5292 (31346.8628,50043.9325) | 96.0745 (75.54,118.6692) | 28850.1539 (22903.0999,36066.8779) | 107.6741 (85.9001,134.5337) | -0.28306 | 0.120735 | 0.35 (0.32 to 0.38) | <0.001 |
| Republic of Latvia | 2320.2772 (1862.4048,2857.4488) | 107.46 (86.1525,132.5499) | 1215.3266 (980.8812,1509.4144) | 105.8393 (84.8122,131.8696) | -0.47621 | -0.01508 | -0.07 (-0.09 to -0.04) | <0.001 |
| Republic of Liberia | 4054.6081 (3191.5777,5048.8029) | 106.7238 (85.4622,133.3131) | 7037.4226 (5496.5763,8781.7856) | 98.6113 (77.8503,122.5719) | 0.73566 | -0.07601 | -0.23 (-0.42 to -0.03) | 0.022 |
| Republic of Lithuania | 3186.9452 (2537.875,3980.5456) | 102.1231 (81.533,128.3168) | 1497.9094 (1191.6369,1867.7165) | 90.6119 (71.9674,113.8692) | -0.52999 | -0.11272 | -0.38 (-0.47 to -0.29) | <0.001 |
| Republic of Madagascar | 20966.0884 (16444.7015,26464.4924) | 115.9265 (90.629,146.5979) | 40361.4729 (31540.8146,51096.5581) | 106.4201 (83.659,134.9442) | 0.925084 | -0.082 | -0.27 (-0.33 to -0.21) | <0.001 |
| Republic of Malawi | 19722.9258 (15547.7601,25049.2601) | 126.6521 (100.4341,159.541) | 29862.055 (23386.8296,37713.1319) | 114.6795 (89.7909,144.0468) | 0.514078 | -0.09453 | -0.31 (-0.39 to -0.23) | <0.001 |
| Republic of Maldives | 174.1913 (135.089,217.514) | 50.0681 (38.9418,62.5576) | 212.5263 (163.2872,273.8856) | 53.1308 (40.7413,67.7952) | 0.220074 | 0.061171 | 0.17 (0.08 to 0.26) | <0.001 |
| Republic of Mali | 13658.8206 (10755.9218,17230.801) | 98.8797 (77.9779,124.6399) | 36759.725 (28658.8121,46027.2398) | 97.755 (76.1251,122.5083) | 1.691281 | -0.01137 | -0.07 (-0.18 to 0.04) | 0.222 |
| Republic of Malta | 204.4624 (161.3809,253.232) | 65.1113 (51.5752,80.8435) | 186.3712 (146.7109,224.4638) | 72.2615 (57.0809,87.3762) | -0.08848 | 0.109815 | 0.25 (0.11 to 0.39) | <0.001 |
| Republic of Mauritius | 576.195 (454.0594,723.3589) | 49.8708 (39.339,61.9222) | 541.3796 (419.6842,701.1073) | 63.5991 (49.0972,81.4622) | -0.06042 | 0.275277 | 0.79 (0.72 to 0.86) | <0.001 |
| Republic of Moldova | 5198.2623 (4108.3543,6486.4538) | 120.6576 (95.6411,150.3687) | 2305.3467 (1842.3254,2863.8621) | 105.1186 (83.8826,130.226) | -0.55652 | -0.12879 | -0.45 (-0.49 to -0.41) | <0.001 |
| Republic of Mozambique | 24541.6244 (19351.3237,31064.909) | 120.8211 (96.0632,152.6555) | 51618.7371 (39542.6823,65543.7653) | 113.9718 (88.0366,143.3298) | 1.103314 | -0.05669 | -0.14 (-0.27 to -0.01) | 0.038 |
| Republic of Namibia | 2865.6438 (2280.5648,3637.6091) | 147.5044 (117.4331,185.5499) | 4366.3901 (3451.1831,5573.1252) | 156.4725 (123.6746,198.6144) | 0.523703 | 0.060799 | 0.21 (0.12 to 0.30) | <0.001 |
| Republic of Nauru | 9.5289 (7.5049,11.8422) | 67.456 (53.6578,83.9702) | 11.4993 (8.9931,14.7739) | 85.872 (67.4399,109.7303) | 0.206781 | 0.273008 | 0.81 (0.77 to 0.85) | <0.001 |
| Republic of Nicaragua | 3489.3988 (2713.43,4288.3221) | 61.5618 (47.6892,75.0609) | 4608.4421 (3720.7017,5556.8942) | 66.7518 (54.1024,80.7505) | 0.320698 | 0.084306 | 0.28 (0.22 to 0.33) | <0.001 |
| Republic of Niue | 1.9841 (1.5613,2.5392) | 77.8202 (61.9646,98.1) | 1.4087 (1.0985,1.8142) | 103.824 (81.529,132.9131) | -0.29001 | 0.334152 | 0.90 (0.79 to 1.00) | <0.001 |
| Republic of Palau | 12.2198 (9.594,15.6088) | 76.1351 (59.9986,96.4477) | 10.7934 (8.4848,13.7154) | 87.4416 (68.4944,112.2512) | -0.11673 | 0.148506 | 0.54 (0.38 to 0.69) | <0.001 |
| Republic of Panama | 1947.7757 (1536.0874,2360.8632) | 69.2037 (54.6983,83.6968) | 2886.3094 (2304.5005,3473.8101) | 71.9254 (57.3305,86.5571) | 0.481849 | 0.039329 | 0.14 (0.09 to 0.19) | <0.001 |
| Republic of Paraguay | 2455.6237 (1950.8555,3111.8011) | 45.165 (35.8908,56.5272) | 3580.9643 (2838.6589,4378.2374) | 50.2276 (39.8987,61.2816) | 0.458271 | 0.112091 | 0.31 (0.15 to 0.46) | <0.001 |
| Republic of Peru | 13322.3986 (10504.0926,16543.2226) | 48.1015 (37.8594,59.3265) | 19742.9027 (15659.2715,24198.322) | 56.8878 (45.0134,69.9474) | 0.481933 | 0.182662 | 0.55 (0.52 to 0.58) | <0.001 |
| Republic of Poland | 11542.6185 (8590.7322,14792.9693) | 37.728 (28.2707,48.5311) | 10471.6917 (8193.5838,13235.9219) | 49.8094 (39.1442,63.073) | -0.09278 | 0.320224 | 0.90 (0.73 to 1.07) | <0.001 |
| Republic of Rwanda | 13292.2766 (10241.288,16673.1332) | 119.6267 (92.45,149.9792) | 18305.8957 (14027.3348,23387.8894) | 110.1 (85.2067,140.3646) | 0.377183 | -0.07964 | -0.26 (-0.33 to -0.18) | <0.001 |
| Republic of San Marino | 11.2502 (8.5531,14.047) | 68.5265 (52.9067,86.003) | 12.283 (9.7337,15.37) | 69.1545 (54.6167,85.2198) | 0.091803 | 0.009164 | 0.02 (-0.01 to 0.05) | 0.138 |
| Republic of Senegal | 11626.6502 (9030.5856,14623.8235) | 97.4383 (76.9835,122.8114) | 21001.5707 (16514.421,26093.7664) | 100.9983 (79.3922,125.189) | 0.80633 | 0.036536 | 0.14 (-0.02 to 0.29) | 0.078 |
| Republic of Serbia | 7522.1231 (5914.3604,9359.5447) | 97.1447 (76.3563,120.0186) | 4759.4213 (3711.6691,5947.16) | 88.6615 (69.5618,110.1491) | -0.36728 | -0.08733 | -0.30 (-0.33 to -0.27) | <0.001 |
| Republic of Seychelles | 39.0034 (30.5086,49.0536) | 47.855 (37.4803,60.0856) | 55.1034 (43.3623,70.0052) | 63.6145 (49.7243,80.159) | 0.412785 | 0.329318 | 0.93 (0.89 to 0.97) | <0.001 |
| Republic of Sierra Leone | 6660.6985 (5200.0786,8386.1357) | 104.1735 (81.2063,130.4188) | 11986.9138 (9457.0344,15063.3003) | 98.9397 (78.6041,124.5532) | 0.799648 | -0.05024 | -0.17 (-0.23 to -0.11) | <0.001 |
| Republic of Singapore | 3247.8206 (2604.3004,4039.1821) | 124.6349 (98.1851,156.9735) | 5449.8653 (4338.812,6844.3875) | 159.3018 (123.6882,204.186) | 0.678007 | 0.278148 | 0.77 (0.67 to 0.86) | <0.001 |
| Republic of Slovenia | 1302.4082 (1023.4612,1643.5377) | 86.6042 (68.2415,109.3023) | 939.2027 (751.859,1164.2124) | 78.913 (62.6149,98.0442) | -0.27887 | -0.08881 | -0.29 (-0.39 to -0.20) | <0.001 |
| Republic of South Africa | 71507.2358 (56588.4724,90953.9629) | 155.1217 (123.0757,196.7877) | 89721.9103 (71524.0663,112673.5235) | 165.6315 (131.1005,206.6753) | 0.254725 | 0.067752 | 0.21 (0.15 to 0.27) | <0.001 |
| Republic of South Sudan | 10386.0535 (8145.6256,13258.0016) | 118.3706 (92.4833,150.8693) | 15892.9043 (12084.1822,20170.8836) | 114.4368 (88.2613,144.8798) | 0.530216 | -0.03323 | -0.11 (-0.17 to -0.05) | <0.001 |
| Republic of Sudan | 30129.4046 (23643.8664,37817.0968) | 101.0568 (79.2447,126.7371) | 59418.0718 (46386.2853,75497.0741) | 109.0878 (85.2468,137.9529) | 0.972096 | 0.07947 | 0.24 (0.21 to 0.27) | <0.001 |
| Republic of Suriname | 282.847 (225.0698,352.1292) | 63.5963 (50.7147,79.0377) | 370.2688 (295.3905,449.9449) | 73.3663 (58.4331,88.7424) | 0.309078 | 0.153625 | 0.46 (0.42 to 0.49) | <0.001 |
| Republic of Tajikistan | 6634.5484 (5126.3081,8324.6601) | 83.5007 (65.0218,104.0105) | 11263.6677 (8838.5977,13933.0086) | 91.4772 (71.4224,112.9796) | 0.697729 | 0.095526 | 0.29 (0.22 to 0.35) | <0.001 |
| Republic of the Congo | 4400.0241 (3454.9016,5685.0479) | 127.4501 (99.4227,163.1501) | 8160.8596 (6360.1483,10427.5061) | 128.9455 (100.7871,164.2113) | 0.854731 | 0.011733 | 0.06 (-0.01 to 0.12) | 0.088 |
| Republic of the Gambia | 1380.3031 (1096.936,1727.2915) | 90.612 (71.7848,114.1281) | 2980.5941 (2331.676,3700.4812) | 91.7791 (72.2234,114.0213) | 1.159377 | 0.01288 | 0.05 (-0.01 to 0.11) | 0.111 |
| Republic of the Marshall Islands | 54.2984 (41.6608,71.4206) | 81.503 (63.6607,105.7707) | 64.7493 (50.7274,84.0777) | 107.8133 (84.7801,139.3878) | 0.192472 | 0.322814 | 0.90 (0.85 to 0.95) | <0.001 |
| Republic of the Niger | 13008.2989 (10133.595,16352.0629) | 96.008 (75.6107,121.3125) | 37444.2909 (29680.4385,46066.3199) | 91.7375 (73.1945,113.7957) | 1.878493 | -0.04448 | -0.13 (-0.16 to -0.10) | <0.001 |
| Republic of the Philippines | 39268.6651 (30728.0807,49481.3303) | 45.3813 (35.6734,57.0955) | 55436.1776 (43720.0124,69682.679) | 47.0775 (37.1925,59.141) | 0.411715 | 0.037377 | 0.11 (0.08 to 0.14) | <0.001 |
| Republic of the Union of Myanmar | 24210.965 (18213.4491,31214.7903) | 48.6522 (36.53,62.7819) | 29007.5955 (22103.4031,36693.045) | 52.7133 (40.0952,66.557) | 0.198118 | 0.083472 | 0.30 (0.11 to 0.49) | 0.002 |
| Republic of Trinidad and Tobago | 953.8205 (752.2287,1173.992) | 70.7724 (56.067,87.091) | 763.0553 (603.6986,920.2546) | 76.529 (60.7832,92.4205) | -0.2 | 0.08134 | 0.26 (0.22 to 0.29) | <0.001 |
| Republic of Tunisia | 10951.3406 (8689.7945,13744.3282) | 106.3793 (85.054,133.4214) | 10863.9091 (8522.6886,13948.7938) | 108.1774 (85.3273,138.8193) | -0.00798 | 0.016903 | 0.06 (0.03 to 0.08) | <0.001 |
| Republic of Turkey | 65450.7699 (50264.3383,82478.0356) | 95.3697 (73.4055,120.3707) | 63402.8639 (48976.9525,81200.0472) | 93.2934 (71.6002,118.4338) | -0.03129 | -0.02177 | -0.07 (-0.14 to -0.01) | 0.019 |
| Republic of Uganda | 34456.5828 (27203.7054,43441.7593) | 120.7721 (96.0318,151.3565) | 77039.1573 (59229.4876,97755.2654) | 120.7617 (94.0799,152.7905) | 1.235833 | -8.61E-05 | -0.01 (-0.05 to 0.03) | 0.554 |
| Republic of Uzbekistan | 22749.565 (17690.7654,28378.2683) | 78.1554 (61.5474,96.7317) | 32145.6003 (25416.0532,39598.1145) | 89.0526 (70.1061,109.6627) | 0.41302 | 0.13943 | 0.44 (0.38 to 0.51) | <0.001 |
| Republic of Vanuatu | 149.5055 (114.3856,191.8201) | 66.101 (50.7801,85.0871) | 307.7841 (239.6098,390.0081) | 79.0995 (61.7254,100.6758) | 1.058681 | 0.196646 | 0.61 (0.57 to 0.65) | <0.001 |
| Republic of Yemen | 20216.7449 (15896.7733,25920.7263) | 90.2135 (71.5742,114.6192) | 40674.5778 (31552.9869,51765.4195) | 92.4001 (72.0649,117.2675) | 1.011925 | 0.024238 | 0.08 (0.05 to 0.11) | <0.001 |
| Republic of Zambia | 15835.9683 (12212.4859,20367.9541) | 126.1162 (98.4036,160.429) | 30576.2924 (23824.7725,39227.0481) | 114.3602 (89.1696,145.9102) | 0.930813 | -0.09322 | -0.30 (-0.37 to -0.22) | <0.001 |
| Republic of Zimbabwe | 22011.34 (17301.9693,28281.0511) | 146.2764 (115.1798,187.7006) | 33365.1668 (25770.3127,42810.0114) | 165.0234 (127.9546,211.2614) | 0.515817 | 0.128161 | 0.38 (0.34 to 0.43) | <0.001 |
| Romania | 15842.4884 (12339.7212,20296.2659) | 81.0354 (63.3454,103.5568) | 8834.8178 (6972.9065,10950.9714) | 77.423 (61.1718,96.1992) | -0.44233 | -0.04458 | -0.15 (-0.17 to -0.14) | <0.001 |
| Russian Federation | 200286.4082 (160585.8919,248583.9679) | 159.529 (128.0504,197.682) | 134038.076 (107944.9901,164037.7578) | 140.6849 (113.2081,172.2499) | -0.33077 | -0.11812 | -0.41 (-0.44 to -0.38) | <0.001 |
| Saint Kitts and Nevis | 28.418 (22.4857,35.7102) | 60.5782 (48.1645,75.7258) | 26.6287 (21.2711,32.781) | 65.0924 (51.8674,80.1824) | -0.06296 | 0.074519 | 0.20 (0.15 to 0.25) | <0.001 |
| Saint Lucia | 120.5521 (95.8288,147.8095) | 71.0602 (57.0494,86.3778) | 91.3861 (73.1895,112.9113) | 76.2557 (61.626,94.2628) | -0.24194 | 0.073114 | 0.22 (0.19 to 0.25) | <0.001 |
| Saint Vincent and the Grenadines | 93.6994 (74.7865,116.153) | 71.0934 (56.892,87.4205) | 69.2768 (55.9005,85.3397) | 77.7096 (63.0881,95.7643) | -0.26065 | 0.093063 | 0.27 (0.25 to 0.29) | <0.001 |
| Slovak Republic | 4657.3009 (3641.2036,5815.9085) | 101.7123 (79.9037,127.0932) | 3479.2745 (2754.5422,4328.3985) | 104.4247 (82.7587,130.4398) | -0.25294 | 0.026667 | 0.05 (-0.11 to 0.22) | 0.521 |
| Socialist Republic of Viet Nam | 40967.2565 (31405.0146,52731.2152) | 46.4454 (35.6321,59.6531) | 45524.7943 (34930.971,57779.2564) | 51.7461 (39.6603,65.4627) | 0.111248 | 0.114128 | 0.36 (0.30 to 0.43) | <0.001 |
| Solomon Islands | 340.4435 (267.2376,430.8203) | 66.8382 (52.7631,83.8878) | 678.9834 (536.3804,856.9927) | 78.2108 (62.0237,99.1294) | 0.994408 | 0.170151 | 0.55 (0.30 to 0.80) | <0.001 |
| State of Eritrea | 5746.6384 (4507.2076,7258.9706) | 111.0803 (86.7843,140.5976) | 9000.6216 (7046.8524,11415.2436) | 106.3993 (83.3162,135.0199) | 0.566241 | -0.04214 | -0.09 (-0.18 to -0.00) | 0.044 |
| State of Israel | 4042.1894 (3132.8445,5131.2454) | 77.9409 (60.5517,98.456) | 6519.8611 (5252.8874,7969.5309) | 71.7822 (57.8731,87.7669) | 0.612953 | -0.07902 | -0.25 (-0.35 to -0.15) | <0.001 |
| State of Kuwait | 2377.2362 (1814.8638,3024.2677) | 120.7972 (92.3586,153.8497) | 4748.1434 (3661.2883,6145.6418) | 134.854 (103.1536,173.453) | 0.997338 | 0.116367 | 0.32 (0.22 to 0.43) | <0.001 |
| State of Libya | 5948.6189 (4597.4882,7544.6144) | 102.5747 (79.6549,129.621) | 6612.5038 (5169.6949,8404.8831) | 112.0188 (88.2523,140.911) | 0.111603 | 0.09207 | 0.24 (0.14 to 0.34) | <0.001 |
| State of Qatar | 551.0285 (428.4322,706.7688) | 113.6113 (86.7578,147.8436) | 3176.5187 (2479.9923,4082.0925) | 132.8408 (103.4784,172.9397) | 4.764709 | 0.169257 | 0.50 (0.45 to 0.54) | <0.001 |
| Sultanate of Oman | 2378.2983 (1879.0039,2994.0285) | 85.8589 (66.6799,108.4747) | 4377.5373 (3430.8267,5527.0876) | 94.6543 (74.1347,121.3876) | 0.840617 | 0.10244 | 0.31 (0.27 to 0.36) | <0.001 |
| Swiss Confederation | 4691.2647 (3789.6873,5860.1348) | 97.7457 (79.3265,123.5186) | 4908.928 (3919.5771,6068.1146) | 89.905 (71.6949,110.2566) | 0.046398 | -0.08022 | -0.25 (-0.31 to -0.19) | <0.001 |
| Syrian Arab Republic | 16510.444 (12696.9648,21237.7186) | 88.5683 (68.2669,114.7681) | 11755.547 (8937.0471,15334.6129) | 91.6817 (71.3578,118.1314) | -0.28799 | 0.035153 | 0.12 (0.10 to 0.14) | <0.001 |
| Taiwan (Province of China) | 13542.4961 (10588.3035,16759.4372) | 71.0284 (55.4105,88.0366) | 9192.7928 (7293.2095,11508.3273) | 74.6549 (59.1599,94.0225) | -0.32119 | 0.051057 | 0.23 (0.07 to 0.38) | 0.006 |
| Togolese Republic | 5234.6738 (4110.1082,6545.013) | 91.5004 (72.2062,114.3046) | 9833.0836 (7597.7888,12237.2652) | 91.0171 (70.6623,113.5397) | 0.878452 | -0.00528 | 0.01 (-0.09 to 0.12) | 0.785 |
| Tokelau | 1.2506 (0.969,1.5731) | 65.8565 (51.7622,82.5785) | 0.9881 (0.7697,1.2908) | 79.1786 (61.8161,100.9053) | -0.2099 | 0.20229 | 0.59 (0.56 to 0.63) | <0.001 |
| Turkmenistan | 4405.0553 (3441.1758,5507.3236) | 85.2514 (66.6877,106.1312) | 5200.2761 (4129.0941,6346.2827) | 97.0282 (77.038,118.4269) | 0.180525 | 0.138142 | 0.50 (0.41 to 0.59) | <0.001 |
| Tuvalu | 8.9637 (6.9924,11.3) | 71.6731 (55.685,91.2096) | 10.2843 (8.096,13.054) | 79.3158 (62.3941,100.5483) | 0.147328 | 0.106633 | 0.33 (0.27 to 0.40) | <0.001 |
| Ukraine | 38822.8885 (30733.1845,47857.5094) | 94.1633 (74.9131,116.0988) | 21800.9823 (17136.0098,26761.2338) | 88.1914 (69.7846,107.9646) | -0.43845 | -0.06342 | -0.24 (-0.33 to -0.15) | <0.001 |
| Union of the Comoros | 786.2601 (613.536,981.8159) | 112.9827 (88.5952,140.8189) | 892.6068 (704.5347,1134.5849) | 108.3903 (85.7445,137.4114) | 0.135256 | -0.04065 | -0.16 (-0.23 to -0.09) | <0.001 |
| United Arab Emirates | 2247.6759 (1774.5772,2827.25) | 103.4587 (81.0212,130.7616) | 6599.5758 (5189.7162,8442.1083) | 104.4564 (81.5157,132.9511) | 1.936178 | 0.009643 | 0.02 (-0.11 to 0.16) | 0.709 |
| United Kingdom of Great Britain and Northern Ireland | 33649.2768 (27004.3373,40874.9896) | 79.5625 (63.4873,97.0817) | 35989.0117 (29240.2148,43734.8591) | 81.1475 (65.7896,98.2806) | 0.069533 | 0.019921 | 0.07 (0.03 to 0.11) | 0.002 |
| United Mexican States | 109736.0923 (87402.363,132913.3665) | 98.3389 (78.9091,118.9638) | 109209.7507 (89363.2842,130828.519) | 95.503 (78.1392,114.4663) | -0.0048 | -0.02884 | -0.10 (-0.18 to -0.01) | 0.022 |
| United Republic of Tanzania | 48818.425 (37701.231,61108.9548) | 120.4269 (93.1348,152.0524) | 95089.8343 (73811.1663,118070.5698) | 119.1671 (92.7657,147.0852) | 0.947827 | -0.01046 | -0.04 (-0.09 to 0.01) | 0.092 |
| United States of America | 134477.3461 (109053.6325,161182.489) | 63.1302 (51.1371,75.7567) | 139760.7901 (113583.3196,167889.723) | 62.3186 (50.5201,75.2698) | 0.039289 | -0.01286 | 0.03 (-0.31 to 0.37) | 0.866 |
| United States Virgin Islands | 77.6366 (61.9745,95.0291) | 70.5876 (56.4055,86.1818) | 35.37 (28.6755,43.2449) | 70.9077 (57.3498,87.3675) | -0.54442 | 0.004535 | 0.03 (-0.01 to 0.08) | 0.132 |

**Table S2 Numbers and ASRs per 100,000 Cases of incidence of urogenital congenital anomalies in 1990 and 2021, along with the relative changes and AAPC in ASRs per 100,000 Cases from 1990-2021, Categorized by 204 countries and territories**

| Characteristic | Number in 1990  （95% CI） | Age-standardized  Rate in 1990  （95% CI） | Number in 2019  （95% CI） | Age-standardized  Rate in 2019  （95% CI） | Relative Change of numbers from 1990 to 2019（%） | Relative Change of age-standardized  rate from 1990 to 2019（%） | AAPC  (Age-standardized  Rate, 95% CI) | *P* value |
| --- | --- | --- | --- | --- | --- | --- | --- | --- |
| American Samoa | 13.2113 (9.636,17.7677) | 15.6812 (11.4375,21.0895) | 7.2352 (5.3256,10.0498) | 22.4738 (16.5421,31.2162) | -0.45235 | 0.433168 | 1.15 (1.06 to 1.24) | <0.001 |
| Antigua and Barbuda | 5.8567 (4.177,8.2836) | 9.9994 (7.1315,14.1429) | 5.1881 (3.7358,7.1465) | 10.3432 (7.4479,14.2474) | -0.11416 | 0.034382 | 0.19 (-0.07 to 0.45) | 0.153 |
| Arab Republic of Egypt | 16935.633 (12291.7416,22970.0292) | 18.7456 (13.6054,25.4249) | 14735.9504 (11187.3901,19405.0899) | 11.9122 (9.0436,15.6866) | -0.12988 | -0.36453 | -1.37 (-1.67 to -1.07) | <0.001 |
| Argentine Republic | 5683.9899 (4167.7029,7562.2938) | 17.034 (12.49,22.663) | 5089.7284 (3735.3553,6748.6842) | 19.7578 (14.5002,26.1977) | -0.10455 | 0.159904 | 0.47 (0.28 to 0.67) | <0.001 |
| Australia | 2185.8952 (1752.9152,2877.7666) | 17.6862 (14.183,23.2842) | 2242.6191 (1602.4817,3087.1189) | 15.7455 (11.2511,21.6748) | 0.02595 | -0.10973 | -0.29 (-0.44 to -0.14) | <0.001 |
| Barbados | 31.0753 (23.3477,40.5547) | 15.5205 (11.661,20.255) | 20.6096 (15.4846,27.022) | 16.3222 (12.2634,21.4006) | -0.33679 | 0.051654 | 0.13 (0.02 to 0.25) | 0.018 |
| Belize | 37.9402 (28.611,49.1358) | 12.5389 (9.4557,16.239) | 44.4758 (32.2142,60.7503) | 12.0835 (8.7522,16.5051) | 0.172261 | -0.03632 | -0.23 (-0.46 to -0.00) | 0.047 |
| Bermuda | 5.7165 (4.2413,7.645) | 13.4085 (9.9484,17.9321) | 2.8752 (2.0427,3.8901) | 12.2608 (8.7108,16.5887) | -0.49703 | -0.08559 | -0.28 (-0.37 to -0.20) | <0.001 |
| Bolivarian Republic of Venezuela | 2570.3613 (1942.3471,3407.3515) | 9.7348 (7.3563,12.9047) | 2235.1806 (1642.9247,3017.5273) | 10.2991 (7.5701,13.9039) | -0.1304 | 0.057967 | 0.21 (-0.12 to 0.53) | 0.213 |
| Bosnia and Herzegovina | 566.9254 (433.9215,731.8423) | 17.4504 (13.3564,22.5267) | 269.3464 (187.7983,387.7835) | 20.155 (14.0528,29.0175) | -0.5249 | 0.154988 | 0.49 (0.30 to 0.69) | <0.001 |
| Brunei Darussalam | 63.3131 (47.2039,85.6542) | 18.7435 (13.9745,25.3575) | 72.866 (54.9947,98.2341) | 24.6887 (18.6335,33.284) | 0.150883 | 0.317187 | 0.89 (0.82 to 0.97) | <0.001 |
| Burkina Faso | 4374.3203 (3218.2229,5809.161) | 19.6914 (14.4871,26.1505) | 8727.1331 (6443.9427,11528.0009) | 19.1668 (14.1524,25.3182) | 0.995083 | -0.02664 | -0.07 (-0.23 to 0.09) | 0.374 |
| Canada | 3075.826 (2686.1914,3503.7234) | 15.7658 (13.7687,17.9591) | 2459.3894 (1797.5189,3334.1074) | 13.8665 (10.1347,18.7983) | -0.20041 | -0.12047 | -0.43 (-0.58 to -0.29) | <0.001 |
| Central African Republic | 1620.0027 (1190.789,2238.1394) | 26.7425 (19.6572,36.9465) | 2300.1926 (1701.6956,3056.7416) | 25.2965 (18.7145,33.6167) | 0.41987 | -0.05407 | -0.16 (-0.25 to -0.07) | <0.001 |
| Commonwealth of Dominica | 8.338 (5.979,11.5342) | 9.3128 (6.678,12.8827) | 3.2684 (2.3526,4.4735) | 10.5381 (7.5853,14.4236) | -0.60801 | 0.131572 | 0.44 (0.33 to 0.56) | <0.001 |
| Commonwealth of the Bahamas | 29.8848 (21.3638,40.4743) | 11.249 (8.0416,15.235) | 21.327 (15.6598,29.0155) | 11.1036 (8.1531,15.1066) | -0.28636 | -0.01293 | -0.02 (-0.09 to 0.05) | 0.52 |
| Cook Islands | 2.7522 (2.0968,3.5949) | 13.0063 (9.9087,16.9886) | 1.5769 (1.1546,2.2761) | 14.9589 (10.9529,21.5915) | -0.42704 | 0.150127 | 0.37 (0.21 to 0.53) | <0.001 |
| Czech Republic | 1228.1951 (916.9733,1666.9679) | 19.8344 (14.8084,26.9203) | 878.133 (621.5073,1199.0247) | 17.1688 (12.1514,23.4427) | -0.28502 | -0.13439 | -0.43 (-0.49 to -0.37) | <0.001 |
| Democratic People's Republic of Korea | 3214.0113 (2351.2811,4235.0643) | 12.4605 (9.1157,16.419) | 1347.9913 (987.6848,1801.6761) | 9.546 (6.9945,12.7589) | -0.58059 | -0.2339 | -0.86 (-0.93 to -0.78) | <0.001 |
| Democratic Republic of Sao Tome and Principe | 40.1225 (29.8415,52.9685) | 18.0916 (13.4558,23.884) | 40.213 (29.645,53.1681) | 16.9839 (12.5206,22.4555) | 0.002256 | -0.06123 | -0.23 (-0.29 to -0.17) | <0.001 |
| Democratic Republic of the Congo | 22958.7412 (16944.829,30995.5827) | 26.6942 (19.7018,36.0387) | 32439.8668 (23474.2186,44710.3062) | 23.7247 (17.1678,32.6987) | 0.412964 | -0.11124 | -0.39 (-0.45 to -0.32) | <0.001 |
| Democratic Republic of Timor-Leste | 168.5728 (125.6744,227.2116) | 10.1073 (7.5352,13.6231) | 174.6664 (125.793,237.7943) | 8.9189 (6.4233,12.1424) | 0.036148 | -0.11758 | -0.38 (-0.56 to -0.19) | <0.001 |
| Democratic Socialist Republic of Sri Lanka | 1691.7933 (1213.4477,2355.4342) | 9.8056 (7.0331,13.652) | 1443.4283 (1068.3159,1968.1452) | 9.9629 (7.3738,13.5846) | -0.14681 | 0.016042 | 0.02 (-0.17 to 0.22) | 0.813 |
| Dominican Republic | 1055.2499 (743.9627,1458.652) | 9.793 (6.9042,13.5367) | 1026.2306 (740.1576,1434.9613) | 10.0114 (7.2206,13.9988) | -0.0275 | 0.022302 | 0.07 (0.01 to 0.12) | 0.017 |
| Eastern Republic of Uruguay | 445.2341 (331.1755,586.4537) | 16.5771 (12.3304,21.835) | 240.8872 (175.3418,326.0398) | 14.0936 (10.2587,19.0756) | -0.45897 | -0.14982 | -0.47 (-0.80 to -0.14) | 0.005 |
| Federal Democratic Republic of Ethiopia | 29845.1244 (22140.692,40676.61) | 25.5523 (18.956,34.8258) | 35437.0919 (26399.5146,47280.6576) | 21.2235 (15.8109,28.3167) | 0.187366 | -0.16941 | -0.58 (-0.64 to -0.51) | <0.001 |
| Federal Democratic Republic of Nepal | 7478.3543 (5286.8701,10714.7375) | 19.8257 (14.0159,28.4056) | 5475.4437 (3931.6747,7576.7756) | 17.7766 (12.7646,24.5988) | -0.26783 | -0.10336 | -0.31 (-0.65 to 0.03) | 0.073 |
| Federal Republic of Germany | 6020.845 (5078.6835,7087.2735) | 14.22 (11.9948,16.7387) | 5453.3117 (4419.501,6630.8919) | 14.4281 (11.6929,17.5436) | -0.09426 | 0.014634 | 0.02 (-0.14 to 0.18) | 0.834 |
| Federal Republic of Nigeria | 42030.8337 (31727.7699,55863.5526) | 21.4543 (16.1952,28.5151) | 81778.5217 (60417.1721,107709.1477) | 20.6688 (15.2699,27.2225) | 0.945679 | -0.03661 | -0.15 (-0.36 to 0.07) | 0.178 |
| Federal Republic of Somalia | 4022.0462 (2971.363,5558.7752) | 21.6782 (16.0152,29.961) | 8361.424 (6278.4555,11277.8771) | 18.1427 (13.623,24.4708) | 1.078898 | -0.16309 | -0.59 (-0.89 to -0.29) | <0.001 |
| Federated States of Micronesia | 19.2392 (14.0969,25.9927) | 12.7136 (9.3155,17.1765) | 12.688 (9.1738,16.8165) | 14.1874 (10.2579,18.8038) | -0.34051 | 0.115923 | 0.22 (0.04 to 0.39) | 0.014 |
| Federative Republic of Brazil | 20420.5094 (15223.4329,26592.5881) | 13.1808 (9.8262,17.1647) | 19703.6515 (14832.2803,26168.8097) | 12.3848 (9.3229,16.4485) | -0.0351 | -0.06039 | -0.17 (-0.39 to 0.06) | 0.142 |
| French Republic | 3964.754 (3560.8704,4412.5785) | 10.761 (9.6648,11.9765) | 5534.3513 (4882.7287,6304.0747) | 16.6731 (14.71,18.9921) | 0.395888 | 0.549401 | 1.38 (1.31 to 1.44) | <0.001 |
| Gabonese Republic | 438.3484 (319.1837,596.1973) | 25.6721 (18.6931,34.9166) | 548.5327 (404.3872,721.8384) | 26.5658 (19.5847,34.9591) | 0.251362 | 0.034812 | 0.17 (0.04 to 0.29) | 0.01 |
| Georgia | 712.1146 (516.0843,968.2137) | 17.1697 (12.4432,23.3444) | 461.316 (335.2209,626.9583) | 21.4766 (15.6062,29.188) | -0.35219 | 0.250843 | 0.80 (0.57 to 1.03) | <0.001 |
| Grand Duchy of Luxembourg | 23.6382 (17.1164,31.3736) | 9.7572 (7.0652,12.9502) | 29.6885 (21.2065,40.2639) | 9.1265 (6.5191,12.3775) | 0.255954 | -0.06464 | -0.17 (-0.28 to -0.05) | 0.004 |
| Greenland | 5.3903 (3.8115,7.5728) | 9.315 (6.5866,13.0865) | 3.4275 (2.3799,4.837) | 9.1705 (6.3676,12.9417) | -0.36414 | -0.01551 | -0.09 (-0.78 to 0.60) | 0.801 |
| Grenada | 11.6096 (8.1679,15.954) | 10.018 (7.0481,13.7669) | 6.7635 (4.8416,9.2827) | 10.4358 (7.4705,14.323) | -0.41742 | 0.041705 | 0.13 (0.08 to 0.18) | <0.001 |
| Guam | 22.0275 (16.2037,29.2848) | 12.1111 (8.9091,16.1013) | 20.0834 (14.8105,27.2985) | 15.8408 (11.6818,21.5317) | -0.08826 | 0.307957 | 0.85 (0.65 to 1.04) | <0.001 |
| Hashemite Kingdom of Jordan | 1272.6456 (970.064,1673.0831) | 19.9147 (15.1798,26.1809) | 1777.1525 (1374.3072,2304.558) | 17.1555 (13.2667,22.2468) | 0.396424 | -0.13855 | -0.48 (-0.57 to -0.40) | <0.001 |
| Hellenic Republic | 749.909 (559.5292,998.3864) | 14.8949 (11.1135,19.8303) | 514.9922 (375.9628,689.7527) | 12.9517 (9.4552,17.3468) | -0.31326 | -0.13046 | -0.44 (-0.51 to -0.36) | <0.001 |
| Hungary | 1220.1895 (884.1566,1625.9086) | 20.192 (14.6312,26.9059) | 783.2702 (568.4328,1052.8932) | 18.3693 (13.3309,24.6926) | -0.35807 | -0.09027 | -0.38 (-0.67 to -0.09) | 0.011 |
| Independent State of Papua New Guinea | 1082.1355 (785.2093,1476.3435) | 15.2989 (11.1011,20.8721) | 2884.4708 (2147.4828,3915.4167) | 17.6039 (13.1061,23.8958) | 1.665536 | 0.150664 | 0.45 (0.40 to 0.50) | <0.001 |
| Independent State of Samoa | 34.7445 (26.6995,45.8258) | 13.42 (10.3126,17.7001) | 44.8416 (31.4886,62.4978) | 15.1038 (10.6062,21.0508) | 0.29061 | 0.125469 | 0.29 (0.18 to 0.41) | <0.001 |
| Ireland | 192.2459 (169.4286,220.8058) | 7.3378 (6.4669,8.4279) | 230.0489 (178.7683,297.3974) | 8.4831 (6.5921,10.9666) | 0.196639 | 0.156082 | 0.42 (0.33 to 0.52) | <0.001 |
| Islamic Republic of Afghanistan | 4523.3801 (3330.4975,6316.8752) | 21.3708 (15.735,29.8441) | 9340.6501 (7012.4178,12272.6055) | 16.0779 (12.0704,21.1247) | 1.064971 | -0.24767 | -0.96 (-1.23 to -0.70) | <0.001 |
| Islamic Republic of Iran | 18758.1265 (14405.5133,24916.1796) | 25.2285 (19.3745,33.5106) | 8543.35 (6407.6067,11099.9861) | 17.2484 (12.9365,22.41) | -0.54455 | -0.31631 | -1.20 (-1.45 to -0.95) | <0.001 |
| Islamic Republic of Mauritania | 692.9004 (510.3059,929.9094) | 16.6924 (12.2936,22.402) | 1022.3825 (741.2791,1394.5678) | 15.7579 (11.4253,21.4944) | 0.475511 | -0.05598 | -0.19 (-0.24 to -0.14) | <0.001 |
| Islamic Republic of Pakistan | 40099.414 (29575.7894,54345.3746) | 19.8795 (14.6623,26.942) | 53016.9945 (38294.8832,72054.0128) | 17.7463 (12.8184,24.1186) | 0.322139 | -0.10731 | -0.32 (-0.49 to -0.14) | <0.001 |
| Jamaica | 380.1366 (277.3214,503.9351) | 13.7367 (10.0213,18.2103) | 219.5577 (161.3403,299.4192) | 13.8482 (10.1762,18.8853) | -0.42242 | 0.008117 | 0.03 (-0.02 to 0.09) | 0.228 |
| Japan | 14557.6112 (10971.9551,19350.3414) | 24.3033 (18.3172,32.3045) | 6951.0742 (5311.8453,9097.5313) | 16.8093 (12.8452,21.9999) | -0.52251 | -0.30835 | -1.16 (-1.26 to -1.07) | <0.001 |
| Kingdom of Bahrain | 102.6551 (77.8595,137.2555) | 16.0759 (12.1929,21.4943) | 125.6444 (95.21,162.1023) | 14.8378 (11.2437,19.1432) | 0.223947 | -0.07702 | -0.28 (-0.45 to -0.10) | 0.002 |
| Kingdom of Belgium | 694.3063 (615.3161,779.2682) | 11.5187 (10.2082,12.9282) | 759.8181 (617.0403,917.9658) | 13.7893 (11.1982,16.6594) | 0.094356 | 0.197123 | 0.56 (0.51 to 0.60) | <0.001 |
| Kingdom of Bhutan | 232.7728 (171.7277,324.2001) | 21.8081 (16.0889,30.3738) | 126.047 (92.2906,174.532) | 21.0577 (15.4183,29.1577) | -0.4585 | -0.03441 | -0.12 (-0.23 to -0.01) | 0.04 |
| Kingdom of Cambodia | 2040.5785 (1499.8992,2748.9969) | 9.9249 (7.2952,13.3705) | 1808.5392 (1328.8051,2432.9594) | 10.5666 (7.7637,14.2148) | -0.11371 | 0.064656 | 0.13 (-0.11 to 0.37) | 0.302 |
| Kingdom of Denmark | 415.6542 (334.427,511.9465) | 13.5717 (10.9195,16.7158) | 342.9878 (254.195,454.798) | 11.1823 (8.2874,14.8275) | -0.17482 | -0.17606 | -0.60 (-0.73 to -0.48) | <0.001 |
| Kingdom of Eswatini | 350.9447 (255.8396,481.8949) | 22.652 (16.5133,31.1042) | 350.6712 (260.9204,467.4215) | 25.286 (18.8143,33.7046) | -0.00078 | 0.116281 | 0.34 (0.20 to 0.49) | <0.001 |
| Kingdom of Lesotho | 594.2356 (431.0797,794.6372) | 23.156 (16.7982,30.9652) | 496.1639 (365.149,676.2899) | 24.4742 (18.0117,33.3593) | -0.16504 | 0.056927 | 0.17 (-0.03 to 0.37) | 0.09 |
| Kingdom of Morocco | 5380.3334 (4123.9927,7016.5421) | 14.2051 (10.8881,18.525) | 3983.2999 (2920.4503,5205.0104) | 13.0131 (9.5409,17.0043) | -0.25966 | -0.08391 | -0.29 (-0.37 to -0.21) | <0.001 |
| Kingdom of Norway | 465.492 (331.7247,649.2097) | 16.2606 (11.5878,22.6782) | 388.1332 (279.4036,536.3612) | 14.5045 (10.4413,20.0438) | -0.16619 | -0.108 | -0.35 (-0.47 to -0.22) | <0.001 |
| Kingdom of Saudi Arabia | 5134.0023 (3876.1369,6828.9235) | 20.9058 (15.7837,27.8075) | 3726.2027 (2777.6934,5013.6257) | 16.731 (12.4721,22.5117) | -0.27421 | -0.1997 | -0.74 (-0.79 to -0.69) | <0.001 |
| Kingdom of Spain | 2141.8204 (1898.9551,2396.443) | 11.1713 (9.9045,12.4993) | 1813.6744 (1516.8007,2136.4151) | 11.2471 (9.4061,13.2485) | -0.15321 | 0.006785 | 0.12 (0.03 to 0.21) | 0.011 |
| Kingdom of Sweden | 1239.6929 (893.0903,1668.1959) | 20.7724 (14.9647,27.9525) | 896.3987 (638.4505,1234.8116) | 16.3369 (11.6358,22.5045) | -0.27692 | -0.21353 | -0.76 (-0.83 to -0.68) | <0.001 |
| Kingdom of Thailand | 5248.6423 (3958.5335,7104.9544) | 10.5285 (7.9406,14.2522) | 2652.1988 (1999.8634,3633.9833) | 10.2841 (7.7546,14.0911) | -0.49469 | -0.02321 | -0.27 (-0.42 to -0.11) | <0.001 |
| Kingdom of the Netherlands | 753.7361 (686.4583,850.9196) | 7.9264 (7.2189,8.9484) | 903.5201 (715.6669,1139.3173) | 10.5591 (8.3637,13.3147) | 0.198722 | 0.332143 | 0.96 (0.70 to 1.23) | <0.001 |
| Kingdom of Tonga | 19.167 (14.433,25.1667) | 12.2823 (9.2487,16.1269) | 20.7902 (15.146,27.6602) | 14.5738 (10.6173,19.3897) | 0.084687 | 0.186569 | 0.48 (0.39 to 0.56) | <0.001 |
| Kyrgyz Republic | 1045.0029 (724.8077,1421.3547) | 16.4488 (11.4088,22.3727) | 1308.2586 (938.1887,1799.1939) | 17.4149 (12.4887,23.95) | 0.251919 | 0.058734 | 0.20 (0.04 to 0.37) | 0.015 |
| Lao People's Democratic Republic | 994.2616 (731.4245,1354.0919) | 11.9257 (8.7731,16.2417) | 887.1103 (636.8703,1226.2483) | 10.5304 (7.56,14.5562) | -0.10777 | -0.117 | -0.37 (-0.54 to -0.21) | <0.001 |
| Lebanese Republic | 726.2513 (543.0298,951.4751) | 17.7528 (13.2741,23.2583) | 576.4094 (431.9998,796.2028) | 15.1832 (11.3793,20.9728) | -0.20632 | -0.14474 | -0.55 (-0.76 to -0.33) | <0.001 |
| Malaysia | 1991.6094 (1477.8557,2657.5724) | 8.335 (6.1849,11.1221) | 2046.2842 (1505.3996,2765.6286) | 8.9159 (6.5592,12.0501) | 0.027453 | 0.069694 | 0.27 (0.20 to 0.33) | <0.001 |
| Mongolia | 585.4621 (423.1291,801.2067) | 16.8958 (12.2111,23.122) | 633.4979 (450.7293,871.6954) | 17.2795 (12.2942,23.7766) | 0.082048 | 0.02271 | 0.07 (-0.08 to 0.23) | 0.346 |
| Montenegro | 99.9348 (73.6666,133.3973) | 20.8894 (15.3986,27.8841) | 53.1093 (38.5219,72.1658) | 15.713 (11.3972,21.3511) | -0.46856 | -0.2478 | -0.94 (-1.01 to -0.86) | <0.001 |
| New Zealand | 618.9898 (459.9644,820.1257) | 21.3504 (15.8653,28.2881) | 516.3559 (377.6076,685.348) | 17.4549 (12.7647,23.1675) | -0.16581 | -0.18246 | -0.60 (-0.81 to -0.38) | <0.001 |
| North Macedonia | 249.195 (184.5201,324.9403) | 15.2754 (11.3109,19.9186) | 120.1129 (87.6113,160.6562) | 13.2245 (9.6461,17.6884) | -0.518 | -0.13426 | -0.45 (-0.55 to -0.34) | <0.001 |
| Northern Mariana Islands | 10.2092 (7.3957,13.5157) | 17.307 (12.5375,22.9123) | 6.1979 (4.49,8.407) | 21.5952 (15.6444,29.2922) | -0.39291 | 0.247773 | 0.71 (0.60 to 0.82) | <0.001 |
| Palestine | 794.9977 (585.0646,1069.9043) | 18.7923 (13.8298,25.2905) | 816.1649 (623.2751,1071.1796) | 14.1435 (10.8009,18.5627) | 0.026625 | -0.24738 | -0.87 (-1.00 to -0.73) | <0.001 |
| People's Democratic Republic of Algeria | 7237.2257 (5472.0759,9686.8126) | 19.5529 (14.784,26.171) | 7164.5377 (5311.2132,9668.1963) | 16.4205 (12.1728,22.1587) | -0.01004 | -0.1602 | -0.56 (-0.57 to -0.55) | <0.001 |
| People's Republic of Bangladesh | 46585.3033 (34725.74,62482.6389) | 22.8525 (17.0348,30.651) | 32661.9272 (23238.5763,45245.3862) | 24.3365 (17.3152,33.7125) | -0.29888 | 0.064938 | 0.23 (0.16 to 0.29) | <0.001 |
| People's Republic of China | 155513.6603 (115458.9588,206204.2635) | 14.0665 (10.4435,18.6516) | 58245.5151 (42177.6635,78934.0862) | 10.9894 (7.9578,14.8928) | -0.62546 | -0.21875 | -0.74 (-0.93 to -0.55) | <0.001 |
| Plurinational State of Bolivia | 1139.5806 (876.3049,1499.994) | 10.4145 (8.0085,13.7083) | 1376.7881 (1022.3434,1876.9334) | 11.7588 (8.7315,16.0304) | 0.208153 | 0.12908 | 0.48 (0.35 to 0.61) | <0.001 |
| Portuguese Republic | 652.1793 (496.3128,868.2978) | 11.7768 (8.9623,15.6794) | 379.5161 (284.2371,498.7789) | 9.6144 (7.2007,12.6358) | -0.41808 | -0.18362 | -0.62 (-0.67 to -0.57) | <0.001 |
| Principality of Andorra | 4.2915 (3.1736,5.6845) | 16.9172 (12.5106,22.4085) | 2.9773 (2.1813,4.0594) | 13.4459 (9.8508,18.3326) | -0.30623 | -0.20519 | -0.68 (-1.13 to -0.23) | 0.003 |
| Principality of Monaco | 1.7006 (1.2391,2.321) | 13.1465 (9.5784,17.9424) | 1.4767 (1.0533,2.0068) | 9.6225 (6.8633,13.0767) | -0.13166 | -0.26806 | -0.99 (-1.09 to -0.89) | <0.001 |
| Puerto Rico | 434.0001 (321.9024,579.1921) | 13.7872 (10.2261,18.3996) | 120.6654 (89.0914,163.4695) | 13.6693 (10.0925,18.5182) | -0.72197 | -0.00855 | -0.01 (-0.17 to 0.15) | 0.924 |
| Republic of Albania | 777.1572 (570.5679,1045.193) | 19.9931 (14.6784,26.8886) | 265.7082 (189.7882,366.7656) | 19.9013 (14.215,27.4704) | -0.6581 | -0.00459 | -0.03 (-0.12 to 0.06) | 0.481 |
| Republic of Angola | 6236.0568 (4702.8687,8222.1081) | 25.8911 (19.5255,34.1368) | 15631.0015 (11182.0621,21961.5329) | 26.9638 (19.2893,37.884) | 1.506552 | 0.041431 | 0.11 (0.07 to 0.15) | <0.001 |
| Republic of Armenia | 635.8568 (467.4562,853.2446) | 17.5122 (12.8742,23.4992) | 317.7762 (229.2156,442.2545) | 19.1694 (13.8271,26.6784) | -0.50024 | 0.094631 | 0.30 (0.25 to 0.34) | <0.001 |
| Republic of Austria | 556.6218 (479.953,644.3492) | 12.4644 (10.7475,14.4289) | 565.871 (450.8024,712.4558) | 13.8281 (11.0161,17.4101) | 0.016617 | 0.109408 | 0.25 (-0.19 to 0.69) | 0.272 |
| Republic of Azerbaijan | 1657.2046 (1201.5994,2235.2056) | 18.6188 (13.5001,25.1127) | 1294.1023 (943.8948,1750.2912) | 20.0886 (14.6523,27.1702) | -0.21911 | 0.078942 | 0.23 (0.13 to 0.33) | <0.001 |
| Republic of Belarus | 1085.7574 (793.337,1458.0893) | 15.8868 (11.6081,21.3347) | 524.2456 (374.1644,717.9321) | 13.1457 (9.3823,18.0024) | -0.51716 | -0.17254 | -0.60 (-0.73 to -0.47) | <0.001 |
| Republic of Benin | 2005.1618 (1492.3465,2589.2048) | 17.5739 (13.0794,22.6927) | 4461.0092 (3234.8167,5986.5241) | 17.7826 (12.8947,23.8637) | 1.224763 | 0.011876 | 0.04 (-0.03 to 0.11) | 0.24 |
| Republic of Botswana | 500.9982 (362.5031,696.4894) | 22.5169 (16.2924,31.3031) | 585.622 (425.5965,804.6721) | 25.1584 (18.2837,34.5688) | 0.16891 | 0.117312 | 0.36 (0.11 to 0.60) | 0.004 |
| Republic of Bulgaria | 825.0461 (597.2695,1115.8147) | 17.2492 (12.4871,23.3283) | 373.3836 (278.2889,514.8402) | 13.3805 (9.9727,18.4498) | -0.54744 | -0.22428 | -0.82 (-0.90 to -0.73) | <0.001 |
| Republic of Burundi | 2769.1082 (2059.6079,3791.41) | 22.1909 (16.5052,30.3834) | 4383.8733 (3185.3905,5721.3128) | 19.5554 (14.2092,25.5213) | 0.583135 | -0.11876 | -0.41 (-0.72 to -0.10) | 0.011 |
| Republic of Cabo Verde | 106.1348 (77.4617,143.4879) | 17.6023 (12.8469,23.7972) | 69.8329 (51.8498,94.0233) | 17.1508 (12.7342,23.0919) | -0.34204 | -0.02565 | -0.11 (-0.16 to -0.07) | <0.001 |
| Republic of Cameroon | 3932.5294 (2917.9045,5302.3543) | 17.1717 (12.7413,23.1532) | 8878.4953 (6701.0268,11538.9877) | 17.8511 (13.4731,23.2003) | 1.257706 | 0.039565 | 0.10 (0.02 to 0.18) | 0.012 |
| Republic of Chad | 2726.5398 (1990.5607,3683.2268) | 17.755 (12.9624,23.9849) | 6943.1972 (5178.8847,9255.3067) | 16.7709 (12.5093,22.3557) | 1.546523 | -0.05543 | -0.19 (-0.21 to -0.16) | <0.001 |
| Republic of Chile | 2229.231 (1700.4321,2885.8554) | 15.2079 (11.6004,19.6874) | 1414.5682 (1100.6635,1801.6489) | 14.5065 (11.2874,18.4761) | -0.36545 | -0.04612 | -0.10 (-0.22 to 0.01) | 0.081 |
| Republic of Colombia | 5215.5701 (3821.7543,7013.7687) | 11.7942 (8.6423,15.8606) | 4241.9188 (3134.4967,5726.8778) | 13.1017 (9.6813,17.6882) | -0.18668 | 0.11086 | 0.33 (0.24 to 0.43) | <0.001 |
| Republic of Costa Rica | 480.8135 (361.7154,632.34) | 12.2892 (9.2451,16.1621) | 310.5043 (229.8024,420.748) | 11.8305 (8.7557,16.0309) | -0.35421 | -0.03733 | -0.12 (-0.33 to 0.08) | 0.245 |
| Republic of C么te d'Ivoire | 5062.1314 (3652.4905,6993.3543) | 19.0442 (13.741,26.3096) | 8314.0342 (6129.2725,10998.7063) | 18.3205 (13.5062,24.2363) | 0.642398 | -0.038 | -0.13 (-0.17 to -0.08) | <0.001 |
| Republic of Croatia | 227.6134 (186.1284,278.404) | 8.5221 (6.9689,10.4238) | 221.736 (168.4412,284.0133) | 13.1142 (9.9622,16.7975) | -0.02582 | 0.538846 | 1.47 (1.33 to 1.60) | <0.001 |
| Republic of Cuba | 1204.3101 (923.3402,1585.0953) | 14.0596 (10.7794,18.505) | 542.0654 (396.3739,736.44) | 11.2567 (8.2312,15.2931) | -0.5499 | -0.19936 | -0.77 (-1.14 to -0.40) | <0.001 |
| Republic of Cyprus | 99.6409 (73.0846,131.8536) | 15.0866 (11.0657,19.9639) | 79.2746 (57.6651,106.4145) | 10.9058 (7.933,14.6394) | -0.2044 | -0.27712 | -1.04 (-1.34 to -0.74) | <0.001 |
| Republic of Djibouti | 136.4799 (100.5673,183.7564) | 18.2409 (13.4411,24.5595) | 243.8943 (176.0595,351.558) | 16.8397 (12.156,24.2733) | 0.787035 | -0.07682 | -0.12 (-0.31 to 0.06) | 0.197 |
| Republic of Ecuador | 1560.2419 (1202.0567,1993.4333) | 10.7845 (8.3087,13.7787) | 1952.2478 (1530.8109,2457.2295) | 12.6727 (9.937,15.9507) | 0.251247 | 0.175085 | 0.50 (0.37 to 0.62) | <0.001 |
| Republic of El Salvador | 839.427 (603.8928,1144.9935) | 10.0456 (7.2269,13.7024) | 701.285 (510.248,949.7691) | 12.5442 (9.127,16.9889) | -0.16457 | 0.248726 | 0.71 (0.63 to 0.79) | <0.001 |
| Republic of Equatorial Guinea | 234.1239 (171.6672,312.7516) | 23.7727 (17.4309,31.7565) | 465.6344 (343.4074,633.4439) | 25.9023 (19.1031,35.2372) | 0.988838 | 0.089582 | 0.28 (0.22 to 0.33) | <0.001 |
| Republic of Estonia | 155.1263 (115.5148,208.7206) | 15.0063 (11.1744,20.1908) | 87.243 (62.0966,119.2573) | 13.973 (9.9455,19.1004) | -0.4376 | -0.06886 | -0.23 (-0.33 to -0.14) | <0.001 |
| Republic of Fiji | 149.486 (112.2599,201.2952) | 16.5315 (12.4147,22.261) | 165.0524 (122.1564,225.7437) | 19.0192 (14.0762,26.0127) | 0.104133 | 0.150482 | 0.46 (0.39 to 0.54) | <0.001 |
| Republic of Finland | 319.8055 (258.5465,396.6018) | 10.1292 (8.1889,12.5615) | 235.5178 (177.3184,319.2313) | 10.1294 (7.6263,13.7299) | -0.26356 | 1.97E-05 | -0.03 (-0.31 to 0.25) | 0.843 |
| Republic of Ghana | 4710.9097 (3418.3399,6424.7349) | 16.2725 (11.8077,22.1924) | 8191.5129 (5979.924,11008.1867) | 17.6349 (12.8737,23.6987) | 0.738839 | 0.083724 | 0.29 (0.18 to 0.40) | <0.001 |
| Republic of Guatemala | 1760.8149 (1286.7104,2382.034) | 10.6005 (7.7463,14.3403) | 1995.9544 (1473.7464,2734.4505) | 14.0067 (10.342,19.1891) | 0.13354 | 0.321324 | 0.88 (0.74 to 1.01) | <0.001 |
| Republic of Guinea | 2665.2838 (1981.8724,3560.1757) | 19.1082 (14.2086,25.5239) | 4121.4268 (3082.3509,5414.4088) | 17.2957 (12.9352,22.7218) | 0.546337 | -0.09485 | -0.35 (-0.55 to -0.16) | <0.001 |
| Republic of Guinea-Bissau | 393.2097 (291.4839,537.049) | 17.8482 (13.2308,24.3772) | 526.2938 (383.8786,708.0924) | 15.3051 (11.1636,20.592) | 0.338456 | -0.14248 | -0.49 (-0.58 to -0.41) | <0.001 |
| Republic of Guyana | 167.569 (124.6895,225.0269) | 13.3892 (9.963,17.9802) | 95.1002 (69.7046,124.5535) | 13.2644 (9.7223,17.3725) | -0.43247 | -0.00932 | -0.00 (-0.05 to 0.05) | 0.98 |
| Republic of Haiti | 1869.0372 (1390.0074,2511.1318) | 15.4302 (11.4755,20.7311) | 2449.1556 (1746.9652,3274.8036) | 14.9363 (10.6539,19.9715) | 0.310384 | -0.03201 | -0.06 (-0.18 to 0.06) | 0.348 |
| Republic of Honduras | 1044.0482 (752.2396,1392.5071) | 12.1548 (8.7576,16.2116) | 1162.5147 (836.7711,1616.1939) | 10.8892 (7.838,15.1388) | 0.113468 | -0.10412 | -0.40 (-0.56 to -0.24) | <0.001 |
| Republic of Iceland | 35.8652 (27.0328,48.7825) | 16.1093 (12.1421,21.9112) | 30.45 (22.3849,41.5424) | 13.7046 (10.0747,18.6969) | -0.15099 | -0.14927 | -0.52 (-0.65 to -0.39) | <0.001 |
| Republic of India | 273634.4746 (203906.8149,369202.3571) | 22.7253 (16.9344,30.6622) | 246828.0718 (181887.3566,334527.8952) | 23.5174 (17.3299,31.8733) | -0.09796 | 0.034855 | 0.12 (0.04 to 0.20) | 0.004 |
| Republic of Indonesia | 24280.9801 (18068.1085,32667.2897) | 10.7835 (8.0243,14.508) | 20648.1415 (15108.9899,27878.1144) | 9.7479 (7.1329,13.1612) | -0.14962 | -0.09604 | -0.30 (-0.50 to -0.11) | 0.002 |
| Republic of Iraq | 7065.7549 (5322.718,9196.4421) | 21.292 (16.0395,27.7126) | 7129.5102 (5303.0374,9362.9845) | 17.9139 (13.3247,23.5259) | 0.009023 | -0.15866 | -0.52 (-0.93 to -0.10) | 0.016 |
| Republic of Italy | 4301.1248 (3183.6929,5749.6504) | 15.9838 (11.8312,21.3668) | 2710.5322 (2098.2426,3435.8858) | 13.9676 (10.8124,17.7054) | -0.36981 | -0.12614 | -0.42 (-0.49 to -0.35) | <0.001 |
| Republic of Kazakhstan | 3239.4969 (2317.4992,4302.5568) | 18.2513 (13.0568,24.2406) | 3634.9811 (2607.6843,4893.8068) | 18.5231 (13.2882,24.9378) | 0.122082 | 0.014892 | -0.01 (-0.16 to 0.14) | 0.913 |
| Republic of Kenya | 11704.4621 (8590.9071,15658.2038) | 24.7964 (18.2002,33.1725) | 13047.6823 (9333.0627,17390.0211) | 23.0319 (16.4748,30.6971) | 0.114761 | -0.07116 | -0.15 (-0.39 to 0.08) | 0.206 |
| Republic of Kiribati | 18.3114 (13.5993,24.7882) | 14.1083 (10.4778,19.0984) | 20.6404 (15.2628,27.746) | 14.6873 (10.8608,19.7436) | 0.127189 | 0.04104 | 0.15 (0.10 to 0.19) | <0.001 |
| Republic of Korea | 5344.4352 (3976.7224,7150.4429) | 16.5475 (12.3128,22.1393) | 2005.7502 (1483.2565,2736.8531) | 15.6118 (11.545,21.3024) | -0.6247 | -0.05655 | -0.17 (-0.28 to -0.06) | 0.002 |
| Republic of Latvia | 280.3234 (204.0716,378.0364) | 15.8533 (11.541,21.3793) | 118.0524 (85.9619,161.672) | 14.5666 (10.6069,19.9489) | -0.57887 | -0.08116 | -0.27 (-0.37 to -0.18) | <0.001 |
| Republic of Liberia | 1155.1448 (860.8982,1518.0756) | 21.3886 (15.9403,28.1086) | 1340.5702 (987.9405,1796.0294) | 17.1823 (12.6626,23.0199) | 0.160521 | -0.19666 | -0.95 (-1.33 to -0.57) | <0.001 |
| Republic of Lithuania | 462.8981 (346.8217,620.6039) | 17.0741 (12.7926,22.8911) | 139.225 (103.7077,187.4022) | 12.1642 (9.061,16.3734) | -0.69923 | -0.28756 | -1.07 (-1.23 to -0.92) | <0.001 |
| Republic of Madagascar | 5784.4196 (4216.6109,8074.8975) | 23.1212 (16.8544,32.2766) | 7977.7863 (5823.2562,10588.7864) | 19.6954 (14.3763,26.1413) | 0.379185 | -0.14817 | -0.46 (-0.59 to -0.33) | <0.001 |
| Republic of Malawi | 5867.7327 (4346.6387,8052.0799) | 24.4684 (18.1254,33.5771) | 5515.4806 (4067.6642,7228.1958) | 20.2143 (14.9081,26.4915) | -0.06003 | -0.17386 | -0.55 (-0.75 to -0.34) | <0.001 |
| Republic of Maldives | 44.9358 (33.6106,61.6172) | 10.5513 (7.8921,14.4682) | 31.3684 (22.7526,41.965) | 10.8538 (7.8727,14.5204) | -0.30193 | 0.028669 | 0.14 (0.02 to 0.26) | 0.024 |
| Republic of Mali | 3994.282 (2867.8224,5400.3673) | 18.6419 (13.3845,25.2043) | 9137.9585 (6662.7501,12328.8533) | 17.905 (13.055,24.1572) | 1.28776 | -0.03953 | -0.11 (-0.29 to 0.06) | 0.206 |
| Republic of Malta | 28.7589 (23.8631,35.1067) | 10.7519 (8.9215,13.1251) | 27.3054 (21.2442,34.3068) | 13.0479 (10.1515,16.3935) | -0.05054 | 0.213544 | 0.66 (0.56 to 0.77) | <0.001 |
| Republic of Mauritius | 105.7249 (77.6553,142.0345) | 9.5192 (6.9919,12.7885) | 62.1565 (46.0904,82.7386) | 10.1925 (7.558,13.5676) | -0.41209 | 0.070731 | 0.21 (0.18 to 0.25) | <0.001 |
| Republic of Moldova | 782.3436 (587.8098,1024.3598) | 20.534 (15.4281,26.8862) | 225.6716 (165.6047,308.5065) | 16.6234 (12.1987,22.7252) | -0.71154 | -0.19045 | -0.70 (-0.75 to -0.65) | <0.001 |
| Republic of Mozambique | 7036.3571 (5085.311,9695.4903) | 24.0195 (17.3594,33.0968) | 11124.1244 (8249.9889,14968.901) | 20.9859 (15.5638,28.2392) | 0.580949 | -0.1263 | -0.44 (-0.52 to -0.35) | <0.001 |
| Republic of Namibia | 553.7394 (408.3901,742.3775) | 22.516 (16.6059,30.1864) | 663.8759 (491.7813,900.6067) | 24.1132 (17.8624,32.7117) | 0.198896 | 0.070936 | 0.20 (0.08 to 0.31) | <0.001 |
| Republic of Nauru | 2.1945 (1.6541,2.8835) | 12.9925 (9.7933,17.0722) | 2.134 (1.6013,2.8772) | 15.338 (11.5093,20.6798) | -0.02757 | 0.180527 | 0.45 (0.25 to 0.66) | <0.001 |
| Republic of Nicaragua | 681.7158 (483.4834,950.5865) | 9.9808 (7.0786,13.9173) | 647.8507 (462.3572,899.3009) | 10.5299 (7.515,14.6169) | -0.04968 | 0.055016 | 0.16 (0.10 to 0.23) | <0.001 |
| Republic of Niue | 0.3033 (0.2305,0.399) | 13.7198 (10.4285,18.0498) | 0.2008 (0.147,0.2652) | 18.2217 (13.3352,24.063) | -0.33795 | 0.328132 | 0.92 (0.79 to 1.04) | <0.001 |
| Republic of Palau | 2.0917 (1.5646,2.7431) | 13.9172 (10.4103,18.2507) | 1.3855 (1.0167,1.8776) | 16.0936 (11.8097,21.8086) | -0.33762 | 0.156382 | 0.35 (0.21 to 0.49) | <0.001 |
| Republic of Panama | 394.8612 (297.0628,526.7159) | 13.9234 (10.4748,18.5727) | 451.4064 (333.0706,598.3397) | 13.3165 (9.8256,17.651) | 0.143203 | -0.04359 | -0.23 (-0.36 to -0.09) | <0.001 |
| Republic of Paraguay | 550.6291 (383.3327,776.4545) | 8.8666 (6.1727,12.503) | 501.0251 (340.8594,690.0076) | 8.0847 (5.5002,11.1342) | -0.09009 | -0.08818 | -0.30 (-0.35 to -0.24) | <0.001 |
| Republic of Peru | 3331.5426 (2389.4777,4504.267) | 10.7849 (7.7353,14.5813) | 3989.1823 (2907.9669,5455.3846) | 12.3384 (8.9943,16.8734) | 0.197398 | 0.144044 | 0.47 (0.33 to 0.61) | <0.001 |
| Republic of Poland | 4143.6405 (3013.2221,5455.4112) | 15.7755 (11.4718,20.7696) | 1811.9654 (1382.0877,2377.201) | 11.0306 (8.4137,14.4716) | -0.56271 | -0.30078 | -1.18 (-1.34 to -1.01) | <0.001 |
| Republic of Rwanda | 3145.8238 (2339.2042,4139.1485) | 20.6195 (15.3325,27.1304) | 3445.2776 (2534.9099,4763.5601) | 19.4556 (14.3148,26.9001) | 0.095191 | -0.05645 | -0.18 (-0.44 to 0.09) | 0.199 |
| Republic of San Marino | 1.3157 (0.9687,1.8013) | 11.201 (8.247,15.335) | 1.2441 (0.9046,1.6887) | 11.2408 (8.1738,15.2583) | -0.05442 | 0.003553 | 0.01 (-0.07 to 0.09) | 0.794 |
| Republic of Senegal | 3099.9763 (2332.2764,4208.6971) | 18.7053 (14.073,25.3953) | 3955.2081 (2941.703,5350.6152) | 17.1512 (12.7563,23.2022) | 0.275883 | -0.08308 | -0.30 (-0.56 to -0.03) | 0.028 |
| Republic of Serbia | 1152.4224 (872.0204,1499.3783) | 17.4261 (13.1861,22.6726) | 371.884 (270.1777,500.8147) | 11.5195 (8.369,15.5132) | -0.6773 | -0.33895 | -1.30 (-1.38 to -1.22) | <0.001 |
| Republic of Seychelles | 7.8256 (5.7982,10.6649) | 9.8958 (7.3322,13.4863) | 9.0691 (6.617,12.3344) | 11.9504 (8.7192,16.2531) | 0.158902 | 0.207623 | 0.64 (0.49 to 0.79) | <0.001 |
| Republic of Sierra Leone | 2071.906 (1541.4782,2864.8049) | 21.2845 (15.8354,29.4299) | 2828.2314 (2067.0783,3802.9201) | 19.5356 (14.278,26.2681) | 0.365038 | -0.08217 | -0.26 (-0.51 to -0.00) | 0.046 |
| Republic of Singapore | 656.2497 (497.1237,849.2803) | 27.0926 (20.5232,35.0617) | 802.5503 (561.8092,1128.9609) | 30.3261 (21.2292,42.6602) | 0.222934 | 0.11935 | 0.23 (-0.13 to 0.59) | 0.218 |
| Republic of Slovenia | 158.284 (119.908,209.2654) | 14.7989 (11.2109,19.5655) | 105.4738 (75.5675,147.5539) | 11.8005 (8.4546,16.5085) | -0.33364 | -0.20261 | -0.72 (-0.84 to -0.61) | <0.001 |
| Republic of South Africa | 14472.284 (10695.199,19331.7094) | 28.7661 (21.2585,38.425) | 14648.8878 (10705.4435,19805.8662) | 30.9929 (22.6497,41.9036) | 0.012203 | 0.077411 | 0.24 (0.16 to 0.32) | <0.001 |
| Republic of South Sudan | 2835.9561 (2069.7942,3893.0075) | 23.108 (16.8652,31.7211) | 3903.9652 (2811.6124,5196.4793) | 21.2965 (15.3376,28.3472) | 0.376596 | -0.07839 | -0.24 (-0.48 to -0.00) | 0.047 |
| Republic of Sudan | 8685.8078 (6403.6342,11464.6774) | 20.7105 (15.2688,27.3364) | 9049.3799 (6678.3505,11960.8935) | 16.422 (12.1193,21.7055) | 0.041858 | -0.20707 | -0.73 (-0.80 to -0.66) | <0.001 |
| Republic of Suriname | 57.9986 (42.9276,77.43) | 13.1953 (9.7665,17.6162) | 56.2029 (41.6604,74.3913) | 13.1733 (9.7647,17.4364) | -0.03096 | -0.00167 | 0.01 (-0.13 to 0.15) | 0.852 |
| Republic of Tajikistan | 1647.6485 (1210.4014,2243.1487) | 16.4687 (12.0983,22.4208) | 2475.9466 (1774.0993,3368.444) | 18.2105 (13.0484,24.7748) | 0.502715 | 0.105764 | 0.33 (0.23 to 0.42) | <0.001 |
| Republic of the Congo | 996.7008 (728.6947,1315.9872) | 22.7767 (16.6522,30.0731) | 1558.2392 (1134.7122,2086.173) | 25.5168 (18.5814,34.1619) | 0.563397 | 0.120303 | 0.34 (0.24 to 0.43) | <0.001 |
| Republic of the Gambia | 346.964 (255.6688,459.3907) | 16.0118 (11.7987,21.2001) | 570.5484 (413.7881,756.4238) | 15.3969 (11.1666,20.413) | 0.644402 | -0.0384 | -0.15 (-0.17 to -0.13) | <0.001 |
| Republic of the Marshall Islands | 9.981 (7.4513,13.2805) | 13.8983 (10.3757,18.4928) | 9.1045 (6.9346,12.4671) | 16.6192 (12.6583,22.7571) | -0.08782 | 0.195772 | 0.53 (0.41 to 0.65) | <0.001 |
| Republic of the Niger | 3821.595 (2818.7562,5192.802) | 17.8715 (13.1818,24.2839) | 9077.999 (6843.9568,12060.0672) | 16.0148 (12.0737,21.2756) | 1.375448 | -0.10389 | -0.38 (-0.47 to -0.28) | <0.001 |
| Republic of the Philippines | 10147.7248 (7599.2187,13520.4995) | 10.4641 (7.8361,13.942) | 10082.9549 (7484.8876,13518.1823) | 9.243 (6.8613,12.392) | -0.00638 | -0.11669 | -0.40 (-0.44 to -0.36) | <0.001 |
| Republic of the Union of Myanmar | 5847.3761 (4235.267,8230.9593) | 10.9099 (7.9021,15.3572) | 5814.1621 (4146.1324,7883.9334) | 11.3032 (8.0604,15.327) | -0.00568 | 0.03605 | 0.01 (-0.25 to 0.27) | 0.958 |
| Republic of Trinidad and Tobago | 157.3846 (115.1655,211.0274) | 13.7164 (10.0369,18.3915) | 100.4922 (74.5814,133.928) | 14.0828 (10.4517,18.7684) | -0.36149 | 0.026713 | 0.09 (-0.01 to 0.19) | 0.089 |
| Republic of Tunisia | 2128.4232 (1605.8755,2752.7147) | 20.1892 (15.2325,26.1109) | 1184.6439 (888.1655,1570.8581) | 14.7782 (11.0797,19.5962) | -0.44342 | -0.26801 | -1.00 (-1.04 to -0.97) | <0.001 |
| Republic of Turkey | 12896.7845 (9736.3115,17588.6726) | 18.1455 (13.6987,24.7468) | 7564.8711 (5702.2562,9921.9547) | 15.6063 (11.7637,20.4689) | -0.41343 | -0.13994 | -0.52 (-0.61 to -0.42) | <0.001 |
| Republic of Uganda | 10312.0067 (7603.0419,13956.6212) | 23.3377 (17.2069,31.586) | 16948.1361 (12282.9045,23131.4105) | 22.4054 (16.2379,30.5796) | 0.643534 | -0.03995 | -0.11 (-0.18 to -0.05) | <0.001 |
| Republic of Uzbekistan | 5357.8194 (3796.8577,7422.0544) | 15.7105 (11.1334,21.7634) | 6341.6053 (4514.6083,8768.95) | 16.5688 (11.7954,22.9108) | 0.183617 | 0.054632 | 0.19 (0.09 to 0.28) | <0.001 |
| Republic of Vanuatu | 36.559 (26.5435,49.6687) | 12.5328 (9.0994,17.0269) | 58.5296 (42.9223,76.0519) | 13.9349 (10.219,18.1066) | 0.600963 | 0.111874 | 0.34 (0.18 to 0.49) | <0.001 |
| Republic of Yemen | 5427.2932 (4084.7036,7370.2412) | 17.2104 (12.9529,23.3716) | 6881.6411 (5106.6692,9327.2544) | 14.7719 (10.9618,20.0216) | 0.267969 | -0.14169 | -0.51 (-0.57 to -0.44) | <0.001 |
| Republic of Zambia | 4148.1133 (3025.7216,5734.81) | 22.658 (16.5272,31.3249) | 6319.6695 (4575.2363,8496.9156) | 21.8178 (15.7954,29.3345) | 0.523505 | -0.03708 | -0.12 (-0.16 to -0.09) | <0.001 |
| Republic of Zimbabwe | 4840.6672 (3485.1324,6716.485) | 26.0421 (18.7495,36.1337) | 5464.8137 (4020.373,7435.5294) | 24.3914 (17.9443,33.1874) | 0.128938 | -0.06339 | -0.20 (-0.52 to 0.11) | 0.208 |
| Romania | 1950.131 (1463.0749,2601.9771) | 13.4307 (10.0763,17.9201) | 1029.668 (765.4204,1397.0511) | 12.0097 (8.9276,16.2947) | -0.472 | -0.1058 | -0.35 (-0.39 to -0.32) | <0.001 |
| Russian Federation | 26945.8786 (20072.1256,36063.8652) | 28.3172 (21.0936,37.8992) | 15748.2425 (11320.6046,21133.8982) | 24.1913 (17.3899,32.4643) | -0.41556 | -0.1457 | -0.49 (-0.60 to -0.38) | <0.001 |
| Saint Kitts and Nevis | 4.6694 (3.3587,6.4426) | 10.4503 (7.5169,14.4187) | 2.9017 (2.0507,4.0173) | 10.4067 (7.3547,14.4078) | -0.37857 | -0.00417 | 0.08 (-0.14 to 0.29) | 0.476 |
| Saint Lucia | 24.6967 (18.4775,32.6196) | 14.4222 (10.7903,19.0489) | 12.0539 (8.9007,15.8361) | 14.9035 (11.0049,19.5799) | -0.51192 | 0.033372 | 0.15 (0.00 to 0.29) | 0.05 |
| Saint Vincent and the Grenadines | 19.4185 (14.277,25.4683) | 15.7096 (11.5501,20.6039) | 9.0198 (6.7256,11.7438) | 14.4047 (10.7408,18.7549) | -0.5355 | -0.08306 | -0.34 (-0.73 to 0.06) | 0.093 |
| Slovak Republic | 756.0983 (566.841,986.4245) | 19.8193 (14.8584,25.8568) | 470.983 (351.4016,627.4901) | 17.5874 (13.122,23.4317) | -0.37709 | -0.11261 | -0.29 (-0.51 to -0.08) | 0.007 |
| Socialist Republic of Viet Nam | 8996.1505 (6682.4286,12167.1038) | 9.6837 (7.1931,13.0969) | 7703.9403 (5416.2433,10734.9963) | 10.3434 (7.2719,14.4129) | -0.14364 | 0.068125 | 0.20 (0.17 to 0.23) | <0.001 |
| Solomon Islands | 82.4148 (62.4233,107.6247) | 12.5912 (9.537,16.4428) | 147.8227 (109.3349,199.2679) | 15.1222 (11.1849,20.385) | 0.793643 | 0.201013 | 0.43 (0.38 to 0.49) | <0.001 |
| State of Eritrea | 1330.5364 (961.8773,1754.5996) | 18.7403 (13.5478,24.7131) | 1742.3875 (1282.8801,2368.8133) | 18.6149 (13.7057,25.3073) | 0.309538 | -0.00669 | -0.08 (-0.41 to 0.26) | 0.65 |
| State of Israel | 908.2953 (671.5588,1243.36) | 17.9581 (13.2775,24.5827) | 1190.1574 (875.5953,1625.3519) | 13.413 (9.8679,18.3176) | 0.31032 | -0.25309 | -0.94 (-1.02 to -0.87) | <0.001 |
| State of Kuwait | 354.1239 (270.2803,467.8772) | 21.9804 (16.7763,29.0411) | 548.764 (416.4247,728.3562) | 22.898 (17.3759,30.3917) | 0.549638 | 0.041746 | 0.17 (-0.08 to 0.42) | 0.188 |
| State of Libya | 1187.6884 (903.8315,1573.6543) | 18.9941 (14.4545,25.1666) | 522.7319 (399.8815,683.7398) | 13.5745 (10.3842,17.7556) | -0.55987 | -0.28533 | -1.06 (-1.35 to -0.77) | <0.001 |
| State of Qatar | 109.1877 (81.5897,150.6257) | 19.9525 (14.9094,27.5248) | 314.9061 (235.8315,428.4082) | 16.9763 (12.7135,23.0951) | 1.88408 | -0.14916 | -0.53 (-0.66 to -0.39) | <0.001 |
| Sultanate of Oman | 486.8295 (371.0251,655.0687) | 14.1071 (10.7514,18.9823) | 515.2487 (389.5267,685.1677) | 13.5539 (10.2467,18.0237) | 0.058376 | -0.03921 | -0.15 (-0.26 to -0.04) | 0.007 |
| Swiss Confederation | 702.7882 (612.0816,817.1402) | 17.3676 (15.126,20.1935) | 610.1648 (477.4785,763.3206) | 14.4212 (11.2851,18.041) | -0.13179 | -0.16965 | -0.64 (-0.73 to -0.55) | <0.001 |
| Syrian Arab Republic | 3215.6646 (2441.9628,4268.6548) | 14.5846 (11.0755,19.3604) | 1134.8994 (854.1683,1512.424) | 12.0203 (9.0469,16.0188) | -0.64707 | -0.17582 | -0.68 (-0.97 to -0.39) | <0.001 |
| Taiwan (Province of China) | 2436.4223 (1823.3139,3291.1201) | 15.5782 (11.6581,21.0431) | 1180.3035 (883.8135,1587.6694) | 15.5906 (11.6743,20.9715) | -0.51556 | 0.000796 | -0.12 (-0.21 to -0.02) | 0.016 |
| Togolese Republic | 1329.0578 (964.5188,1809.7847) | 17.2739 (12.5359,23.5219) | 1796.3464 (1298.2926,2420.9449) | 15.4352 (11.1557,20.8022) | 0.351594 | -0.10644 | -0.35 (-0.79 to 0.09) | 0.116 |
| Tokelau | 0.2034 (0.1509,0.2708) | 11.5904 (8.598,15.4324) | 0.1076 (0.0772,0.1475) | 12.6883 (9.1031,17.3966) | -0.47099 | 0.094725 | 0.28 (-0.24 to 0.79) | 0.291 |
| Turkmenistan | 1103.691 (785.8446,1476.1629) | 18.3302 (13.0514,24.5163) | 1206.1478 (851.6753,1617.5796) | 22.7863 (16.0897,30.5589) | 0.092831 | 0.243102 | 0.66 (0.62 to 0.71) | <0.001 |
| Tuvalu | 2.7698 (2.0595,3.7253) | 16.8455 (12.5256,22.6567) | 1.8553 (1.3681,2.524) | 14.5148 (10.7028,19.7455) | -0.33017 | -0.13836 | -0.50 (-0.56 to -0.43) | <0.001 |
| Ukraine | 6064.6774 (4531.5539,8121.4183) | 18.8247 (14.0659,25.2088) | 1888.5197 (1369.4674,2659.4031) | 14.2952 (10.3662,20.1304) | -0.6886 | -0.24061 | -0.91 (-1.16 to -0.66) | <0.001 |
| Union of the Comoros | 205.3765 (149.3576,275.7563) | 21.7027 (15.783,29.1399) | 157.4397 (114.7107,215.2687) | 19.3116 (14.0704,26.4049) | -0.23341 | -0.11018 | -0.30 (-0.61 to 0.01) | 0.06 |
| United Arab Emirates | 399.8817 (306.9307,535.7966) | 17.2123 (13.2114,23.0626) | 498.0572 (367.3465,654.1683) | 13.5915 (10.0246,17.8517) | 0.245511 | -0.21036 | -0.80 (-0.94 to -0.66) | <0.001 |
| United Kingdom of Great Britain and Northern Ireland | 6643.6056 (4851.6326,8852.3389) | 17.3666 (12.6823,23.1403) | 4823.4285 (3590.6289,6383.1136) | 14.4231 (10.7367,19.0868) | -0.27397 | -0.16949 | -0.60 (-0.69 to -0.50) | <0.001 |
| United Mexican States | 26984.1053 (19569.5717,35793.1524) | 22.1508 (16.0643,29.382) | 16794.0547 (12652.1501,21995.8876) | 18.5318 (13.9613,24.2718) | -0.37763 | -0.16338 | -0.57 (-0.63 to -0.51) | <0.001 |
| United Republic of Tanzania | 15317.732 (11006.83,21634.8049) | 26.5665 (19.0899,37.5227) | 19258.4494 (14534.4946,25971.3053) | 21.3209 (16.0911,28.7527) | 0.257265 | -0.19745 | -0.63 (-0.87 to -0.39) | <0.001 |
| United States of America | 25070.9057 (18526.0439,33332.5437) | 12.5303 (9.2592,16.6594) | 23632.1729 (17348.2012,31576.8088) | 13.3396 (9.7925,17.824) | -0.05739 | 0.064587 | 0.37 (-0.12 to 0.86) | 0.138 |
| United States Virgin Islands | 17.2385 (12.8744,22.9619) | 15.4807 (11.5617,20.6206) | 5.0127 (3.6735,6.6101) | 14.3786 (10.537,18.9604) | -0.70921 | -0.07119 | -0.23 (-0.25 to -0.20) | <0.001 |

**Table S3 Numbers and ASRs per 100,000 Cases of deaths of urogenital congenital anomalies in 1990 and 2021, along with the relative changes and AAPC in ASRs per 100,000 Cases from 1990-2021, Categorized by 204 countries and territories**

| Characteristic | Number in 1990  （95% CI） | Age-standardized  Rate in 1990  （95% CI） | Number in 2019  （95% CI） | Age-standardized  Rate in 2019  （95% CI） | Relative Change of numbers from 1990 to 2019（%） | Relative Change of age-standardized  rate from 1990 to 2019（%） | AAPC  (Age-standardized  Rate, 95% CI) | *P* value |
| --- | --- | --- | --- | --- | --- | --- | --- | --- |
| American Samoa | 0.0177 (0.003,0.0701) | 0.0234 (0.0045,0.088) | 0.0147 (0.0016,0.0743) | 0.0424 (0.0042,0.2198) | -0.16949 | 0.811966 | 2.05 (1.46 to 2.64) | <0.001 |
| Antigua and Barbuda | 0.0534 (0.0287,0.0912) | 0.0908 (0.0491,0.1551) | 0.0455 (0.0236,0.0757) | 0.0807 (0.0407,0.137) | -0.14794 | -0.11123 | -0.46 (-1.08 to 0.15) | 0.141 |
| Arab Republic of Egypt | 559.8796 (170.346,1316.5476) | 0.6343 (0.1945,1.4891) | 232.43 (103.794,420.2266) | 0.1866 (0.0835,0.3377) | -0.58486 | -0.70582 | -3.93 (-4.41 to -3.44) | <0.001 |
| Argentine Republic | 77.6907 (51.76,135.3619) | 0.2328 (0.1551,0.4057) | 65.8624 (35.2705,95.6161) | 0.2524 (0.1344,0.3678) | -0.15225 | 0.084192 | 0.17 (-0.63 to 0.97) | 0.674 |
| Australia | 20.8172 (11.3133,49.8533) | 0.1631 (0.0882,0.3948) | 10.9188 (7.1269,26.1669) | 0.0688 (0.0436,0.1696) | -0.47549 | -0.57817 | -2.31 (-3.61 to -0.99) | <0.001 |
| Barbados | 0.5581 (0.3792,0.799) | 0.2695 (0.1844,0.3865) | 0.4299 (0.2192,0.6717) | 0.2916 (0.1438,0.465) | -0.22971 | 0.082004 | 0.29 (-1.65 to 2.27) | 0.773 |
| Belize | 0.28 (0.171,0.5307) | 0.0952 (0.0586,0.1794) | 0.3654 (0.2231,0.5837) | 0.0967 (0.0588,0.1549) | 0.305 | 0.015756 | 0.01 (-1.25 to 1.29) | 0.986 |
| Bermuda | 0.0313 (0.0138,0.059) | 0.0673 (0.0291,0.1292) | 0.0076 (0.0018,0.0192) | 0.0237 (0.0042,0.0624) | -0.75719 | -0.64785 | -3.33 (-3.53 to -3.14) | <0.001 |
| Bolivarian Republic of Venezuela | 13.5911 (8.8525,34.5159) | 0.054 (0.0354,0.1346) | 32.334 (18.8795,51.9758) | 0.1424 (0.0834,0.2321) | 1.379057 | 1.637037 | 3.44 (2.44 to 4.45) | <0.001 |
| Bosnia and Herzegovina | 1.5893 (0.4055,4.5032) | 0.0484 (0.0123,0.1372) | 0.3712 (0.1313,0.8237) | 0.0264 (0.0091,0.0584) | -0.76644 | -0.45455 | -1.89 (-2.70 to -1.07) | <0.001 |
| Brunei Darussalam | 0.4446 (0.1695,0.9248) | 0.1321 (0.0506,0.2742) | 0.507 (0.2397,0.9602) | 0.1699 (0.0798,0.3241) | 0.140351 | 0.286147 | 0.77 (0.33 to 1.21) | <0.001 |
| Burkina Faso | 24.9054 (7.9387,71.0092) | 0.1238 (0.0426,0.3529) | 49.1888 (17.1294,124.1103) | 0.1172 (0.0422,0.2885) | 0.975025 | -0.05331 | -0.17 (-0.30 to -0.04) | 0.014 |
| Canada | 29.9141 (19.0036,48.1602) | 0.1509 (0.0954,0.2444) | 15.1909 (10.2012,20.8023) | 0.0802 (0.0526,0.1087) | -0.49218 | -0.46852 | -1.80 (-2.04 to -1.56) | <0.001 |
| Central African Republic | 7.149 (2.1387,23.2435) | 0.127 (0.0398,0.4065) | 10.0421 (2.8806,29.5254) | 0.1155 (0.0339,0.334) | 0.404686 | -0.09055 | -0.33 (-0.45 to -0.22) | <0.001 |
| Commonwealth of Dominica | 0.0062 (0.0013,0.0354) | 0.0071 (0.0016,0.0408) | 0.0051 (0.0014,0.0279) | 0.0144 (0.0035,0.0807) | -0.17742 | 1.028169 | 2.24 (1.82 to 2.67) | <0.001 |
| Commonwealth of the Bahamas | 0.3507 (0.2045,0.5227) | 0.1335 (0.0781,0.1979) | 0.2303 (0.1244,0.3753) | 0.1015 (0.0509,0.1774) | -0.34331 | -0.2397 | -0.88 (-1.66 to -0.09) | 0.03 |
| Cook Islands | 0.0025 (5e-04,0.0091) | 0.0123 (0.0025,0.0435) | 0.0016 (3e-04,0.0059) | 0.0127 (0.0017,0.0493) | -0.36 | 0.03252 | 0.48 (-0.31 to 1.28) | 0.233 |
| Czech Republic | 11.3708 (5.2249,24.5274) | 0.178 (0.0801,0.3882) | 2.9089 (1.4787,4.6365) | 0.0524 (0.0261,0.0857) | -0.74418 | -0.70562 | -4.02 (-4.56 to -3.47) | <0.001 |
| Democratic People's Republic of Korea | 2.8502 (0.703,10.8864) | 0.0122 (0.0032,0.0441) | 2.5969 (1.0665,5.8021) | 0.011 (0.0038,0.032) | -0.08887 | -0.09836 | -0.30 (-0.48 to -0.12) | <0.001 |
| Democratic Republic of Sao Tome and Principe | 0.1581 (0.0509,0.4326) | 0.0774 (0.0257,0.2091) | 0.1012 (0.0232,0.2424) | 0.0452 (0.0106,0.1056) | -0.3599 | -0.41602 | -1.80 (-2.69 to -0.90) | <0.001 |
| Democratic Republic of the Congo | 82.9109 (26.2169,230.7614) | 0.1025 (0.0338,0.2842) | 94.3699 (24.4266,265.4476) | 0.0713 (0.0187,0.1974) | 0.138209 | -0.30439 | -1.13 (-1.23 to -1.04) | <0.001 |
| Democratic Republic of Timor-Leste | 0.7183 (0.1882,3.1478) | 0.0477 (0.0143,0.2039) | 0.5786 (0.1732,1.7058) | 0.0313 (0.0098,0.0901) | -0.19449 | -0.34382 | -1.34 (-1.63 to -1.06) | <0.001 |
| Democratic Socialist Republic of Sri Lanka | 23.4483 (16.1902,36.1889) | 0.1345 (0.093,0.2076) | 11.7288 (7.0332,20.723) | 0.0711 (0.042,0.1297) | -0.4998 | -0.47138 | -1.62 (-2.03 to -1.20) | <0.001 |
| Dominican Republic | 7.0479 (2.9176,14.9211) | 0.0674 (0.0284,0.1432) | 6.1676 (1.8939,14.4241) | 0.0596 (0.018,0.1401) | -0.1249 | -0.11573 | -0.48 (-1.42 to 0.46) | 0.316 |
| Eastern Republic of Uruguay | 5.5328 (4.0992,8.957) | 0.2053 (0.1521,0.3326) | 2.8207 (1.4797,4.558) | 0.161 (0.0839,0.2628) | -0.49019 | -0.21578 | -0.79 (-2.14 to 0.58) | 0.258 |
| Federal Democratic Republic of Ethiopia | 112.1386 (38.1314,381.2641) | 0.1061 (0.0373,0.3559) | 104.3103 (34.6093,263.5873) | 0.0646 (0.0213,0.1607) | -0.06981 | -0.39114 | -1.59 (-1.73 to -1.45) | <0.001 |
| Federal Democratic Republic of Nepal | 43.1434 (11.9185,101.9883) | 0.1193 (0.0336,0.2832) | 21.3708 (6.5363,55.1039) | 0.0695 (0.0213,0.1793) | -0.50466 | -0.41744 | -1.75 (-1.87 to -1.62) | <0.001 |
| Federal Republic of Germany | 87.6442 (59.8985,140.2803) | 0.1962 (0.1332,0.3135) | 42.5219 (26.259,56.4905) | 0.1012 (0.0617,0.1363) | -0.51483 | -0.4842 | -2.11 (-2.77 to -1.45) | <0.001 |
| Federal Republic of Nigeria | 186.1018 (72.481,550.7384) | 0.1054 (0.0465,0.3072) | 473.7943 (204.0335,1198.3441) | 0.127 (0.0602,0.3126) | 1.545888 | 0.204934 | 0.60 (0.50 to 0.71) | <0.001 |
| Federal Republic of Somalia | 13.5098 (3.5267,44.323) | 0.0797 (0.0214,0.2592) | 28.3843 (7.541,84.5793) | 0.0664 (0.0182,0.195) | 1.101016 | -0.16688 | -0.60 (-0.70 to -0.51) | <0.001 |
| Federated States of Micronesia | 0.0464 (0.01,0.1712) | 0.0329 (0.0084,0.117) | 0.0419 (0.0109,0.1288) | 0.0453 (0.0114,0.1411) | -0.09698 | 0.3769 | 1.04 (0.90 to 1.18) | <0.001 |
| Federative Republic of Brazil | 307.5863 (213.1279,540.4577) | 0.1986 (0.1376,0.3488) | 375.6883 (193.9116,502.2937) | 0.2308 (0.1187,0.3094) | 0.221408 | 0.162135 | 0.46 (0.25 to 0.67) | <0.001 |
| French Republic | 41.6595 (27.6324,69.4261) | 0.1077 (0.0705,0.1826) | 25.5338 (15.0028,34.4362) | 0.07 (0.0403,0.0955) | -0.38708 | -0.35005 | -1.29 (-1.66 to -0.92) | <0.001 |
| Gabonese Republic | 1.4861 (0.4899,4.6661) | 0.0912 (0.0316,0.2806) | 1.8039 (0.451,5.825) | 0.088 (0.022,0.284) | 0.213848 | -0.03509 | -0.11 (-0.30 to 0.08) | 0.247 |
| Georgia | 4.8801 (2.7621,8.0867) | 0.1112 (0.062,0.1857) | 2.3492 (1.4159,3.4082) | 0.0964 (0.0572,0.1459) | -0.51862 | -0.13309 | -0.39 (-2.66 to 1.94) | 0.742 |
| Grand Duchy of Luxembourg | 0.0905 (0.045,0.2625) | 0.0357 (0.0175,0.1037) | 0.0623 (0.0315,0.213) | 0.0174 (0.0086,0.0601) | -0.3116 | -0.51261 | -2.26 (-4.39 to -0.07) | 0.043 |
| Greenland | 0.1768 (0.0686,0.3884) | 0.3059 (0.119,0.6718) | 0.0391 (0.0125,0.1) | 0.1002 (0.0301,0.2628) | -0.77885 | -0.67244 | -3.35 (-3.66 to -3.04) | <0.001 |
| Grenada | 0.0324 (0.012,0.1757) | 0.0289 (0.0108,0.1548) | 0.0065 (0.0012,0.0555) | 0.009 (0.0016,0.0794) | -0.79938 | -0.68858 | -3.33 (-6.15 to -0.42) | 0.025 |
| Guam | 0.025 (0.004,0.0866) | 0.0146 (0.0026,0.0494) | 0.0489 (0.0144,0.1187) | 0.0373 (0.0106,0.0921) | 0.956 | 1.554795 | 3.03 (2.78 to 3.28) | <0.001 |
| Hashemite Kingdom of Jordan | 24.7303 (13.8216,39.6834) | 0.3956 (0.2224,0.6323) | 19.185 (10.3871,36.0057) | 0.1819 (0.0979,0.342) | -0.22423 | -0.54019 | -2.56 (-2.99 to -2.12) | <0.001 |
| Hellenic Republic | 14.0315 (7.4758,25.7176) | 0.262 (0.1355,0.4895) | 4.9377 (3.3912,7.0361) | 0.1092 (0.0721,0.1583) | -0.6481 | -0.58321 | -2.73 (-3.72 to -1.72) | <0.001 |
| Hungary | 13.2743 (6.6525,26.1523) | 0.2135 (0.1052,0.4263) | 3.5434 (1.9559,5.1612) | 0.0757 (0.0404,0.1126) | -0.73306 | -0.64543 | -3.30 (-3.98 to -2.61) | <0.001 |
| Independent State of Papua New Guinea | 3.4803 (0.6652,14.0621) | 0.0523 (0.0124,0.2049) | 17.487 (3.1422,48.9813) | 0.1097 (0.0231,0.3033) | 4.024567 | 1.097514 | 2.44 (2.28 to 2.61) | <0.001 |
| Independent State of Samoa | 0.0655 (0.0127,0.2436) | 0.027 (0.0057,0.0964) | 0.102 (0.0218,0.3284) | 0.0361 (0.0082,0.116) | 0.557252 | 0.337037 | 0.94 (0.67 to 1.22) | <0.001 |
| Ireland | 5.7862 (3.376,9.1551) | 0.2148 (0.1243,0.3413) | 2.7322 (1.7809,3.9731) | 0.0959 (0.0622,0.1393) | -0.52781 | -0.55354 | -2.71 (-3.61 to -1.80) | <0.001 |
| Islamic Republic of Afghanistan | 109.324 (28.2883,306.2987) | 0.5568 (0.1424,1.5681) | 221.9565 (90.2538,527.014) | 0.3941 (0.1612,0.9275) | 1.030263 | -0.29221 | -1.11 (-1.42 to -0.80) | <0.001 |
| Islamic Republic of Iran | 406.6292 (231.4696,622.7835) | 0.5366 (0.3088,0.8202) | 44.0492 (25.1092,72.7646) | 0.0807 (0.045,0.1334) | -0.89167 | -0.84961 | -5.92 (-6.50 to -5.33) | <0.001 |
| Islamic Republic of Mauritania | 3.0456 (0.7968,8.0255) | 0.081 (0.0242,0.2068) | 3.7636 (0.9064,9.9783) | 0.0616 (0.0145,0.1579) | 0.23575 | -0.23951 | -0.87 (-1.06 to -0.68) | <0.001 |
| Islamic Republic of Pakistan | 337.0707 (119.8434,680.7387) | 0.172 (0.062,0.345) | 442.2154 (153.1504,998.0162) | 0.1503 (0.0528,0.337) | 0.311937 | -0.12616 | -0.44 (-0.62 to -0.25) | <0.001 |
| Jamaica | 5.1769 (3.2688,7.7002) | 0.1888 (0.1201,0.28) | 2.9381 (1.9198,4.6472) | 0.172 (0.11,0.2739) | -0.43246 | -0.08898 | -0.28 (-0.59 to 0.03) | 0.078 |
| Japan | 114.6658 (69.5828,155.154) | 0.1868 (0.1122,0.2537) | 32.092 (18.3597,47.7872) | 0.0735 (0.0423,0.1085) | -0.72013 | -0.60653 | -2.96 (-3.53 to -2.37) | <0.001 |
| Kingdom of Bahrain | 3.0777 (1.6323,5.5931) | 0.4877 (0.2597,0.8848) | 1.9616 (1.1523,3.0491) | 0.2144 (0.1245,0.3374) | -0.36264 | -0.56039 | -2.53 (-3.25 to -1.81) | <0.001 |
| Kingdom of Belgium | 7.6448 (4.6252,13.7201) | 0.1221 (0.0726,0.2218) | 3.5839 (2.3688,5.6512) | 0.0597 (0.039,0.094) | -0.5312 | -0.51106 | -2.33 (-2.86 to -1.79) | <0.001 |
| Kingdom of Bhutan | 1.6797 (0.511,4.5076) | 0.1635 (0.0512,0.4299) | 0.6568 (0.2125,1.6999) | 0.1084 (0.0346,0.2821) | -0.60898 | -0.337 | -1.27 (-1.40 to -1.13) | <0.001 |
| Kingdom of Cambodia | 12.1733 (3.3683,46.2304) | 0.0657 (0.0207,0.2416) | 8.2805 (2.8124,23.8512) | 0.0486 (0.0167,0.1393) | -0.31978 | -0.26027 | -0.92 (-1.19 to -0.65) | <0.001 |
| Kingdom of Denmark | 7.3956 (4.8293,15.0713) | 0.2327 (0.1508,0.4731) | 4.4102 (2.8801,5.8876) | 0.1333 (0.0856,0.1794) | -0.40367 | -0.42716 | -1.91 (-3.00 to -0.81) | <0.001 |
| Kingdom of Eswatini | 0.4407 (0.1425,1.0981) | 0.033 (0.012,0.0773) | 0.5464 (0.2028,1.3144) | 0.0429 (0.0172,0.1003) | 0.239846 | 0.3 | 0.91 (0.76 to 1.05) | <0.001 |
| Kingdom of Lesotho | 0.9065 (0.2753,2.1509) | 0.038 (0.0122,0.087) | 1.1009 (0.4108,2.6132) | 0.0568 (0.0224,0.1324) | 0.214451 | 0.494737 | 1.35 (1.23 to 1.48) | <0.001 |
| Kingdom of Morocco | 62.5925 (17.6819,174.8718) | 0.1685 (0.0475,0.4689) | 27.2611 (8.7054,81.3922) | 0.0876 (0.0278,0.2626) | -0.56447 | -0.48012 | -2.09 (-2.25 to -1.92) | <0.001 |
| Kingdom of Norway | 0.7814 (0.4121,2.9457) | 0.0263 (0.0137,0.0998) | 0.8607 (0.1107,1.5398) | 0.0289 (0.0036,0.0527) | 0.101485 | 0.098859 | 0.25 (-0.57 to 1.07) | 0.545 |
| Kingdom of Saudi Arabia | 60.3106 (23.0167,133.3884) | 0.2507 (0.0963,0.5553) | 20.0664 (8.3356,48.1687) | 0.0748 (0.0279,0.2015) | -0.66728 | -0.70164 | -3.88 (-4.21 to -3.55) | <0.001 |
| Kingdom of Spain | 21.031 (12.1321,37.4845) | 0.103 (0.0586,0.1844) | 8.7881 (4.5951,14.429) | 0.0469 (0.0237,0.076) | -0.58214 | -0.54466 | -2.49 (-3.09 to -1.88) | <0.001 |
| Kingdom of Sweden | 6.3392 (2.3839,9.1096) | 0.1037 (0.0387,0.1497) | 0.3926 (0.1258,2.2799) | 0.0063 (0.0018,0.0385) | -0.93807 | -0.93925 | -8.29 (-14.29 to -1.87) | 0.012 |
| Kingdom of Thailand | 22.9899 (9.366,53.8162) | 0.0453 (0.0183,0.1052) | 6.0803 (3.184,12.377) | 0.0141 (0.0058,0.0302) | -0.73552 | -0.68874 | -3.68 (-4.01 to -3.35) | <0.001 |
| Kingdom of the Netherlands | 15.709 (10.7308,27.9617) | 0.1605 (0.109,0.286) | 7.9196 (5.2681,10.3827) | 0.085 (0.0559,0.1119) | -0.49586 | -0.4704 | -2.01 (-2.83 to -1.18) | <0.001 |
| Kingdom of Tonga | 0.022 (0.0035,0.0808) | 0.0151 (0.0027,0.0536) | 0.036 (0.0074,0.1283) | 0.0262 (0.0056,0.092) | 0.636364 | 0.735099 | 1.82 (1.54 to 2.11) | <0.001 |
| Kyrgyz Republic | 6.8977 (3.4545,9.475) | 0.1115 (0.057,0.153) | 4.9815 (3.3555,7.3722) | 0.0663 (0.0447,0.098) | -0.2778 | -0.40538 | -1.68 (-3.39 to 0.05) | 0.057 |
| Lao People's Democratic Republic | 5.9445 (1.088,22.6126) | 0.0795 (0.0179,0.2983) | 4.1437 (1.4094,14.143) | 0.0504 (0.0176,0.17) | -0.30294 | -0.36604 | -1.44 (-1.65 to -1.23) | <0.001 |
| Lebanese Republic | 12.0476 (4.5065,27.0261) | 0.2986 (0.1119,0.6675) | 4.6211 (2.2986,8.7094) | 0.1171 (0.057,0.2224) | -0.61643 | -0.60784 | -2.98 (-3.28 to -2.68) | <0.001 |
| Malaysia | 17.2028 (8.3493,35.4349) | 0.0759 (0.0385,0.152) | 11.9071 (6.1076,24.54) | 0.0467 (0.0219,0.1014) | -0.30784 | -0.38472 | -1.58 (-2.39 to -0.75) | <0.001 |
| Mongolia | 2.4978 (0.8795,5.83) | 0.0752 (0.0269,0.1735) | 1.7881 (0.8103,3.4764) | 0.0489 (0.0223,0.0949) | -0.28413 | -0.34973 | -1.26 (-2.14 to -0.37) | 0.006 |
| Montenegro | 0.8709 (0.3013,1.9299) | 0.1801 (0.062,0.3997) | 0.1309 (0.0313,0.3729) | 0.0377 (0.0088,0.1076) | -0.8497 | -0.79067 | -4.81 (-5.45 to -4.16) | <0.001 |
| New Zealand | 3.2952 (0.71,4.9191) | 0.1131 (0.0244,0.1688) | 1.6702 (0.2943,2.6628) | 0.0543 (0.0091,0.0865) | -0.49314 | -0.51989 | -2.45 (-3.62 to -1.26) | <0.001 |
| North Macedonia | 0.4036 (0.1252,1.189) | 0.0245 (0.0076,0.0723) | 0.0611 (0.0153,0.2633) | 0.0063 (0.0014,0.0265) | -0.84861 | -0.74286 | -4.39 (-5.39 to -3.38) | <0.001 |
| Northern Mariana Islands | 0.0068 (0.0012,0.0225) | 0.013 (0.0027,0.0419) | 0.007 (0.0021,0.0177) | 0.0202 (0.0054,0.0543) | 0.029412 | 0.553846 | 1.49 (1.08 to 1.91) | <0.001 |
| Palestine | 9.5969 (3.4955,20.0963) | 0.2348 (0.0857,0.4875) | 6.7702 (3.1303,12.86) | 0.1164 (0.0539,0.2212) | -0.29454 | -0.50426 | -2.29 (-2.44 to -2.14) | <0.001 |
| People's Democratic Republic of Algeria | 161.9511 (69.0044,340.4423) | 0.4414 (0.1882,0.9268) | 108.7407 (42.9004,233.3748) | 0.2468 (0.0967,0.5309) | -0.32856 | -0.44087 | -1.83 (-2.04 to -1.62) | <0.001 |
| People's Republic of Bangladesh | 392.4543 (113.3618,951.195) | 0.2014 (0.0597,0.4794) | 150.9956 (50.5417,367.2855) | 0.1104 (0.0367,0.2705) | -0.61525 | -0.45184 | -1.96 (-2.07 to -1.85) | <0.001 |
| People's Republic of China | 265.9907 (79.9532,655.9275) | 0.0238 (0.0071,0.0588) | 123.9085 (45.5877,200.0232) | 0.0136 (0.0042,0.0269) | -0.53416 | -0.42857 | -1.84 (-2.40 to -1.28) | <0.001 |
| Plurinational State of Bolivia | 31.457 (11.2681,61.2369) | 0.3017 (0.1098,0.5803) | 25.9967 (12.0574,50.9096) | 0.2223 (0.1033,0.4349) | -0.17358 | -0.26318 | -0.96 (-1.16 to -0.77) | <0.001 |
| Portuguese Republic | 5.2937 (2.512,10.3745) | 0.0906 (0.0415,0.1787) | 1.403 (0.8299,3.2749) | 0.0299 (0.0169,0.07) | -0.73497 | -0.66998 | -3.31 (-4.63 to -1.97) | <0.001 |
| Principality of Andorra | 0.0145 (0.0065,0.031) | 0.0521 (0.0219,0.1122) | 0.0029 (0.0011,0.0063) | 0.0075 (0.0022,0.0183) | -0.8 | -0.85605 | -5.86 (-7.01 to -4.70) | <0.001 |
| Principality of Monaco | 0.0097 (0.004,0.0188) | 0.0651 (0.0248,0.1338) | 0.0059 (0.0021,0.0135) | 0.03 (0.0087,0.0738) | -0.39175 | -0.53917 | -2.35 (-2.58 to -2.12) | <0.001 |
| Puerto Rico | 5.8107 (2.3723,8.2057) | 0.1814 (0.0735,0.2567) | 0.2188 (0.0651,0.7887) | 0.0199 (0.0063,0.0731) | -0.96235 | -0.8903 | -6.76 (-9.78 to -3.63) | <0.001 |
| Republic of Albania | 5.3168 (2.2226,10.7664) | 0.1365 (0.0572,0.2768) | 1.6358 (0.5984,3.9542) | 0.1212 (0.044,0.2949) | -0.69233 | -0.11209 | -0.37 (-0.56 to -0.18) | <0.001 |
| Republic of Angola | 23.8627 (6.7315,81.5924) | 0.1083 (0.0324,0.3658) | 47.9255 (16.7112,136.6338) | 0.0861 (0.0307,0.2429) | 1.008385 | -0.20499 | -0.73 (-0.90 to -0.56) | <0.001 |
| Republic of Armenia | 5.8156 (2.6378,9.721) | 0.1593 (0.0723,0.266) | 1.5255 (0.9541,2.2167) | 0.0781 (0.0524,0.1098) | -0.73769 | -0.50973 | -2.30 (-3.70 to -0.87) | 0.002 |
| Republic of Austria | 5.2898 (3.1324,8.9658) | 0.1131 (0.0659,0.1929) | 2.4416 (1.5357,3.81) | 0.0544 (0.0339,0.0848) | -0.53843 | -0.51901 | -2.07 (-2.85 to -1.29) | <0.001 |
| Republic of Azerbaijan | 6.1008 (2.5405,13.4263) | 0.0698 (0.0294,0.1531) | 4.191 (1.4058,9.603) | 0.0605 (0.019,0.1428) | -0.31304 | -0.13324 | -0.44 (-0.73 to -0.16) | 0.003 |
| Republic of Belarus | 8.8928 (6.1387,13.0536) | 0.1208 (0.0809,0.1747) | 1.9699 (1.1003,3.7922) | 0.0348 (0.0176,0.0653) | -0.77848 | -0.71192 | -4.13 (-4.82 to -3.44) | <0.001 |
| Republic of Benin | 10.8088 (3.2507,30.7452) | 0.1055 (0.0365,0.2949) | 23.0276 (7.7231,53.0853) | 0.0993 (0.0336,0.2211) | 1.130449 | -0.05877 | -0.22 (-0.54 to 0.10) | 0.175 |
| Republic of Botswana | 0.5968 (0.1482,1.5478) | 0.0305 (0.0084,0.0758) | 0.9351 (0.2962,2.2906) | 0.0404 (0.013,0.0986) | 0.566857 | 0.32459 | 0.84 (0.64 to 1.03) | <0.001 |
| Republic of Bulgaria | 3.966 (2.2964,6.8451) | 0.0799 (0.046,0.1391) | 1.1246 (0.6436,1.7566) | 0.0373 (0.0215,0.0586) | -0.71644 | -0.53317 | -2.47 (-3.46 to -1.47) | <0.001 |
| Republic of Burundi | 12.0275 (4.4267,44.9445) | 0.1047 (0.043,0.3858) | 14.8793 (4.643,40.1773) | 0.0695 (0.0218,0.1849) | 0.237107 | -0.3362 | -1.33 (-1.57 to -1.08) | <0.001 |
| Republic of Cabo Verde | 0.3828 (0.1169,0.9496) | 0.0673 (0.0205,0.163) | 0.235 (0.0564,0.5919) | 0.054 (0.0127,0.1375) | -0.3861 | -0.19762 | -0.69 (-1.06 to -0.32) | <0.001 |
| Republic of Cameroon | 21.8727 (7.0104,60.9791) | 0.1071 (0.04,0.2879) | 50.9279 (19.5475,133.9626) | 0.1114 (0.0443,0.2804) | 1.328377 | 0.040149 | 0.13 (-0.05 to 0.31) | 0.146 |
| Republic of Chad | 10.397 (3.2096,32.181) | 0.0775 (0.0262,0.236) | 34.3744 (12.6864,86.7613) | 0.0941 (0.0361,0.2267) | 2.306184 | 0.214194 | 0.64 (0.49 to 0.78) | <0.001 |
| Republic of Chile | 55.1212 (36.0402,83.7567) | 0.3762 (0.2462,0.5717) | 26.6945 (13.4721,35.7764) | 0.2683 (0.1338,0.3608) | -0.51571 | -0.28682 | -1.18 (-1.71 to -0.64) | <0.001 |
| Republic of Colombia | 73.0252 (52.7218,117.2356) | 0.1713 (0.1253,0.2738) | 72.0698 (45.0119,101.3493) | 0.1915 (0.1153,0.2799) | -0.01308 | 0.117922 | 0.30 (-0.15 to 0.76) | 0.192 |
| Republic of Costa Rica | 8.7481 (6.6508,13.3725) | 0.2288 (0.1742,0.3499) | 11.3421 (6.4712,14.558) | 0.328 (0.1884,0.4317) | 0.296522 | 0.433566 | 1.47 (1.20 to 1.74) | <0.001 |
| Republic of C么te d'Ivoire | 23.7773 (8.0516,63.1312) | 0.0992 (0.0387,0.2529) | 45.6801 (18.3874,108.9725) | 0.1082 (0.0455,0.2503) | 0.921164 | 0.090726 | 0.27 (0.02 to 0.51) | 0.031 |
| Republic of Croatia | 4.4464 (2.5381,8.1858) | 0.161 (0.0912,0.2994) | 1.6077 (0.9492,2.3968) | 0.0911 (0.0532,0.1367) | -0.63843 | -0.43416 | -1.66 (-2.57 to -0.74) | <0.001 |
| Republic of Cuba | 9.4874 (6.3017,19.2479) | 0.1042 (0.0683,0.2142) | 3.8456 (2.6704,6.7586) | 0.0542 (0.034,0.1146) | -0.59466 | -0.47985 | -2.00 (-3.26 to -0.72) | 0.002 |
| Republic of Cyprus | 0.9155 (0.3327,2.0809) | 0.1375 (0.0495,0.3135) | 0.2547 (0.0915,0.5585) | 0.0323 (0.0104,0.0723) | -0.72179 | -0.76509 | -4.57 (-4.80 to -4.33) | <0.001 |
| Republic of Djibouti | 0.4995 (0.1398,1.5069) | 0.0709 (0.0204,0.2099) | 0.9372 (0.1752,2.6917) | 0.0654 (0.0122,0.1873) | 0.876276 | -0.07757 | -0.17 (-0.39 to 0.05) | 0.13 |
| Republic of Ecuador | 11.1071 (6.9772,24.0426) | 0.0801 (0.051,0.1735) | 24.2778 (11.7542,34.1507) | 0.1542 (0.0741,0.2181) | 1.185791 | 0.925094 | 2.43 (1.49 to 3.39) | <0.001 |
| Republic of El Salvador | 25.6848 (15.8268,39.443) | 0.3278 (0.206,0.4994) | 15.4135 (8.053,24.3329) | 0.2572 (0.1342,0.4102) | -0.3999 | -0.21538 | -0.81 (-1.10 to -0.52) | <0.001 |
| Republic of Equatorial Guinea | 0.8181 (0.2383,2.4354) | 0.09 (0.0285,0.2614) | 1.6006 (0.4236,4.9476) | 0.0898 (0.0237,0.2771) | 0.956485 | -0.00222 | -0.00 (-0.33 to 0.33) | 0.997 |
| Republic of Estonia | 0.9526 (0.5604,1.2689) | 0.0819 (0.0499,0.111) | 0.1365 (0.0671,0.2438) | 0.015 (0.0066,0.0297) | -0.85671 | -0.81685 | -5.44 (-5.85 to -5.02) | <0.001 |
| Republic of Fiji | 0.2114 (0.0515,0.8168) | 0.0244 (0.0063,0.0922) | 0.4647 (0.0906,1.5167) | 0.0528 (0.0101,0.1733) | 1.198202 | 1.163934 | 2.54 (2.19 to 2.90) | <0.001 |
| Republic of Finland | 6.7855 (4.8339,11.5503) | 0.2056 (0.1459,0.346) | 2.3279 (1.4827,3.9762) | 0.0855 (0.0527,0.1536) | -0.65693 | -0.58414 | -2.95 (-3.49 to -2.41) | <0.001 |
| Republic of Ghana | 26.4447 (8.9351,69.7974) | 0.1 (0.0375,0.2585) | 39.4555 (12.1162,100.51) | 0.0905 (0.0281,0.2307) | 0.492 | -0.095 | -0.33 (-0.47 to -0.18) | <0.001 |
| Republic of Guatemala | 16.7964 (11.8416,29.4836) | 0.1137 (0.0817,0.1962) | 30.9066 (19.23,49.9047) | 0.2062 (0.1275,0.3365) | 0.840073 | 0.813544 | 2.10 (0.70 to 3.51) | 0.003 |
| Republic of Guinea | 17.8295 (5.7997,53.1586) | 0.1399 (0.0487,0.4184) | 24.475 (7.878,56.9914) | 0.1104 (0.0356,0.2499) | 0.372725 | -0.21086 | -0.71 (-1.04 to -0.37) | <0.001 |
| Republic of Guinea-Bissau | 2.3003 (0.7848,6.7369) | 0.12 (0.047,0.3336) | 2.7636 (0.9264,6.706) | 0.0901 (0.0309,0.2076) | 0.201409 | -0.24917 | -0.92 (-1.11 to -0.74) | <0.001 |
| Republic of Guyana | 2.1888 (1.4567,3.6435) | 0.1832 (0.1229,0.2997) | 1.6867 (0.9826,2.5724) | 0.2314 (0.1343,0.3539) | -0.2294 | 0.2631 | 0.81 (-0.23 to 1.86) | 0.127 |
| Republic of Haiti | 29.8846 (8.9636,72.4798) | 0.2715 (0.0798,0.6779) | 31.507 (11.7252,91.2123) | 0.1984 (0.0735,0.5877) | 0.054289 | -0.26924 | -1.00 (-1.13 to -0.87) | <0.001 |
| Republic of Honduras | 18.1087 (9.5667,32.2007) | 0.2458 (0.1398,0.414) | 17.9685 (8.7299,36.2309) | 0.1694 (0.0828,0.3416) | -0.00774 | -0.31082 | -1.21 (-1.46 to -0.95) | <0.001 |
| Republic of Iceland | 0.2958 (0.1835,0.5201) | 0.1312 (0.0811,0.2311) | 0.1447 (0.0874,0.2657) | 0.0609 (0.0362,0.1134) | -0.51082 | -0.53582 | -2.57 (-3.16 to -1.99) | <0.001 |
| Republic of India | 2297.7916 (991.9225,4055.4297) | 0.1962 (0.0858,0.3438) | 1824.0371 (766.5852,3743.8738) | 0.1702 (0.0707,0.3523) | -0.20618 | -0.13252 | -0.46 (-0.74 to -0.17) | 0.002 |
| Republic of Indonesia | 84.5286 (40.5739,219.9955) | 0.0383 (0.0187,0.0995) | 51.7868 (20.8522,109.8626) | 0.0234 (0.0092,0.0501) | -0.38735 | -0.38903 | -1.60 (-1.83 to -1.38) | <0.001 |
| Republic of Iraq | 149.9998 (69.478,315.9217) | 0.4628 (0.2176,0.9685) | 103.8575 (40.5376,190.9359) | 0.2572 (0.0997,0.4755) | -0.30762 | -0.44425 | -1.92 (-2.15 to -1.69) | <0.001 |
| Republic of Italy | 44.4519 (26.7933,66.8983) | 0.156 (0.0918,0.2377) | 17.4271 (8.342,23.9396) | 0.0773 (0.0332,0.1112) | -0.60796 | -0.50449 | -2.40 (-3.09 to -1.72) | <0.001 |
| Republic of Kazakhstan | 11.1619 (6.1444,17.2914) | 0.0626 (0.0346,0.097) | 7.3405 (4.9649,12.4636) | 0.0371 (0.0251,0.0632) | -0.34236 | -0.40735 | -1.67 (-2.28 to -1.06) | <0.001 |
| Republic of Kenya | 26.3528 (10.5752,80.0278) | 0.0588 (0.0233,0.1764) | 28.7089 (8.921,69.2174) | 0.051 (0.0158,0.1226) | 0.089406 | -0.13265 | -0.44 (-0.63 to -0.25) | <0.001 |
| Republic of Kiribati | 0.0608 (0.0103,0.2486) | 0.051 (0.0118,0.2022) | 0.1188 (0.0213,0.3537) | 0.0857 (0.0167,0.2546) | 0.953947 | 0.680392 | 1.69 (1.52 to 1.85) | <0.001 |
| Republic of Korea | 19.2571 (10.2417,41.626) | 0.0587 (0.0311,0.1251) | 1.9587 (1.0074,8.76) | 0.0138 (0.0072,0.0556) | -0.89829 | -0.76491 | -4.45 (-4.85 to -4.05) | <0.001 |
| Republic of Latvia | 2.6181 (1.6527,3.2845) | 0.1337 (0.086,0.168) | 0.3466 (0.2003,0.554) | 0.0283 (0.0157,0.0478) | -0.86761 | -0.78833 | -4.97 (-5.73 to -4.20) | <0.001 |
| Republic of Liberia | 7.8465 (2.2983,24.4722) | 0.1585 (0.0536,0.4859) | 6.732 (2.2898,15.836) | 0.0923 (0.0321,0.2106) | -0.14204 | -0.41767 | -1.87 (-2.69 to -1.04) | <0.001 |
| Republic of Lithuania | 5.0249 (3.0673,6.461) | 0.1705 (0.1061,0.2197) | 0.9006 (0.5237,1.3533) | 0.0523 (0.0275,0.0832) | -0.82077 | -0.69326 | -3.31 (-3.93 to -2.69) | <0.001 |
| Republic of Madagascar | 19.3541 (6.1887,65.1357) | 0.0832 (0.0273,0.2779) | 27.5776 (7.4716,68.5634) | 0.0695 (0.0187,0.1727) | 0.424897 | -0.16466 | -0.60 (-0.76 to -0.43) | <0.001 |
| Republic of Malawi | 26.9701 (7.8894,83.0181) | 0.1222 (0.0388,0.3672) | 20.6857 (5.6609,51.4399) | 0.0777 (0.0213,0.1919) | -0.23301 | -0.36416 | -1.44 (-1.81 to -1.08) | <0.001 |
| Republic of Maldives | 0.4921 (0.1168,1.3502) | 0.127 (0.0319,0.3356) | 0.1369 (0.0498,0.3038) | 0.0444 (0.015,0.1013) | -0.7218 | -0.65039 | -3.56 (-3.87 to -3.25) | <0.001 |
| Republic of Mali | 29.5424 (10.397,76.1481) | 0.1631 (0.0558,0.4002) | 57.2198 (17.7859,124.3842) | 0.1284 (0.0382,0.301) | 0.93687 | -0.21275 | -0.76 (-1.00 to -0.53) | <0.001 |
| Republic of Malta | 0.8061 (0.4954,1.3988) | 0.2946 (0.1791,0.5141) | 0.4493 (0.2788,0.5948) | 0.203 (0.1224,0.2714) | -0.44262 | -0.31093 | -1.48 (-2.85 to -0.10) | 0.036 |
| Republic of Mauritius | 1.2022 (0.5687,1.9939) | 0.1088 (0.0516,0.1804) | 0.55 (0.3935,0.938) | 0.0757 (0.0527,0.134) | -0.54251 | -0.30423 | -1.13 (-2.14 to -0.11) | 0.03 |
| Republic of Moldova | 9.4657 (4.3408,13.1616) | 0.2405 (0.1082,0.337) | 2.0274 (1.3617,2.8625) | 0.1188 (0.0818,0.1726) | -0.78582 | -0.50603 | -2.27 (-3.29 to -1.24) | <0.001 |
| Republic of Mozambique | 31.5299 (8.3908,109.2625) | 0.1176 (0.0335,0.4072) | 41.2134 (11.4899,114.4213) | 0.0816 (0.0227,0.2222) | 0.307121 | -0.30612 | -1.18 (-1.46 to -0.89) | <0.001 |
| Republic of Namibia | 0.7318 (0.189,1.8507) | 0.0336 (0.0102,0.0818) | 0.9424 (0.2842,2.4421) | 0.0362 (0.0121,0.0913) | 0.287784 | 0.077381 | 0.25 (0.13 to 0.36) | <0.001 |
| Republic of Nauru | 0.0046 (0.001,0.0165) | 0.031 (0.0082,0.1052) | 0.0085 (0.0021,0.0242) | 0.0632 (0.0172,0.1785) | 0.847826 | 1.03871 | 2.33 (2.15 to 2.51) | <0.001 |
| Republic of Nicaragua | 8.8198 (5.5239,16.1496) | 0.137 (0.0885,0.2468) | 7.1953 (4.2352,11.3744) | 0.113 (0.0659,0.1821) | -0.18419 | -0.17518 | -0.63 (-0.84 to -0.41) | <0.001 |
| Republic of Niue | 7e-04 (2e-04,0.0024) | 0.0302 (0.0068,0.1046) | 0.002 (5e-04,0.0067) | 0.1759 (0.0421,0.586) | 1.857143 | 4.824503 | 6.21 (5.21 to 7.22) | <0.001 |
| Republic of Palau | 0.0053 (0.0011,0.018) | 0.0356 (0.0076,0.12) | 0.0056 (0.0016,0.0169) | 0.0557 (0.0124,0.1676) | 0.056604 | 0.564607 | 1.47 (1.23 to 1.71) | <0.001 |
| Republic of Panama | 7.3137 (5.5487,11.4292) | 0.2609 (0.1984,0.4058) | 11.0391 (6.6317,15.1602) | 0.3011 (0.1799,0.4148) | 0.509373 | 0.154082 | 0.46 (0.14 to 0.78) | 0.005 |
| Republic of Paraguay | 4.224 (2.4231,9.939) | 0.0686 (0.0395,0.1617) | 4.3775 (2.0026,8.266) | 0.0699 (0.032,0.1322) | 0.03634 | 0.01895 | 0.37 (0.22 to 0.52) | <0.001 |
| Republic of Peru | 38.0912 (16.4309,78.0963) | 0.1271 (0.0556,0.2594) | 32.3224 (13.6462,73.9954) | 0.0984 (0.0408,0.2267) | -0.15145 | -0.22581 | -0.90 (-1.36 to -0.44) | <0.001 |
| Republic of Poland | 47.3393 (14.2951,72.4661) | 0.1782 (0.053,0.2733) | 14.3452 (3.024,22.3919) | 0.0852 (0.0172,0.1338) | -0.69697 | -0.52189 | -2.45 (-3.22 to -1.67) | <0.001 |
| Republic of Rwanda | 15.7324 (5.6339,54.8727) | 0.1125 (0.0435,0.381) | 11.6202 (3.521,28.6871) | 0.0673 (0.0203,0.1647) | -0.26138 | -0.40178 | -1.55 (-2.22 to -0.87) | <0.001 |
| Republic of San Marino | 0.0068 (0.003,0.0141) | 0.0532 (0.0222,0.1131) | 0.0017 (6e-04,0.0045) | 0.011 (0.0028,0.031) | -0.75 | -0.79323 | -4.84 (-5.03 to -4.65) | <0.001 |
| Republic of Senegal | 13.9522 (4.0994,37.5515) | 0.094 (0.0294,0.243) | 17.0514 (4.3792,42.1217) | 0.0788 (0.0205,0.1919) | 0.22213 | -0.1617 | -0.56 (-0.82 to -0.30) | <0.001 |
| Republic of Serbia | 5.9218 (2.7275,11.4879) | 0.0887 (0.0406,0.1728) | 0.7854 (0.436,1.9142) | 0.0228 (0.0125,0.0545) | -0.86737 | -0.74295 | -4.13 (-5.27 to -2.98) | <0.001 |
| Republic of Seychelles | 0.0304 (0.0105,0.0703) | 0.0401 (0.0147,0.0905) | 0.0368 (0.0122,0.0856) | 0.0433 (0.013,0.1045) | 0.210526 | 0.0798 | 0.27 (0.01 to 0.53) | 0.043 |
| Republic of Sierra Leone | 12.5616 (3.5035,37.9803) | 0.1429 (0.0481,0.4253) | 13.3967 (4.8523,30.7295) | 0.0999 (0.036,0.2298) | 0.06648 | -0.30091 | -1.11 (-1.48 to -0.74) | <0.001 |
| Republic of Singapore | 2.2488 (1.2374,5.137) | 0.0925 (0.0509,0.209) | 0.5971 (0.2717,2.1339) | 0.0217 (0.0101,0.0736) | -0.73448 | -0.76541 | -4.67 (-5.65 to -3.68) | <0.001 |
| Republic of Slovenia | 1.285 (0.6807,2.9092) | 0.1154 (0.06,0.264) | 0.3685 (0.2125,0.5515) | 0.0385 (0.0219,0.059) | -0.71323 | -0.66638 | -3.20 (-4.26 to -2.13) | <0.001 |
| Republic of South Africa | 27.9861 (19.5033,40.858) | 0.0585 (0.041,0.0841) | 31.1107 (17.7165,44.5448) | 0.0638 (0.0362,0.0918) | 0.111648 | 0.090598 | 0.23 (-0.24 to 0.70) | 0.345 |
| Republic of South Sudan | 11.1988 (3.2187,34.4186) | 0.0984 (0.03,0.3015) | 21.5753 (6.7213,59.4043) | 0.1245 (0.0401,0.3373) | 0.926572 | 0.265244 | 0.76 (0.61 to 0.91) | <0.001 |
| Republic of Sudan | 198.2922 (33.879,631.8969) | 0.5079 (0.0851,1.6198) | 237.7311 (118.5294,483.6983) | 0.4313 (0.2153,0.8762) | 0.198893 | -0.15082 | -0.53 (-0.59 to -0.46) | <0.001 |
| Republic of Suriname | 0.4406 (0.1814,0.91) | 0.1012 (0.042,0.2082) | 0.4556 (0.1986,0.9597) | 0.1009 (0.042,0.2185) | 0.034044 | -0.00296 | 0.03 (-0.44 to 0.50) | 0.914 |
| Republic of Tajikistan | 6.8869 (2.3159,17.8934) | 0.0726 (0.0254,0.1854) | 13.2026 (3.9767,35.7722) | 0.0986 (0.0302,0.2649) | 0.91706 | 0.358127 | 1.00 (0.65 to 1.35) | <0.001 |
| Republic of the Congo | 3.6242 (1.1045,10.3312) | 0.088 (0.028,0.2491) | 4.613 (1.2397,13.7953) | 0.0763 (0.0209,0.2278) | 0.272833 | -0.13295 | -0.42 (-0.63 to -0.22) | <0.001 |
| Republic of the Gambia | 1.3833 (0.3604,4.0231) | 0.0726 (0.0199,0.2044) | 2.3802 (0.6237,6.0038) | 0.0711 (0.0205,0.1741) | 0.720668 | -0.02066 | -0.09 (-0.48 to 0.30) | 0.661 |
| Republic of the Marshall Islands | 0.0218 (0.0039,0.0862) | 0.034 (0.0087,0.1278) | 0.0378 (0.0059,0.1413) | 0.0685 (0.0107,0.2563) | 0.733945 | 1.014706 | 2.29 (2.15 to 2.44) | <0.001 |
| Republic of the Niger | 16.3141 (3.3821,53.0122) | 0.0891 (0.0203,0.2777) | 32.444 (7.9083,84.6369) | 0.0639 (0.0155,0.1621) | 0.988709 | -0.28283 | -1.16 (-1.52 to -0.80) | <0.001 |
| Republic of the Philippines | 41.4949 (28.6026,67.6163) | 0.0458 (0.0323,0.0725) | 36.0971 (22.7601,56.707) | 0.0329 (0.0207,0.0515) | -0.13008 | -0.28166 | -1.11 (-1.36 to -0.85) | <0.001 |
| Republic of the Union of Myanmar | 40.1174 (11.7947,137.7784) | 0.0786 (0.0249,0.2681) | 28.956 (10.6128,84.5063) | 0.0555 (0.02,0.1625) | -0.27822 | -0.29389 | -1.13 (-1.29 to -0.97) | <0.001 |
| Republic of Trinidad and Tobago | 4.0284 (2.4151,5.6658) | 0.3453 (0.2071,0.4856) | 2.0469 (1.2446,3.1958) | 0.2519 (0.1504,0.4004) | -0.49188 | -0.27049 | -0.98 (-1.96 to 0.00) | 0.051 |
| Republic of Tunisia | 45.3192 (18.2748,94.2838) | 0.4314 (0.1741,0.8969) | 13.539 (5.0902,33.0522) | 0.1625 (0.0608,0.3966) | -0.70125 | -0.62332 | -3.14 (-3.39 to -2.88) | <0.001 |
| Republic of Turkey | 574.2511 (242.1014,1175.715) | 0.8164 (0.3444,1.669) | 109.2174 (51.5913,199.9183) | 0.2143 (0.0993,0.3982) | -0.80981 | -0.73751 | -4.28 (-4.52 to -4.03) | <0.001 |
| Republic of Uganda | 32.6978 (9.6731,108.3959) | 0.0797 (0.0252,0.2617) | 62.3903 (22.2834,169.6747) | 0.0856 (0.0307,0.23) | 0.908089 | 0.074028 | 0.25 (-0.13 to 0.63) | 0.2 |
| Republic of Uzbekistan | 7.4498 (3.859,19.795) | 0.0227 (0.0119,0.0594) | 8.7231 (5.3437,19.1181) | 0.023 (0.0141,0.0502) | 0.170917 | 0.013216 | 0.06 (-0.64 to 0.76) | 0.87 |
| Republic of Vanuatu | 0.071 (0.0137,0.269) | 0.027 (0.0062,0.0978) | 0.1883 (0.0486,0.5459) | 0.0467 (0.0136,0.1331) | 1.652113 | 0.72963 | 1.82 (1.59 to 2.04) | <0.001 |
| Republic of Yemen | 122.3668 (24.3441,340.8649) | 0.4069 (0.0807,1.1261) | 150.8352 (64.7674,304.2552) | 0.3255 (0.1402,0.6554) | 0.232648 | -0.20005 | -0.70 (-0.90 to -0.49) | <0.001 |
| Republic of Zambia | 16.5993 (5.6146,56.7695) | 0.1 (0.0367,0.338) | 23.1597 (6.2909,55.5163) | 0.0824 (0.0226,0.1965) | 0.395221 | -0.176 | -0.61 (-0.74 to -0.49) | <0.001 |
| Republic of Zimbabwe | 4.4917 (0.9226,13.7244) | 0.0273 (0.0062,0.0813) | 9.119 (2.4487,24.8165) | 0.0455 (0.0135,0.1185) | 1.030189 | 0.666667 | 1.66 (1.41 to 1.91) | <0.001 |
| Romania | 20.5688 (14.0769,42.5859) | 0.1354 (0.0924,0.2831) | 5.5828 (3.6539,7.7874) | 0.0614 (0.0404,0.0867) | -0.72858 | -0.54653 | -2.14 (-3.29 to -0.97) | <0.001 |
| Russian Federation | 130.3193 (85.6393,161.9333) | 0.1227 (0.0808,0.1528) | 42.5823 (25.4043,69.0473) | 0.0464 (0.026,0.0768) | -0.67325 | -0.62184 | -3.28 (-3.88 to -2.67) | <0.001 |
| Saint Kitts and Nevis | 0.0612 (0.0365,0.0986) | 0.1382 (0.0828,0.2223) | 0.0358 (0.0216,0.0594) | 0.1099 (0.0655,0.1905) | -0.41503 | -0.20478 | -0.77 (-1.04 to -0.49) | <0.001 |
| Saint Lucia | 0.2555 (0.1666,0.3776) | 0.1528 (0.0999,0.2248) | 0.152 (0.0978,0.2505) | 0.1657 (0.1031,0.2824) | -0.40509 | 0.084424 | 0.22 (-0.19 to 0.63) | 0.29 |
| Saint Vincent and the Grenadines | 0.3151 (0.1753,0.4443) | 0.2577 (0.1431,0.3623) | 0.1174 (0.071,0.1779) | 0.1635 (0.0979,0.2544) | -0.62742 | -0.36554 | -1.58 (-2.38 to -0.77) | <0.001 |
| Slovak Republic | 13.7312 (6.6169,22.6401) | 0.3528 (0.1684,0.5839) | 4.7022 (2.5153,7.1162) | 0.1672 (0.089,0.2539) | -0.65755 | -0.52608 | -2.36 (-3.14 to -1.58) | <0.001 |
| Socialist Republic of Viet Nam | 14.3965 (3.6489,39.4377) | 0.0162 (0.0042,0.0431) | 8.2475 (2.5629,20.7028) | 0.0102 (0.0029,0.0264) | -0.42712 | -0.37037 | -1.47 (-1.62 to -1.31) | <0.001 |
| Solomon Islands | 0.1853 (0.035,0.7412) | 0.0313 (0.0064,0.1196) | 0.4773 (0.1084,1.5458) | 0.0515 (0.0124,0.1642) | 1.575823 | 0.645367 | 1.63 (1.51 to 1.76) | <0.001 |
| State of Eritrea | 4.8497 (1.5109,14.7771) | 0.0747 (0.0246,0.2206) | 6.2681 (1.6384,17.9573) | 0.0697 (0.0183,0.1974) | 0.292472 | -0.06693 | -0.22 (-0.50 to 0.06) | 0.126 |
| State of Israel | 13.6942 (9.0165,23.5637) | 0.2707 (0.1783,0.4658) | 7.7703 (4.7676,11.4601) | 0.0868 (0.0532,0.1282) | -0.43258 | -0.67935 | -3.65 (-4.58 to -2.72) | <0.001 |
| State of Kuwait | 6.7503 (3.8786,15.6945) | 0.4049 (0.2325,0.9457) | 11.4966 (6.5933,16.0539) | 0.4589 (0.2612,0.6454) | 0.703124 | 0.133366 | 1.25 (0.35 to 2.15) | 0.006 |
| State of Libya | 29.1555 (12.4386,62.1632) | 0.4684 (0.2007,0.9971) | 17.4527 (8.9918,34.948) | 0.4276 (0.215,0.8816) | -0.40139 | -0.08711 | -0.52 (-1.27 to 0.23) | 0.17 |
| State of Qatar | 0.9954 (0.417,2.0862) | 0.1852 (0.0782,0.3858) | 1.4526 (0.6738,2.7969) | 0.0755 (0.0344,0.1463) | 0.459313 | -0.59233 | -2.69 (-2.88 to -2.49) | <0.001 |
| Sultanate of Oman | 11.2775 (3.8346,28.5653) | 0.3324 (0.1141,0.8362) | 8.1522 (3.4279,17.398) | 0.21 (0.087,0.4508) | -0.27713 | -0.36823 | -1.52 (-1.86 to -1.17) | <0.001 |
| Swiss Confederation | 9.3882 (6.3736,18.9304) | 0.2269 (0.154,0.4576) | 5.5125 (3.5028,7.4794) | 0.1238 (0.078,0.1691) | -0.41283 | -0.45439 | -2.10 (-3.15 to -1.05) | <0.001 |
| Syrian Arab Republic | 85.3402 (30.7039,189.2326) | 0.393 (0.1425,0.8677) | 14.3594 (4.3893,37.5541) | 0.1427 (0.0406,0.3796) | -0.83174 | -0.6369 | -3.20 (-3.72 to -2.69) | <0.001 |
| Taiwan (Province of China) | 10.9172 (7.8982,14.0897) | 0.0649 (0.0469,0.0839) | 6.1123 (4.3377,8.2706) | 0.0546 (0.0379,0.0788) | -0.44012 | -0.15871 | -0.47 (-1.65 to 0.72) | 0.434 |
| Togolese Republic | 6.2829 (2.0162,17.8501) | 0.0902 (0.0296,0.2445) | 8.4383 (2.5631,19.1164) | 0.0776 (0.0236,0.17) | 0.343058 | -0.13969 | -0.46 (-0.57 to -0.35) | <0.001 |
| Tokelau | 4e-04 (1e-04,0.0014) | 0.0238 (0.0052,0.081) | 0.0013 (3e-04,0.0042) | 0.1444 (0.0286,0.4651) | 2.25 | 5.067227 | 7.28 (6.48 to 8.09) | <0.001 |
| Turkmenistan | 5.8785 (3.1708,9.3091) | 0.1018 (0.0558,0.1586) | 7.8293 (5.0783,11.8099) | 0.1474 (0.0958,0.2218) | 0.331853 | 0.447937 | 1.13 (-0.37 to 2.66) | 0.141 |
| Tuvalu | 0.0073 (0.0012,0.03) | 0.0476 (0.01,0.1889) | 0.0062 (0.0016,0.0177) | 0.0482 (0.0128,0.1386) | -0.15068 | 0.012605 | 0.07 (-0.04 to 0.18) | 0.193 |
| Ukraine | 45.545 (31.1882,72.0178) | 0.1288 (0.0847,0.2018) | 9.5645 (6.3139,20.8945) | 0.0464 (0.0303,0.0896) | -0.79 | -0.63975 | -3.23 (-3.72 to -2.75) | <0.001 |
| Union of the Comoros | 0.9053 (0.2686,2.6849) | 0.101 (0.0313,0.2967) | 0.6236 (0.1419,1.5737) | 0.0769 (0.0175,0.1933) | -0.31117 | -0.23861 | -0.89 (-1.03 to -0.75) | <0.001 |
| United Arab Emirates | 10.654 (4.5669,22.0538) | 0.4664 (0.2021,0.959) | 6.6993 (2.9091,13.5948) | 0.1602 (0.0667,0.3335) | -0.37119 | -0.65652 | -3.21 (-4.00 to -2.41) | <0.001 |
| United Kingdom of Great Britain and Northern Ireland | 44.3952 (32.5291,71.3627) | 0.1111 (0.0807,0.1798) | 25.0551 (18.3705,38.7677) | 0.0688 (0.0497,0.1074) | -0.43563 | -0.38074 | -1.62 (-2.24 to -0.99) | <0.001 |
| United Mexican States | 342.818 (241.6844,608.8564) | 0.3006 (0.215,0.5288) | 382.9207 (261.2715,484.692) | 0.3598 (0.2494,0.4692) | 0.11698 | 0.196939 | 0.60 (0.33 to 0.87) | <0.001 |
| United Republic of Tanzania | 57.7742 (18.9576,199.676) | 0.1078 (0.0368,0.3695) | 79.9354 (26.9751,206.969) | 0.0907 (0.0306,0.2329) | 0.383583 | -0.15863 | -0.52 (-0.67 to -0.37) | <0.001 |
| United States of America | 420.9948 (309.9471,583.2051) | 0.2081 (0.1528,0.2891) | 243.1644 (168.9603,323.4452) | 0.131 (0.0897,0.1752) | -0.42241 | -0.37049 | -1.43 (-1.88 to -0.99) | <0.001 |
| United States Virgin Islands | 0.0688 (0.0216,0.1688) | 0.0621 (0.0196,0.1523) | 0.013 (0.0045,0.0313) | 0.0272 (0.0067,0.0774) | -0.81105 | -0.562 | -2.55 (-2.79 to -2.32) | <0.001 |

**Table S4 Numbers and ASRs per 100,000 Cases of DALYs of urogenital congenital anomalies in 1990 and 2021, along with the relative changes and AAPC in ASRs per 100,000 Cases from 1990-2021, Categorized by 204 countries and territories**

| Characteristic | Number in 1990  （95% CI） | Age-standardized  Rate in 1990  （95% CI） | Number in 2019  （95% CI） | Age-standardized  Rate in 2019  （95% CI） | Relative Change of numbers from 1990 to 2019（%） | Relative Change of age-standardized  rate from 1990 to 2019（%） | AAPC  (Age-standardized  Rate, 95% CI) | *P* value |
| --- | --- | --- | --- | --- | --- | --- | --- | --- |
| American Samoa | 3.7498  (1.8341,8.642) | 5.3363 (2.7138,11.5842) | 3.2887 (1.6378,8.56) | 8.1313 (3.7373,23.7457) | -0.12297 | 0.523771 | 1.41 (1.20 to 1.63) | <0.001 |
| Antigua and Barbuda | 5.6066  (3.2739,9.4337) | 9.3617 (5.4287,15.7939) | 5.1365 (3.1156,8.0557) | 8.9788 (5.2916,14.4376) | -0.08385 | -0.0409 | -0.15 (-0.53 to 0.24) | 0.447 |
| Arab Republic of Egypt | 52844.1002 (17890.2842,119230.8486) | 60.3056 (20.7167,134.9482) | 25250.1473 (13554.7078,42568.5151) | 20.281 (10.9473,34.0486) | -0.52218 | -0.6637 | -3.40 (-4.34 to -2.46) | <0.001 |
| Argentine Republic | 8134.8133 (5605.6847,13421.1164) | 24.3048 (16.7413,40.1468) | 7321.9712 (4510.6963,10140.776) | 26.4777 (15.7724,36.998) | -0.09992 | 0.089402 | 0.21 (-0.49 to 0.91) | 0.565 |
| Australia | 2152.7996 (1310.1468,4688.2181) | 16.8026 (10.0874,37.3013) | 1381.4545 (928.6262,2685.6667) | 8.6325 (5.7035,17.7327) | -0.3583 | -0.48624 | -1.85 (-2.92 to -0.77) | <0.001 |
| Barbados | 52.8733  (37.5662,73.4817) | 25.5356 (18.1003,35.6147) | 39.7659 (22.4762,60.0061) | 27.8678 (15.2767,43.2998) | -0.2479 | 0.091331 | 0.30 (-1.54 to 2.17) | 0.752 |
| Belize | 29.8543 (20.0112,53.0103) | 10.2167 (6.8873,18.0418) | 40.2916 (27.2672,60.7764) | 10.3946 (6.9582,15.8549) | 0.349608 | 0.017413 | 0.01 (-1.08 to 1.10) | 0.99 |
| Bermuda | 3.4712 (1.8715,5.9435) | 7.5781 (4.0057,13.1251) | 1.2584 (0.6188,2.1544) | 4.0476 (1.8479,7.2557) | -0.63747 | -0.46588 | -2.04 (-2.26 to -1.81) | <0.001 |
| Bolivarian Republic of Venezuela | 1591.9913 (1081.5461,3437.9231) | 6.3628 (4.3415,13.4324) | 3091.3712 (1955.0039,4941.0053) | 13.7163 (8.5806,22.2874) | 0.941827 | 1.155702 | 2.66 (1.77 to 3.56) | <0.001 |
| Bosnia and Herzegovina | 320.8162 (182.5687,608.3895) | 8.7554 (4.8398,17.376) | 140.142 (89.463,218.0206) | 7.8487 (4.9776,12.1429) | -0.56317 | -0.10356 | -0.33 (-0.68 to 0.02) | 0.065 |
| Brunei Darussalam | 55.6327 (30.3308,101.1993) | 16.7707 (9.1979,30.2587) | 68.9617 (41.5693,114.6013) | 21.4445 (12.7162,35.995) | 0.239589 | 0.278688 | 0.76 (0.46 to 1.07) | <0.001 |
| Burkina Faso | 2765.9318 (1203.3582,7046.3111) | 14.3634 (6.9814,35.1755) | 5653.3013 (2632.1599,12591.8583) | 13.904 (6.91,29.4414) | 1.043905 | -0.03198 | -0.11 (-0.25 to 0.03) | 0.135 |
| Canada | 3032.0788 (1996.0699,4659.8116) | 15.1899 (9.9561,23.5579) | 1774.6625 (1289.2322,2343.9305) | 9.0488 (6.5336,11.9509) | -0.4147 | -0.40429 | -1.50 (-1.70 to -1.30) | <0.001 |
| Central African Republic | 832.0918 (348.8846,2292.6587) | 15.9126 (7.3293,40.864) | 1259.6238 (561.1498,3016.7385) | 15.0677 (7.0006,34.657) | 0.513804 | -0.0531 | -0.19 (-0.27 to -0.11) | <0.001 |
| Commonwealth of Dominica | 1.9783 (1.0898,4.8397) | 2.3193 (1.2841,5.5598) | 1.5396 (0.8926,3.4376) | 3.4038 (1.8972,8.7777) | -0.22176 | 0.467598 | 1.27 (1.09 to 1.45) | <0.001 |
| Commonwealth of the Bahamas | 35.4688 (22.0743,51.2187) | 13.274 (8.2626,19.2208) | 24.775 (15.3215,39.6668) | 10.6714 (6.1759,18.0084) | -0.3015 | -0.19607 | -0.70 (-1.39 to -0.00) | 0.05 |
| Cook Islands | 0.8235 (0.4355,1.4962) | 3.7889 (2.0135,6.9244) | 0.5655 (0.3145,0.9909) | 4.281 (2.3243,8.2271) | -0.3133 | 0.129879 | 0.46 (0.32 to 0.60) | <0.001 |
| Czech Republic | 1290.4196 (686.0968,2467.4827) | 19.4906 (10.025,38.174) | 497.1013 (335.513,726.4004) | 8.4446 (5.5501,12.3517) | -0.61478 | -0.56673 | -2.75 (-3.15 to -2.35) | <0.001 |
| Democratic People's Republic of Korea | 684.7683 (386.2593,1428.0989) | 3.0209 (1.7169,6.0253) | 542.0053 (322.7055,879.6074) | 2.7255 (1.5676,4.8591) | -0.20848 | -0.09779 | -0.33 (-0.42 to -0.23) | <0.001 |
| Democratic Republic of Sao Tome and Principe | 20.062 (10.0445,45.7678) | 10.1117 (5.3258,22.3159) | 17.3621 (8.942,30.9696) | 7.1096 (3.6096,12.7981) | -0.13458 | -0.29689 | -1.10 (-1.65 to -0.54) | <0.001 |
| Democratic Republic of the Congo | 10302.7617 (5089.2815,24353.9803) | 13.9778 (7.4591,31.8667) | 14084.4647 (6950.8165,29629.4013) | 10.9424 (5.5184,22.3205) | 0.367057 | -0.21716 | -0.75 (-0.86 to -0.65) | <0.001 |
| Democratic Republic of Timor-Leste | 83.7872 (34.5742,296.9995) | 5.8411 (2.5923,19.4793) | 82.9284 (41.1862,186.3818) | 4.5245 (2.2904,9.8603) | -0.01025 | -0.2254 | -0.86 (-1.07 to -0.66) | <0.001 |
| Democratic Socialist Republic of Sri Lanka | 2348.9244 (1675.6229,3442.1023) | 13.2609 (9.4597,19.5533) | 1357.2123 (894.1722,2170.1626) | 8.2265 (5.2851,13.5833) | -0.4222 | -0.37964 | -1.21 (-1.58 to -0.84) | <0.001 |
| Dominican Republic | 757.6525 (389.598,1473.9002) | 7.3585 (3.8656,14.1227) | 713.4753 (315.5059,1478.3349) | 6.8469 (2.9749,14.2849) | -0.05831 | -0.06953 | -0.23 (-0.94 to 0.48) | 0.52 |
| Eastern Republic of Uruguay | 572.9574 (426.9323,873.9746) | 21.1126 (15.733,32.3233) | 324.4305 (208.4365,476.7363) | 17.3428 (10.619,26.1919) | -0.43376 | -0.17856 | -0.64 (-1.79 to 0.52) | 0.28 |
| Federal Democratic Republic of Ethiopia | 13414.9391 (6741.604,37702.6416) | 13.5844 (7.2759,36.0988) | 14712.8459 (7673.5679,28630.6221) | 9.3664 (4.9714,17.7166) | 0.096751 | -0.3105 | -1.18 (-1.26 to -1.11) | <0.001 |
| Federal Democratic Republic of Nepal | 5144.6446 (2178.0583,10590.68) | 15.2243 (7.0748,29.8177) | 3399.5716 (1822.6584,6653.9993) | 10.7354 (5.6745,21.2873) | -0.3392 | -0.29485 | -1.17 (-1.31 to -1.04) | <0.001 |
| Federal Republic of Germany | 9323.8011 (6800.5152,13961.2285) | 20.5811 (14.8565,31.0941) | 5189.2156 (3574.2074,6798.4594) | 12.2423 (8.2753,15.9718) | -0.44344 | -0.40517 | -1.67 (-2.18 to -1.16) | <0.001 |
| Federal Republic of Nigeria | 20958.7535 (10343.5716,54162.2574) | 12.4116 (6.4228,30.6717) | 52545.2202 (27092.8623,116123.9686) | 14.1814 (7.6717,30.2424) | 1.507078 | 0.142592 | 0.44 (0.33 to 0.55) | <0.001 |
| Federal Republic of Somalia | 1722.9874 (787.7398,4408.4152) | 11.1609 (5.45,26.1894) | 3828.9773 (1841.9767,9239.425) | 9.7351 (4.9624,21.8265) | 1.22229 | -0.12775 | -0.43 (-0.53 to -0.32) | <0.001 |
| Federated States of Micronesia | 7.6441 (3.5672,18.9471) | 5.2902 (2.5239,12.871) | 6.8377 (3.5344,14.4709) | 6.9762 (3.4841,15.1591) | -0.10549 | 0.318703 | 0.89 (0.77 to 1.01) | <0.001 |
| Federative Republic of Brazil | 30142.9211 (21110.2685,50894.1513) | 19.2521 (13.4602,32.6522) | 35729.7645 (19466.7484,47260.2565) | 21.944 (11.8504,29.1249) | 0.185345 | 0.139824 | 0.40 (0.21 to 0.58) | <0.001 |
| French Republic | 4764.229 (3380.8789,7241.5707) | 12.0493 (8.4379,18.7691) | 3419.3566 (2358.3142,4495.4613) | 9.1318 (6.2611,11.9838) | -0.28229 | -0.24213 | -0.83 (-1.12 to -0.53) | <0.001 |
| Gabonese Republic | 198.166 (97.1206,485.9504) | 12.9073 (6.5768,30.1401) | 270.9076 (130.6765,621.5912) | 12.9893 (6.215,29.974) | 0.367074 | 0.006353 | 0.01 (-0.15 to 0.17) | 0.866 |
| Georgia | 549.1148 (330.5895,819.6295) | 12.3691 (7.3503,18.7624) | 274.1041 (189.4611,381.3211) | 11.4338 (7.7721,16.3379) | -0.50083 | -0.07562 | -0.21 (-2.06 to 1.69) | 0.83 |
| Grand Duchy of Luxembourg | 13.2346 (7.9754,28.2814) | 5.145 (3.0647,11.1826) | 14.6915 (9.3169,26.8695) | 3.7523 (2.3994,7.3983) | 0.110083 | -0.27069 | -1.16 (-2.17 to -0.13) | 0.027 |
| Greenland | 16.4842 (6.6472,35.6348) | 28.5326 (11.5053,61.6098) | 4.1073 (1.7654,9.544) | 10.4226 (4.2404,24.9549) | -0.75083 | -0.63471 | -3.03 (-3.32 to -2.74) | <0.001 |
| Grenada | 4.6367 (2.4835,17.0565) | 4.1476 (2.2428,14.9793) | 2.2827 (1.1877,6.1953) | 2.8185 (1.4312,8.5728) | -0.50769 | -0.32045 | -1.01 (-2.62 to 0.64) | 0.228 |
| Guam | 5.826 (2.8385,11.0145) | 3.5858 (1.797,6.5565) | 8.1222 (4.3092,14.7989) | 6.1864 (3.2316,11.4071) | 0.39413 | 0.72525 | 1.77 (1.62 to 1.92) | <0.001 |
| Hashemite Kingdom of Jordan | 2451.4693 (1455.6495,3835.9179) | 39.7446 (23.7795,61.5972) | 2315.9597 (1491.7213,3820.5072) | 20.9417 (13.1012,35.5989) | -0.05528 | -0.47309 | -2.12 (-2.51 to -1.73) | <0.001 |
| Hellenic Republic | 1390.4112 (784.7936,2444.7245) | 25.6692 (14.0752,46.1378) | 542.4748 (401.9509,727.7833) | 11.979 (8.7103,16.0729) | -0.60985 | -0.53333 | -2.33 (-3.18 to -1.47) | <0.001 |
| Hungary | 1479.1896 (840.9772,2640.9338) | 23.0843 (12.6353,42.2863) | 541.4271 (377.2385,751.1464) | 10.9802 (7.51,15.699) | -0.63397 | -0.52434 | -2.40 (-2.74 to -2.06) | <0.001 |
| Independent State of Papua New Guinea | 462.8999 (184.4599,1451.2912) | 7.243 (3.2102,21.4316) | 2009.9399 (680.0874,4793.9682) | 13.0318 (4.8315,30.1503) | 3.342062 | 0.799227 | 1.91 (1.79 to 2.03) | <0.001 |
| Independent State of Samoa | 12.2242 (5.7898,28.2044) | 5.1052 (2.5285,11.3759) | 17.0389 (8.1505,37.2327) | 6.1831 (3.079,13.1647) | 0.393866 | 0.211138 | 0.66 (0.48 to 0.83) | <0.001 |
| Ireland | 547.9881 (332.5991,842.7271) | 20.1993 (12.1225,31.3658) | 293.0883 (198.6703,397.7586) | 10.0787 (6.7565,13.8111) | -0.46516 | -0.50104 | -2.36 (-3.18 to -1.54) | <0.001 |
| Islamic Republic of Afghanistan | 10347.1786 (3068.3835,27807.1416) | 53.5706 (16.5873,143.7608) | 21696.0509 (9730.5719,48081.0523) | 39.127 (17.9434,85.4459) | 1.096808 | -0.26962 | -1.01 (-1.28 to -0.73) | <0.001 |
| Islamic Republic of Iran | 39901.4667 (24543.6986,58707.1006) | 52.3903 (32.1148,77.1584) | 6437.9014 (4153.507,9355.0154) | 10.6733 (6.8111,15.9094) | -0.83866 | -0.79627 | -5.01 (-5.40 to -4.62) | <0.001 |
| Islamic Republic of Mauritania | 360.2324 (153.0359,814.5418) | 9.9296 (4.5813,21.0035) | 524.2439 (250.872,1073.5276) | 8.5275 (4.1387,17.028) | 0.455294 | -0.1412 | -0.50 (-0.58 to -0.43) | <0.001 |
| Islamic Republic of Pakistan | 36253.9158 (15881.1877,66773.14) | 19.0931 (8.7715,34.2832) | 49961.6894 (22819.5302,99822.2339) | 17.004 (7.8637,33.7621) | 0.378105 | -0.10942 | -0.36 (-0.52 to -0.20) | <0.001 |
| Jamaica | 515.4837 (336.3508,739.9655) | 18.6403 (12.1587,26.7677) | 302.9425 (211.6422,453.6866) | 17.3508 (11.8077,26.7879) | -0.41231 | -0.06918 | -0.24 (-0.48 to 0.01) | 0.06 |
| Japan | 13707.3805 (9689.1921,17955.4412) | 20.8302 (14.1647,27.1979) | 5124.983 (3471.0055,6966.0965) | 10.2648 (7.0412,13.753) | -0.62612 | -0.50722 | -2.24 (-2.62 to -1.85) | <0.001 |
| Kingdom of Bahrain | 299.0354 (172.0296,522.92) | 47.4514 (27.5751,82.7315) | 231.1598 (158.0162,335.8856) | 23.5705 (15.5263,34.8337) | -0.22698 | -0.50327 | -2.16 (-2.74 to -1.59) | <0.001 |
| Kingdom of Belgium | 844.2928 (555.1234,1367.1643) | 13.3189 (8.7054,21.9516) | 505.5516 (363.9329,740.5573) | 8.0508 (5.7702,11.7509) | -0.40121 | -0.39554 | -1.70 (-2.15 to -1.25) | <0.001 |
| Kingdom of Bhutan | 188.6617 (80.9107,445.291) | 18.8754 (8.4881,43.1058) | 90.5534 (46.7218,189.7701) | 14.1698 (7.1114,30.6437) | -0.52002 | -0.2493 | -0.89 (-0.99 to -0.80) | <0.001 |
| Kingdom of Cambodia | 1336.5169 (535.7666,4366.4374) | 7.3107 (3.1638,23.0608) | 1055.4276 (540.5196,2466.7873) | 6.0683 (3.0816,14.2793) | -0.21031 | -0.16994 | -0.56 (-0.75 to -0.37) | <0.001 |
| Kingdom of Denmark | 732.2465 (502.8998,1385.3417) | 23.1224 (15.7706,44.1705) | 453.6327 (323.6651,592.1459) | 13.8857 (9.7554,18.0158) | -0.38049 | -0.39947 | -1.75 (-2.68 to -0.81) | <0.001 |
| Kingdom of Eswatini | 105.8901 (61.0437,183.1158) | 8.4228 (4.9264,13.9135) | 133.7676 (77.2032,219.4661) | 9.7971 (5.7571,15.992) | 0.263268 | 0.163164 | 0.51 (0.37 to 0.65) | <0.001 |
| Kingdom of Lesotho | 194.6187 (113.247,322.7598) | 8.775 (5.1947,14.3985) | 224.9449 (132.6922,376.79) | 10.8337 (6.3942,18.275) | 0.155824 | 0.23461 | 0.72 (0.57 to 0.87) | <0.001 |
| Kingdom of Morocco | 6634.3067 (2468.0667,16832.37) | 18.2552 (6.9496,45.578) | 3720.0853 (1881.3755,8601.9828) | 11.4638 (5.7055,27.0734) | -0.43927 | -0.37203 | -1.49 (-1.62 to -1.36) | <0.001 |
| Kingdom of Norway | 158.3667 (99.8691,333.1819) | 5.1877 (3.2657,11.2344) | 176.8585 (89.4079,269.4396) | 5.475 (2.5973,8.7335) | 0.116766 | 0.055381 | 0.04 (-0.37 to 0.44) | 0.859 |
| Kingdom of Saudi Arabia | 6251.4532 (2833.9218,12640.7075) | 26.266 (12.103,52.628) | 3063.9394 (1795.8278,5620.5496) | 10.6484 (5.6999,21.2094) | -0.50988 | -0.59459 | -2.85 (-3.05 to -2.65) | <0.001 |
| Kingdom of Spain | 2459.4145 (1570.8446,3946.0599) | 11.349 (7.1951,18.8831) | 1331.8387 (867.9525,1882.2432) | 6.5669 (4.1926,9.3054) | -0.45847 | -0.42137 | -1.82 (-2.37 to -1.27) | <0.001 |
| Kingdom of Sweden | 713.6464 (346.6857,974.5108) | 11.8346 (5.7268,16.2182) | 171.3986 (98.8235,342.9376) | 2.6469 (1.5039,5.424) | -0.75983 | -0.77634 | -4.70 (-6.37 to -3.01) | <0.001 |
| Kingdom of Thailand | 3061.747 (1702.973,6117.7792) | 5.7346 (3.0972,11.7543) | 1166.998 (736.1645,1802.2349) | 3.0469 (1.7814,4.8434) | -0.61885 | -0.46868 | -2.00 (-2.19 to -1.81) | <0.001 |
| Kingdom of the Netherlands | 1599.8484 (1138.2918,2636.781) | 16.2151 (11.4153,27.0203) | 888.2726 (630.387,1141.7593) | 9.5653 (6.7077,12.2369) | -0.44478 | -0.4101 | -1.65 (-2.34 to -0.96) | <0.001 |
| Kingdom of Tonga | 5.4081 (2.6426,10.6085) | 3.8698 (1.9693,7.296) | 6.8551 (3.4245,14.896) | 5.1306 (2.6245,10.7938) | 0.267562 | 0.325805 | 0.94 (0.80 to 1.07) | <0.001 |
| Kyrgyz Republic | 765.3116 (450.7836,1042.3042) | 12.4942 (7.4079,17.048) | 643.8025 (485.9552,901.4768) | 8.4968 (6.3929,11.8846) | -0.15877 | -0.31994 | -1.26 (-2.46 to -0.04) | 0.043 |
| Lao People's Democratic Republic | 639.9373 (200.5414,2122.0563) | 8.6946 (3.0917,28.0363) | 525.9017 (261.2729,1415.8919) | 6.3561 (3.1952,16.9781) | -0.1782 | -0.26896 | -0.99 (-1.12 to -0.86) | <0.001 |
| Lebanese Republic | 1208.0378 (515.0079,2531.1686) | 30.3016 (13.2115,62.7085) | 603.782 (384.1311,990.6167) | 14.4378 (8.8038,24.0781) | -0.5002 | -0.52353 | -2.36 (-2.58 to -2.13) | <0.001 |
| Malaysia | 1878.2036 (1019.2081,3460.3315) | 8.141 (4.5462,14.7622) | 1498.8412 (870.9623,2594.1765) | 5.7506 (3.1901,10.4515) | -0.20198 | -0.29362 | -1.12 (-1.71 to -0.53) | <0.001 |
| Mongolia | 297.5892 (147.1897,594.0166) | 9.1212 (4.6614,17.8651) | 249.3371 (154.3533,404.9868) | 6.7955 (4.1933,11.0128) | -0.16214 | -0.25498 | -0.86 (-1.40 to -0.32) | 0.002 |
| Montenegro | 101.3134 (48.2961,199.6859) | 20.2653 (9.3708,40.7814) | 26.5277 (14.3645,50.7319) | 6.8647 (3.6388,13.92) | -0.73816 | -0.66126 | -3.31 (-3.73 to -2.89) | <0.001 |
| New Zealand | 386.674 (142.3019,552.0481) | 13.1956 (4.8005,18.8149) | 256.1742 (112.396,374.0807) | 7.8192 (3.1985,11.4019) | -0.33749 | -0.40744 | -1.74 (-2.54 to -0.94) | <0.001 |
| North Macedonia | 97.0375 (58.1046,166.0355) | 5.4661 (3.2061,9.5842) | 52.6344 (32.0436,83.0475) | 3.9655 (2.3545,6.6588) | -0.45759 | -0.27453 | -1.01 (-1.64 to -0.38) | 0.002 |
| Northern Mariana Islands | 2.132 (1.1328,3.7658) | 4.2135 (2.2891,7.1764) | 1.9912 (1.1445,3.0713) | 5.4207 (3.0224,8.5179) | -0.06604 | 0.286508 | 0.82 (0.63 to 1.00) | <0.001 |
| Palestine | 989.813 (444.079,1936.0143) | 25.0728 (12.051,47.8744) | 868.6515 (505.308,1434.4509) | 14.6383 (8.4494,24.2623) | -0.12241 | -0.41617 | -1.77 (-1.89 to -1.66) | <0.001 |
| People's Democratic Republic of Algeria | 15922.1364 (7439.4569,31610.3502) | 43.5896 (20.5576,86.1961) | 11621.3221 (5616.8726,22592.8241) | 26.2349 (12.5889,51.2478) | -0.27012 | -0.39814 | -1.60 (-1.78 to -1.41) | <0.001 |
| People's Republic of Bangladesh | 43463.2188 (18100.2294,94503.6811) | 23.05 (10.1576,48.6412) | 21383.3127 (11259.5765,41646.1102) | 14.7919 (7.5344,29.668) | -0.50801 | -0.35827 | -1.43 (-1.57 to -1.29) | <0.001 |
| People's Republic of China | 44134.2359 (24330.9189,82165.4158) | 3.8287 (2.0883,7.1526) | 23352.5272 (14862.4382,35780.8245) | 2.7125 (1.5952,4.3127) | -0.47088 | -0.29153 | -1.15 (-1.40 to -0.90) | <0.001 |
| Plurinational State of Bolivia | 2940.3027 (1146.132,5631.6665) | 27.9981 (11.1279,53.0226) | 2501.7022 (1274.1119,4867.6269) | 21.2434 (10.7913,41.3869) | -0.14917 | -0.24126 | -0.86 (-1.05 to -0.68) | <0.001 |
| Portuguese Republic | 591.5884 (325.5106,1046.4329) | 9.8023 (5.213,17.7101) | 217.3412 (144.5226,353.4486) | 4.4613 (2.93,7.4965) | -0.63261 | -0.54487 | -2.47 (-3.32 to -1.61) | <0.001 |
| Principality of Andorra | 2.1425 (1.2999,3.5654) | 6.9473 (3.9288,12.192) | 1.1743 (0.7151,1.8395) | 2.8682 (1.7283,4.6398) | -0.4519 | -0.58715 | -2.75 (-3.60 to -1.90) | <0.001 |
| Principality of Monaco | 1.1173 (0.6129,1.9943) | 7.7801 (4.1247,14.5481) | 0.8795 (0.4933,1.5808) | 4.6786 (2.45,8.8057) | -0.21283 | -0.39865 | -1.56 (-1.69 to -1.43) | <0.001 |
| Puerto Rico | 574.3617 (267.3389,792.2) | 17.8402 (8.2048,24.6865) | 60.7388 (34.7128,115.8013) | 4.1817 (2.3293,9.0633) | -0.89425 | -0.7656 | -4.64 (-6.44 to -2.82) | <0.001 |
| Republic of Albania | 614.0296 (327.3031,1115.8037) | 15.7646 (8.4034,28.6738) | 211.4028 (115.7166,429.1851) | 14.576 (7.6041,30.5635) | -0.65571 | -0.0754 | -0.26 (-0.45 to -0.08) | 0.005 |
| Republic of Angola | 2884.6295 (1277.379,8032.0357) | 14.3333 (6.7837,37.1232) | 6594.2955 (3211.062,14479.3631) | 12.4247 (6.4024,26.2164) | 1.286011 | -0.13316 | -0.42 (-0.54 to -0.30) | <0.001 |
| Republic of Armenia | 614.6281 (316.2266,954.4085) | 16.8037 (8.6438,26.0942) | 191.7476 (141.8958,256.6386) | 9.8434 (7.2837,13.0852) | -0.68803 | -0.41421 | -1.74 (-2.83 to -0.64) | 0.002 |
| Republic of Austria | 616.4491 (393.3049,935.1256) | 12.811 (8.0618,19.8822) | 373.0395 (263.5956,542.1207) | 7.8304 (5.537,11.4262) | -0.39486 | -0.38878 | -1.43 (-1.96 to -0.91) | <0.001 |
| Republic of Azerbaijan | 786.4148 (433.8343,1508.3705) | 9.0185 (5.0026,17.2076) | 621.8516 (342.0074,1095.2194) | 8.374 (4.3676,15.3619) | -0.20926 | -0.07146 | -0.22 (-0.38 to -0.07) | 0.006 |
| Republic of Belarus | 1074.8641 (776.2359,1462.5392) | 14.079 (9.9668,19.3336) | 343.2979 (226.5204,516.8904) | 6.0836 (3.8065,9.3195) | -0.68061 | -0.5679 | -2.88 (-3.28 to -2.47) | <0.001 |
| Republic of Benin | 1237.2563 (533.9631,2976.2933) | 12.6557 (6.0549,29.2991) | 2741.9469 (1282.1527,5336.7535) | 12.1093 (6.0226,22.5636) | 1.216151 | -0.04317 | -0.17 (-0.41 to 0.08) | 0.183 |
| Republic of Botswana | 151.4325 (86.8999,268.6507) | 7.9631 (4.7204,13.6487) | 241.792 (141.4385,394.1053) | 9.8802 (5.7305,16.261) | 0.596698 | 0.240748 | 0.68 (0.60 to 0.76) | <0.001 |
| Republic of Bulgaria | 579.064 (388.8038,856.8502) | 10.7435 (7.1984,16.3884) | 230.0378 (155.7819,324.06) | 6.7719 (4.5162,9.4312) | -0.60274 | -0.36967 | -1.49 (-2.06 to -0.93) | <0.001 |
| Republic of Burundi | 1489.0168 (779.5794,4335.1993) | 14.0276 (7.649,38.6609) | 2079.9993 (1028.9664,4352.0142) | 10.1517 (5.2735,20.507) | 0.396894 | -0.27631 | -1.06 (-1.23 to -0.88) | <0.001 |
| Republic of Cabo Verde | 51.2007 (24.6645,102.2055) | 9.2671 (4.6221,17.9328) | 38.4019 (20.5538,70.9269) | 8.1128 (4.155,15.3507) | -0.24997 | -0.12456 | -0.39 (-0.58 to -0.21) | <0.001 |
| Republic of Cameroon | 2483.763 (1112.7468,5920.1208) | 12.5456 (6.1449,28.5821) | 6003.3492 (3018.7652,13630.2838) | 12.9049 (6.8372,28.4826) | 1.417038 | 0.02864 | 0.10 (-0.01 to 0.21) | 0.07 |
| Republic of Chad | 1249.1679 (568.5191,3201.9003) | 10.0389 (5.0446,23.6953) | 4020.1493 (1977.2282,8843.8513) | 11.4093 (5.8308,23.429) | 2.218262 | 0.136509 | 0.41 (0.32 to 0.51) | <0.001 |
| Republic of Chile | 5296.4653 (3593.8569,7807.5594) | 36.1706 (24.5595,53.2766) | 2801.5543 (1666.8529,3680.5395) | 27.0556 (15.1874,35.9326) | -0.47105 | -0.252 | -1.02 (-1.53 to -0.52) | <0.001 |
| Republic of Colombia | 7086.9091 (5213.4466,10947.4119) | 16.5662 (12.215,25.5533) | 6623.3525 (4309.2736,9145.0964) | 17.9954 (11.5368,25.5513) | -0.06541 | 0.086272 | 0.21 (-0.18 to 0.61) | 0.281 |
| Republic of Costa Rica | 835.375 (650.2994,1236.3887) | 21.6482 (16.8884,32.0096) | 929.4577 (561.8547,1185.0774) | 28.3274 (16.6783,37.1747) | 0.112623 | 0.308534 | 1.17 (0.91 to 1.44) | <0.001 |
| Republic of C么te d'Ivoire | 2737.6173 (1223.1851,6255.8159) | 11.8183 (5.711,25.5581) | 5370.3891 (2760.3689,11177.0282) | 12.7749 (6.7393,25.6664) | 0.961702 | 0.080942 | 0.23 (0.05 to 0.41) | 0.012 |
| Republic of Croatia | 470.786 (302.5801,803.4139) | 16.4882 (10.4663,28.7347) | 206.5321 (142.5702,284.4456) | 10.9223 (7.4959,15.2516) | -0.5613 | -0.33757 | -1.18 (-1.93 to -0.43) | 0.002 |
| Republic of Cuba | 988.468 (684.7984,1812.4542) | 10.7845 (7.3733,20.176) | 411.1275 (297.8801,701.8846) | 6.3732 (4.4278,11.9673) | -0.58408 | -0.40904 | -1.60 (-2.59 to -0.61) | 0.002 |
| Republic of Cyprus | 100.0943 (46.8417,205.9929) | 14.8936 (6.8721,30.8855) | 46.0773 (27.8974,76.8141) | 5.4667 (3.2053,9.5523) | -0.53966 | -0.63295 | -3.20 (-3.37 to -3.03) | <0.001 |
| Republic of Djibouti | 69.9204 (33.5261,160.0579) | 10.6337 (5.3142,23.1478) | 146.5345 (66.6835,316.2803) | 10.1994 (4.6493,21.9322) | 1.095733 | -0.04084 | -0.11 (-0.30 to 0.07) | 0.226 |
| Republic of Ecuador | 1202.1868 (824.8518,2364.6933) | 8.6707 (5.9785,16.9125) | 2445.9091 (1344.2063,3392.9203) | 15.3474 (8.3036,21.3171) | 1.03455 | 0.77003 | 2.11 (1.30 to 2.93) | <0.001 |
| Republic of El Salvador | 2353.9128 (1485.0594,3606.2351) | 29.6606 (19.0473,44.9332) | 1363.246 (771.9496,2135.1845) | 22.8437 (12.8142,36.4516) | -0.42086 | -0.22983 | -0.87 (-1.13 to -0.60) | <0.001 |
| Republic of Equatorial Guinea | 102.6433 (48.36,247.0208) | 12.4967 (6.4161,28.5805) | 239.5662 (125.1258,537.0464) | 13.0178 (6.7948,29.5918) | 1.333968 | 0.041699 | 0.14 (-0.08 to 0.37) | 0.22 |
| Republic of Estonia | 124.6825 (86.6867,164.3696) | 10.5047 (7.2037,13.9101) | 38.8797 (25.0271,58.3888) | 4.6643 (2.9626,7.1014) | -0.68817 | -0.55598 | -2.67 (-2.89 to -2.46) | <0.001 |
| Republic of Fiji | 50.8046 (26.0003,108.0367) | 5.5505 (2.8713,11.8638) | 79.7152 (38.4798,173.8064) | 8.8134 (4.2128,19.5949) | 0.569055 | 0.587857 | 1.46 (1.33 to 1.60) | <0.001 |
| Republic of Finland | 672.44 (496.7764,1077.0203) | 20.4243 (14.9861,32.6661) | 271.2036 (186.9808,411.3365) | 9.9352 (6.636,16.0894) | -0.59669 | -0.51356 | -2.41 (-2.87 to -1.94) | <0.001 |
| Republic of Ghana | 3049.4622 (1423.8341,6939.5334) | 11.8259 (6.0303,25.6331) | 4953.8239 (2377.5389,10272.7747) | 11.1568 (5.4588,22.7456) | 0.624491 | -0.05658 | -0.19 (-0.30 to -0.07) | 0.001 |
| Republic of Guatemala | 1689.4031 (1223.634,2803.3745) | 11.3262 (8.3528,18.3101) | 2955.7071 (1890.9989,4602.5803) | 19.5465 (12.3883,30.8892) | 0.749557 | 0.725777 | 1.90 (0.70 to 3.12) | 0.002 |
| Republic of Guinea | 1915.0771 (825.8824,5155.5304) | 15.8202 (7.3911,40.7085) | 2852.6477 (1355.9298,5874.082) | 13.0455 (6.4589,25.8282) | 0.489573 | -0.17539 | -0.61 (-0.81 to -0.40) | <0.001 |
| Republic of Guinea-Bissau | 255.4059 (114.7986,654.8965) | 13.4888 (6.6873,32.6715) | 336.7304 (172.7455,706.6918) | 10.756 (5.695,21.5794) | 0.318413 | -0.2026 | -0.72 (-0.95 to -0.50) | <0.001 |
| Republic of Guyana | 214.737 (147.5087,346.2772) | 17.9902 (12.5271,28.5609) | 162.8365 (102.8561,243.5559) | 22.2749 (14.0358,33.4251) | -0.24169 | 0.238169 | 0.73 (-0.23 to 1.70) | 0.136 |
| Republic of Haiti | 2837.9237 (1011.0429,6548.9155) | 25.7631 (9.302,59.1285) | 3118.1597 (1385.3015,8041.6782) | 19.6224 (8.7503,50.8609) | 0.098747 | -0.23835 | -0.87 (-0.98 to -0.76) | <0.001 |
| Republic of Honduras | 1679.2241 (887.4637,2929.2827) | 21.9609 (12.4186,36.6373) | 1663.9014 (855.0357,3254.7733) | 15.3576 (7.798,30.3534) | -0.00912 | -0.30068 | -1.16 (-1.35 to -0.96) | <0.001 |
| Republic of Iceland | 31.8015 (20.9119,50.9697) | 14.1 (9.245,22.617) | 18.397 (12.5273,28.1394) | 7.7365 (5.2207,12.1296) | -0.42151 | -0.45131 | -1.97 (-2.16 to -1.78) | <0.001 |
| Republic of India | 250797.1031 (130234.0683,419349.6006) | 21.5701 (11.3694,35.715) | 218835.0657 (111962.6105,394563.6757) | 19.4734 (9.7417,35.9874) | -0.12744 | -0.0972 | -0.34 (-0.57 to -0.12) | 0.003 |
| Republic of Indonesia | 10914.1687 (6510.7956,23563.9052) | 4.865 (2.8995,10.5257) | 8221.2367 (4801.3807,13679.5463) | 3.5795 (2.0335,6.0965) | -0.24674 | -0.26423 | -1.00 (-1.18 to -0.83) | <0.001 |
| Republic of Iraq | 14532.7716 (7245.2524,29729.1274) | 45.4058 (23.0942,91.7597) | 11131.7101 (5550.5178,19051.5128) | 26.9992 (13.198,46.8086) | -0.23403 | -0.40538 | -1.70 (-1.91 to -1.50) | <0.001 |
| Republic of Italy | 4641.9764 (2898.9924,6753.4514) | 15.9895 (9.8899,23.5414) | 2014.0752 (1174.1854,2748.8684) | 8.7347 (4.671,12.0336) | -0.56612 | -0.45372 | -2.06 (-2.54 to -1.58) | <0.001 |
| Republic of Kazakhstan | 1486.0264 (964.3592,2083.7518) | 8.2715 (5.3692,11.6202) | 1210.0898 (867.4229,1810.5491) | 6.2883 (4.4869,9.3655) | -0.18569 | -0.23976 | -0.88 (-1.43 to -0.33) | 0.002 |
| Republic of Kenya | 3805.3957 (2153.8387,8707.8598) | 9.2088 (5.3199,19.8958) | 4828.8824 (2445.5543,8643.0449) | 8.2166 (4.1319,14.9007) | 0.268957 | -0.10774 | -0.36 (-0.45 to -0.28) | <0.001 |
| Republic of Kiribati | 8.2571 (3.3862,24.7503) | 7.2477 (3.2449,20.3406) | 15.2184 (5.6045,36.2362) | 10.8805 (4.0677,25.8888) | 0.843068 | 0.501235 | 1.30 (1.11 to 1.48) | <0.001 |
| Republic of Korea | 3177.9895 (2106.53,5032.2008) | 8.7101 (5.7594,14.463) | 1252.9379 (777.0572,2034.9943) | 5.1673 (3.2646,9.6069) | -0.60575 | -0.40675 | -1.64 (-1.83 to -1.44) | <0.001 |
| Republic of Latvia | 293.7027 (218.4494,372.2728) | 14.9563 (10.9878,18.8508) | 65.317 (43.0835,93.4247) | 5.8382 (3.8962,8.4566) | -0.77761 | -0.60965 | -3.16 (-3.57 to -2.75) | <0.001 |
| Republic of Liberia | 844.6735 (340.762,2380.5463) | 17.7142 (7.9084,48.0509) | 839.5511 (428.1003,1651.5355) | 11.3267 (5.9111,21.7286) | -0.00606 | -0.36059 | -1.52 (-2.02 to -1.02) | <0.001 |
| Republic of Lithuania | 526.4227 (363.2856,654.557) | 18.0183 (12.5729,22.4125) | 115.251 (77.9578,164.5751) | 7.3838 (4.7749,10.7718) | -0.78107 | -0.59021 | -2.61 (-3.03 to -2.19) | <0.001 |
| Republic of Madagascar | 2501.6818 (1216.879,6618.7304) | 11.686 (6.1616,29.2052) | 3934.8112 (1973.5251,7577.2245) | 10.0636 (5.1252,19.1263) | 0.572866 | -0.13883 | -0.48 (-0.57 to -0.39) | <0.001 |
| Republic of Malawi | 3149.9928 (1371.8449,8203.2548) | 15.6363 (7.5997,38.22) | 2950.9749 (1394.103,5718.8099) | 11.1206 (5.2833,21.2699) | -0.06318 | -0.2888 | -1.09 (-1.36 to -0.81) | <0.001 |
| Republic of Maldives | 49.5291 (16.7522,126.8221) | 12.5829 (4.386,30.7785) | 20.0547 (10.8599,35.3507) | 5.9021 (3.0613,11.2131) | -0.59509 | -0.53094 | -2.54 (-2.80 to -2.27) | <0.001 |
| Republic of Mali | 3077.9753 (1328.0393,7212.8062) | 17.2423 (7.6451,38.4581) | 6325.9727 (2875.3344,12695.9384) | 14.1703 (6.7046,27.4749) | 1.055238 | -0.17817 | -0.64 (-0.82 to -0.45) | <0.001 |
| Republic of Malta | 77.2633 (47.581,130.2738) | 28.1726 (17.1776,47.8062) | 44.6824 (28.4822,58.9972) | 20.3025 (12.6056,26.9685) | -0.42169 | -0.27935 | -1.34 (-2.56 to -0.09) | 0.035 |
| Republic of Mauritius | 126.5391 (69.8608,196.2976) | 11.3066 (6.2032,17.6061) | 65.1861 (48.6905,99.6197) | 8.9595 (6.5994,14.2494) | -0.48485 | -0.20759 | -0.72 (-1.52 to 0.08) | 0.076 |
| Republic of Moldova | 1001.0986 (545.0983,1335.9153) | 25.151 (13.6674,33.6829) | 240.5753 (180.049,326.7868) | 13.8607 (10.2264,18.951) | -0.75969 | -0.4489 | -1.88 (-2.64 to -1.11) | <0.001 |
| Republic of Mozambique | 3728.2574 (1623.9505,10768.9666) | 14.9676 (7.0341,40.8295) | 5574.2342 (2510.3196,11845.3122) | 11.3959 (5.3043,23.3008) | 0.495131 | -0.23863 | -0.85 (-1.05 to -0.64) | <0.001 |
| Republic of Namibia | 171.1808 (93.2802,292.7134) | 8.3841 (4.7578,13.9393) | 246.4503 (139.8048,416.856) | 8.9377 (5.0983,15.0066) | 0.439708 | 0.06603 | 0.22 (0.14 to 0.30) | <0.001 |
| Republic of Nauru | 0.7544 (0.3502,1.7769) | 5.0744 (2.4902,11.418) | 1.1807 (0.5748,2.5299) | 8.7082 (4.335,18.4487) | 0.565085 | 0.716104 | 1.75 (1.65 to 1.85) | <0.001 |
| Republic of Nicaragua | 876.1576 (572.8439,1544.3378) | 13.6142 (9.1335,23.5904) | 741.348 (473.5459,1115.4905) | 11.5125 (7.2273,17.577) | -0.15386 | -0.15438 | -0.53 (-0.72 to -0.34) | <0.001 |
| Republic of Niue | 0.1346 (0.0692,0.2909) | 5.5645 (2.8114,12.3129) | 0.2335 (0.0937,0.6439) | 19.693 (7.5026,56.3406) | 0.73477 | 2.539042 | 4.62 (4.31 to 4.93) | <0.001 |
| Republic of Palau | 0.936 (0.4481,2.1478) | 6.0072 (2.8631,13.9254) | 0.8841 (0.4425,1.8499) | 8.2622 (3.7457,19.041) | -0.05545 | 0.375383 | 1.08 (0.91 to 1.25) | <0.001 |
| Republic of Panama | 688.9748 (535.2774,1041.5826) | 24.3918 (18.9721,36.7971) | 987.5986 (609.4296,1338.0612) | 27.1468 (16.5662,36.9525) | 0.433432 | 0.112948 | 0.34 (0.03 to 0.66) | 0.031 |
| Republic of Paraguay | 442.76 (276.9145,944.4161) | 7.3121 (4.6033,15.4506) | 487.4066 (267.6657,833.1379) | 7.5822 (4.0929,13.1234) | 0.100837 | 0.036939 | 0.36 (0.23 to 0.48) | <0.001 |
| Republic of Peru | 3791.5358 (1881.8118,7318.6168) | 12.638 (6.3876,24.2935) | 3420.0571 (1770.6442,7236.2411) | 10.3435 (5.2387,22.0688) | -0.09798 | -0.18156 | -0.71 (-1.11 to -0.30) | <0.001 |
| Republic of Poland | 4607.0745 (1616.5082,6859.9163) | 17.1975 (5.9148,25.7409) | 1651.7208 (592.5423,2470.7753) | 9.431 (3.0942,14.2213) | -0.64148 | -0.45161 | -1.99 (-2.65 to -1.33) | <0.001 |
| Republic of Rwanda | 1905.9496 (946.4669,5475.6781) | 14.4459 (7.7083,39.19) | 1726.2485 (873.6555,3297.2682) | 10.1054 (5.1778,19.0229) | -0.09428 | -0.30047 | -1.10 (-1.51 to -0.68) | <0.001 |
| Republic of San Marino | 0.9534 (0.5842,1.6326) | 6.9664 (4.0673,12.7008) | 0.5433 (0.3302,0.8457) | 3.2547 (1.9239,5.4066) | -0.43014 | -0.5328 | -2.41 (-2.54 to -2.28) | <0.001 |
| Republic of Senegal | 1642.09 (747.4417,3836.4354) | 11.498 (5.6668,25.5343) | 2240.6671 (1051.1183,4549.741) | 10.2993 (4.9285,20.4202) | 0.364521 | -0.10425 | -0.39 (-0.54 to -0.23) | <0.001 |
| Republic of Serbia | 805.804 (459.2818,1358.327) | 11.5093 (6.4555,19.5126) | 242.7117 (158.6533,380.0686) | 5.213 (3.4531,8.3134) | -0.6988 | -0.54706 | -2.53 (-3.26 to -1.78) | <0.001 |
| Republic of Seychelles | 3.9627 (2.104,7.4312) | 4.9997 (2.6456,9.3626) | 5.0645 (2.6411,9.2626) | 6.0771 (3.0356,11.4565) | 0.278043 | 0.215493 | 0.67 (0.48 to 0.86) | <0.001 |
| Republic of Sierra Leone | 1358.3941 (553.5277,3701.6802) | 16.2639 (7.3073,42.2221) | 1610.2894 (803.7679,3164.5597) | 12.1089 (6.3157,23.4109) | 0.185436 | -0.25547 | -0.92 (-1.09 to -0.75) | <0.001 |
| Republic of Singapore | 318.8041 (200.7971,573.7458) | 12.803 (7.9695,23.2019) | 258.2416 (159.923,430.6347) | 7.9048 (4.9131,13.731) | -0.18997 | -0.38258 | -1.55 (-2.13 to -0.97) | <0.001 |
| Republic of Slovenia | 161.5264 (100.218,308.3973) | 13.478 (8.1916,27.1161) | 66.0837 (45.2163,94.6873) | 6.2803 (4.2667,8.862) | -0.59088 | -0.53403 | -2.18 (-2.94 to -1.42) | <0.001 |
| Republic of South Africa | 5101.9085 (3775.5319,7124.7116) | 10.8094 (7.9702,15.0604) | 6044.9735 (4241.7325,8239.5534) | 11.6845 (8.2334,15.8931) | 0.184846 | 0.080957 | 0.27 (0.01 to 0.54) | 0.044 |
| Republic of South Sudan | 1387.1775 (653.6481,3411.6827) | 13.1656 (6.6624,30.749) | 2511.4636 (1176.8879,5960.408) | 15.2839 (7.627,34.7007) | 0.810485 | 0.160897 | 0.50 (0.35 to 0.65) | <0.001 |
| Republic of Sudan | 18864.1074 (4214.0614,57414.9543) | 49.1583 (11.7322,147.9551) | 23543.0175 (12690.4922,45746.4706) | 42.7012 (23.0206,82.8344) | 0.248032 | -0.13135 | -0.46 (-0.52 to -0.40) | <0.001 |
| Republic of Suriname | 47.5226 (23.0891,90.257) | 10.8142 (5.2512,20.5395) | 50.7297 (27.049,96.7596) | 11.086 (5.6491,21.8027) | 0.067486 | 0.025134 | 0.10 (-0.25 to 0.46) | 0.572 |
| Republic of Tajikistan | 821.2366 (394.3597,1825.2047) | 9.0341 (4.5048,19.1744) | 1543.241 (699.1349,3534.5681) | 11.7348 (5.5171,26.3982) | 0.879167 | 0.298945 | 0.84 (0.60 to 1.09) | <0.001 |
| Republic of the Congo | 486.3831 (232.6974,1109.0254) | 12.5093 (6.3935,27.5194) | 713.441 (354.5914,1511.7141) | 11.516 (5.6792,24.5748) | 0.466829 | -0.0794 | -0.26 (-0.35 to -0.16) | <0.001 |
| Republic of the Gambia | 170.111 (79.6642,398.1517) | 9.3358 (4.7119,20.7909) | 310.7029 (149.2641,651.82) | 9.0968 (4.4608,18.4705) | 0.826472 | -0.0256 | -0.09 (-0.33 to 0.14) | 0.448 |
| Republic of the Marshall Islands | 3.9987 (1.8395,9.6831) | 5.9732 (2.8867,14.0496) | 5.8899 (2.4928,14.5403) | 10.2586 (4.2847,25.9208) | 0.472954 | 0.717438 | 1.75 (1.67 to 1.83) | <0.001 |
| Republic of the Niger | 1906.8555 (745.5351,5075.0013) | 11.1137 (4.7483,27.5828) | 4168.3724 (1892.3712,8829.291) | 8.6742 (4.2612,17.5634) | 1.185993 | -0.2195 | -0.84 (-1.08 to -0.60) | <0.001 |
| Republic of the Philippines | 5032.9567 (3728.9074,7372.5196) | 5.5362 (4.1213,7.9667) | 5000.6333 (3450.227,7397.7105) | 4.4057 (3.0489,6.5218) | -0.00642 | -0.2042 | -0.74 (-1.01 to -0.47) | <0.001 |
| Republic of the Union of Myanmar | 4406.7542 (1851.5483,13169.5615) | 8.5732 (3.6923,25.5047) | 3585.4522 (1735.9052,8432.9301) | 6.7759 (3.2197,16.1732) | -0.18637 | -0.20964 | -0.77 (-0.89 to -0.65) | <0.001 |
| Republic of Trinidad and Tobago | 382.1041 (243.8797,531.2964) | 32.2906 (20.3425,44.9673) | 193.224 (127.0815,291.8486) | 24.1991 (15.4997,37.4243) | -0.49432 | -0.25058 | -0.91 (-1.84 to 0.02) | 0.055 |
| Republic of Tunisia | 4488.5282 (2077.4005,8912.4206) | 42.7892 (19.8312,84.8066) | 1626.5034 (870.8523,3284.9556) | 18.7163 (9.5552,39.2685) | -0.63763 | -0.56259 | -2.67 (-2.89 to -2.44) | <0.001 |
| Republic of Turkey | 53818.619 (23671.4203,107485.0543) | 76.5135 (33.7226,152.399) | 12070.7442 (6846.4957,20143.0947) | 22.6154 (12.0789,39.3004) | -0.77571 | -0.70443 | -3.82 (-4.08 to -3.57) | <0.001 |
| Republic of Uganda | 4203.7091 (2013.202,11177.1518) | 11.6123 (6.0659,28.3661) | 8447.155 (4320.7838,17541.9776) | 12.1099 (6.3974,24.3875) | 1.009453 | 0.042851 | 0.14 (-0.05 to 0.33) | 0.142 |
| Republic of Uzbekistan | 1368.1025 (885.4625,2592.1368) | 4.4428 (2.8736,8.256) | 1823.6159 (1179.7684,2888.5895) | 4.9516 (3.1826,7.7368) | 0.332953 | 0.114522 | 0.37 (0.10 to 0.63) | 0.007 |
| Republic of Vanuatu | 11.8072 (5.2113,29.7766) | 4.7668 (2.2421,11.1629) | 28.2524 (13.1175,59.9943) | 7.0459 (3.4112,14.7074) | 1.392811 | 0.478119 | 1.27 (1.08 to 1.46) | <0.001 |
| Republic of Yemen | 11715.4983 (2857.7909,31079.9888) | 39.7953 (10.539,103.7581) | 15032.9529 (7341.9229,28924.0568) | 32.5642 (15.9891,62.4894) | 0.283168 | -0.18171 | -0.63 (-0.80 to -0.45) | <0.001 |
| Republic of Zambia | 2072.4405 (1021.1338,5661.2116) | 13.5716 (7.0729,35.7252) | 3192.4738 (1516.6185,6047.5038) | 11.4943 (5.6502,21.4764) | 0.540442 | -0.15306 | -0.49 (-0.59 to -0.39) | <0.001 |
| Republic of Zimbabwe | 1216.4235 (658.3207,2134.1879) | 7.8176 (4.3388,13.397) | 2057.9273 (1160.3509,3649.4996) | 10.059 (5.753,17.4041) | 0.691785 | 0.286712 | 0.82 (0.71 to 0.94) | <0.001 |
| Romania | 2416.2888 (1753.062,4381.6572) | 15.0858 (10.9544,28.2068) | 807.1228 (591.978,1082.545) | 8.2568 (6.1022,11.0488) | -0.66597 | -0.45268 | -1.62 (-2.52 to -0.71) | <0.001 |
| Russian Federation | 18024.2684 (13745.4985,23167.8962) | 16.1577 (12.0966,20.9019) | 7700.2627 (5348.8499,10705.6499) | 8.494 (5.8339,11.9352) | -0.57278 | -0.47431 | -2.12 (-2.56 to -1.68) | <0.001 |
| Saint Kitts and Nevis | 6.1789 (3.9774,9.524) | 13.7359 (8.8147,21.2) | 3.7494 (2.4785,6.0752) | 11.505 (7.3478,19.4246) | -0.39319 | -0.16241 | -0.60 (-0.85 to -0.36) | <0.001 |
| Saint Lucia | 26.3474 (18.0837,37.393) | 15.5232 (10.6782,21.9671) | 15.7305 (10.8765,24.802) | 16.9614 (11.3631,27.619) | -0.40296 | 0.092648 | 0.25 (-0.09 to 0.59) | 0.153 |
| Saint Vincent and the Grenadines | 30.6716 (18.2336,42.1414) | 24.6662 (14.6166,33.9692) | 11.9135 (7.8996,17.3222) | 16.5644 (10.652,24.9551) | -0.61158 | -0.32846 | -1.40 (-2.12 to -0.67) | <0.001 |
| Slovak Republic | 1374.3991 (733.4496,2180.9489) | 34.8416 (18.3008,55.6793) | 529.0592 (340.1364,745.6039) | 18.4101 (11.6614,26.3068) | -0.61506 | -0.47161 | -2.09 (-2.41 to -1.76) | <0.001 |
| Socialist Republic of Viet Nam | 2799.7902 (1486.203,5225.054) | 3.1396 (1.6889,5.7459) | 2425.0474 (1423.6574,3907.1919) | 2.8331 (1.6361,4.6614) | -0.13385 | -0.09762 | -0.33 (-0.46 to -0.21) | <0.001 |
| Solomon Islands | 29.0646 (13.1661,80.9955) | 5.1829 (2.5115,13.1871) | 67.683 (30.1466,163.2627) | 7.3979 (3.4692,17.2413) | 1.328709 | 0.427367 | 1.17 (1.05 to 1.30) | <0.001 |
| State of Eritrea | 640.8636 (315.2234,1519.1636) | 10.636 (5.5172,23.7389) | 880.3055 (430.9869,1884.3566) | 9.9538 (5.015,20.7918) | 0.373624 | -0.06414 | -0.16 (-0.36 to 0.04) | 0.113 |
| State of Israel | 1358.8306 (921.31,2219.1522) | 26.7689 (18.1046,43.7725) | 901.248 (608.3063,1234.2104) | 10.0547 (6.781,13.793) | -0.33675 | -0.62439 | -3.12 (-3.89 to -2.34) | <0.001 |
| State of Kuwait | 695.132 (431.0904,1482.3546) | 40.7368 (25.0023,88.4637) | 1207.1401 (775.3638,1624.7253) | 46.2361 (29.0494,63.1966) | 0.736562 | 0.134996 | 1.17 (0.36 to 1.99) | 0.005 |
| State of Libya | 2839.445 (1361.3578,5780.129) | 45.8014 (22.0657,92.8378) | 1793.5112 (1004.0283,3326.9897) | 42.2279 (22.5192,82.3572) | -0.36836 | -0.07802 | -0.49 (-1.36 to 0.40) | 0.282 |
| State of Qatar | 110.9505 (59.0659,209.897) | 20.99 (11.2477,38.8264) | 259.5855 (162.6169,397.727) | 12.0678 (7.6402,19.1646) | 1.339651 | -0.42507 | -1.71 (-1.84 to -1.58) | <0.001 |
| Sultanate of Oman | 1099.9136 (428.7985,2654.4533) | 32.9106 (13.3499,78.1262) | 892.19 (441.1625,1697.9357) | 22.2296 (10.6076,43.5398) | -0.18885 | -0.32455 | -1.30 (-1.54 to -1.06) | <0.001 |
| Swiss Confederation | 1004.7695 (717.5116,1844.6818) | 23.9049 (16.9795,44.4831) | 658.6141 (472.8157,850.9974) | 14.3058 (10.2427,18.5408) | -0.34451 | -0.40155 | -1.78 (-2.62 to -0.93) | <0.001 |
| Syrian Arab Republic | 8250.8675 (3364.7408,17463.4622) | 38.4 (16.0173,80.3742) | 1710.6724 (772.7331,3755.2808) | 16.0978 (6.6398,37.1932) | -0.79267 | -0.58079 | -2.78 (-3.23 to -2.33) | <0.001 |
| Taiwan (Province of China) | 1373.0916 (1043.9579,1771.9087) | 7.9082 (6.0275,10.0737) | 770.117 (590.8877,1010.7388) | 7.2837 (5.4643,9.7469) | -0.43914 | -0.07897 | -0.24 (-1.09 to 0.60) | 0.57 |
| Togolese Republic | 739.0156 (342.0121,1800.6491) | 10.9391 (5.5167,25.0467) | 1076.4142 (519.0064,2106.749) | 9.6914 (4.695,18.6067) | 0.456551 | -0.11406 | -0.39 (-0.49 to -0.28) | <0.001 |
| Tokelau | 0.0821 (0.0416,0.1794) | 4.4571 (2.2479,9.8115) | 0.153 (0.0562,0.3943) | 15.7929 (5.1877,43.1682) | 0.863581 | 2.543313 | 4.86 (4.32 to 5.40) | <0.001 |
| Turkmenistan | 661.9733 (400.8647,971.4547) | 11.6367 (7.1728,16.8896) | 855.1388 (600.1081,1230.7373) | 16.0572 (11.2746,23.0986) | 0.291803 | 0.379876 | 1.08 (-0.00 to 2.17) | 0.051 |
| Tuvalu | 0.9802 (0.3784,2.9566) | 6.8184 (2.916,19.2687) | 0.9267 (0.427,2.0186) | 7.2022 (3.311,15.7367) | -0.05458 | 0.056289 | 0.19 (0.11 to 0.27) | <0.001 |
| Ukraine | 5091.1634 (3660.4641,7262.3798) | 14.2033 (9.9033,20.4382) | 1441.3797 (1018.5281,2247.9112) | 6.7879 (4.7253,10.386) | -0.71689 | -0.52209 | -2.38 (-2.73 to -2.03) | <0.001 |
| Union of the Comoros | 109.8344 (49.1071,275.3696) | 13.159 (6.2682,31.3378) | 88.2703 (40.6532,175.2067) | 10.8025 (4.9651,21.4306) | -0.19633 | -0.17908 | -0.65 (-0.74 to -0.56) | <0.001 |
| United Arab Emirates | 1036.9766 (489.2843,2056.9215) | 45.2717 (21.6218,89.3416) | 827.1606 (461.705,1443.3749) | 18.0053 (9.1952,34.4791) | -0.20233 | -0.60228 | -2.80 (-3.49 to -2.10) | <0.001 |
| United Kingdom of Great Britain and Northern Ireland | 4877.3752 (3569.8397,7375.9696) | 12.25 (8.9666,18.6688) | 3322.51 (2545.2234,4615.735) | 8.733 (6.6301,12.2049) | -0.31879 | -0.2871 | -1.14 (-1.60 to -0.68) | <0.001 |
| United Mexican States | 32561.1689 (23427.2256,55473.467) | 27.994 (20.2891,47.272) | 33186.3874 (24212.6628,42022.6906) | 31.9505 (23.1294,41.6431) | 0.019201 | 0.141334 | 0.44 (0.15 to 0.73) | 0.003 |
| United Republic of Tanzania | 6981.3497 (3471.5303,19945.3102) | 14.0854 (7.4419,38.1941) | 10676.5905 (5111.0291,21454.0473) | 12.5078 (6.0815,24.3937) | 0.529302 | -0.112 | -0.38 (-0.47 to -0.29) | <0.001 |
| United States of America | 41572.3838 (30711.0984,55441.1761) | 20.5147 (15.1308,27.4324) | 25472.4139 (18460.8855,31910.2096) | 13.5103 (9.6984,17.2385) | -0.38728 | -0.34143 | -1.29 (-1.69 to -0.89) | <0.001 |
| United States Virgin Islands | 8.4094 (3.9145,17.6494) | 7.5925 (3.5445,15.9087) | 2.1263 (1.1159,3.897) | 4.5553 (2.1705,9.4739) | -0.74715 | -0.40003 | -1.57 (-1.70 to -1.44) | <0.001 |
