# Supplementary material for: Burden of urogenital congenital anomalies: findings from the global burden of disease study 2021
Source: Front Pediatr. 2025 Sep 25;13:1584280. doi: 10.3389/fped.2025.1584280 (PMC12507638; doi:10.3389/fped.2025.1584280)
Supplement: Supplementary file 2 [file Supplementaryfile2.pdf]

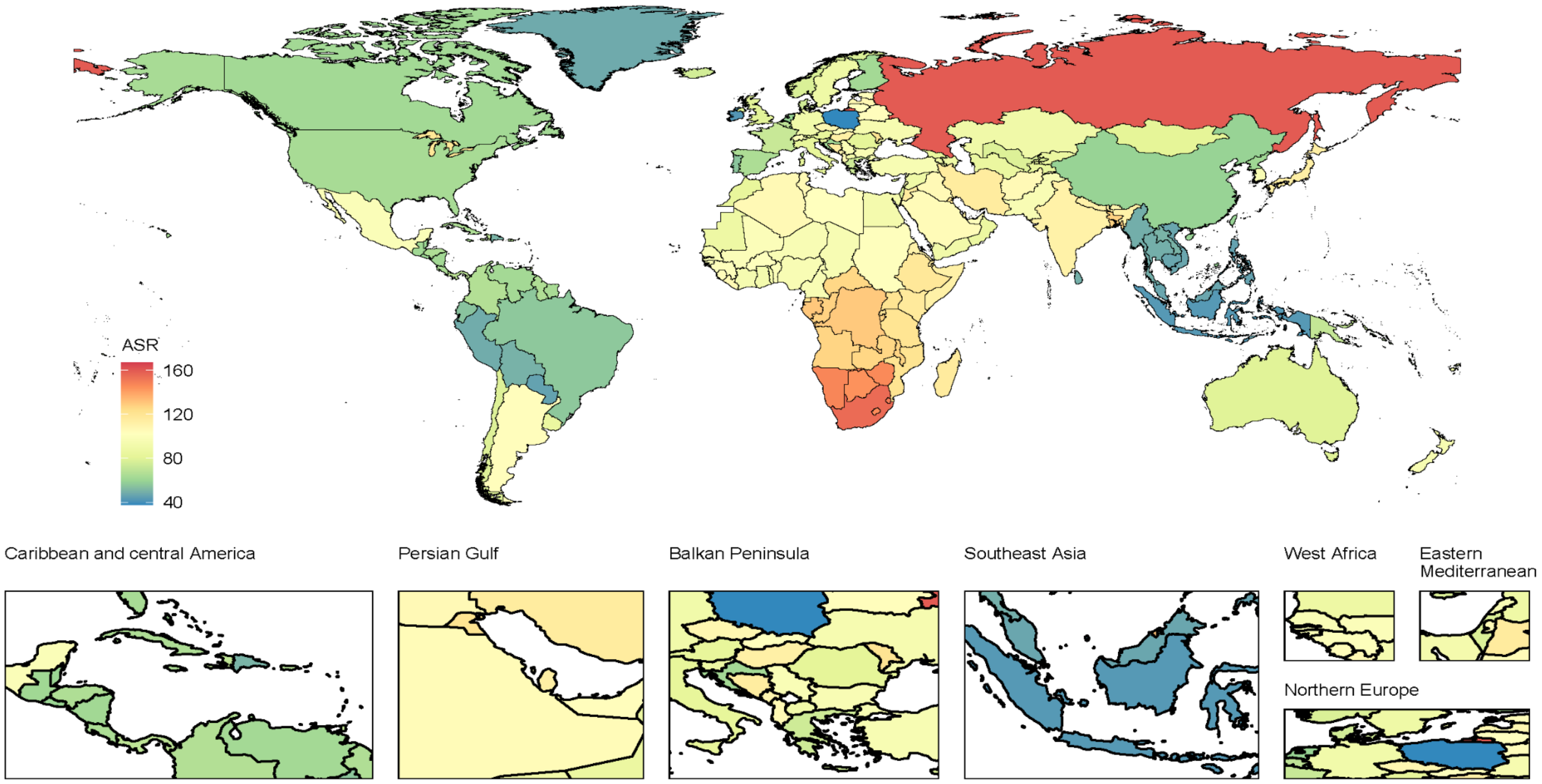

Figure S1 Age-standardized rates per 100,000 cases of prevalence for in 1990 across 204 countries and territories

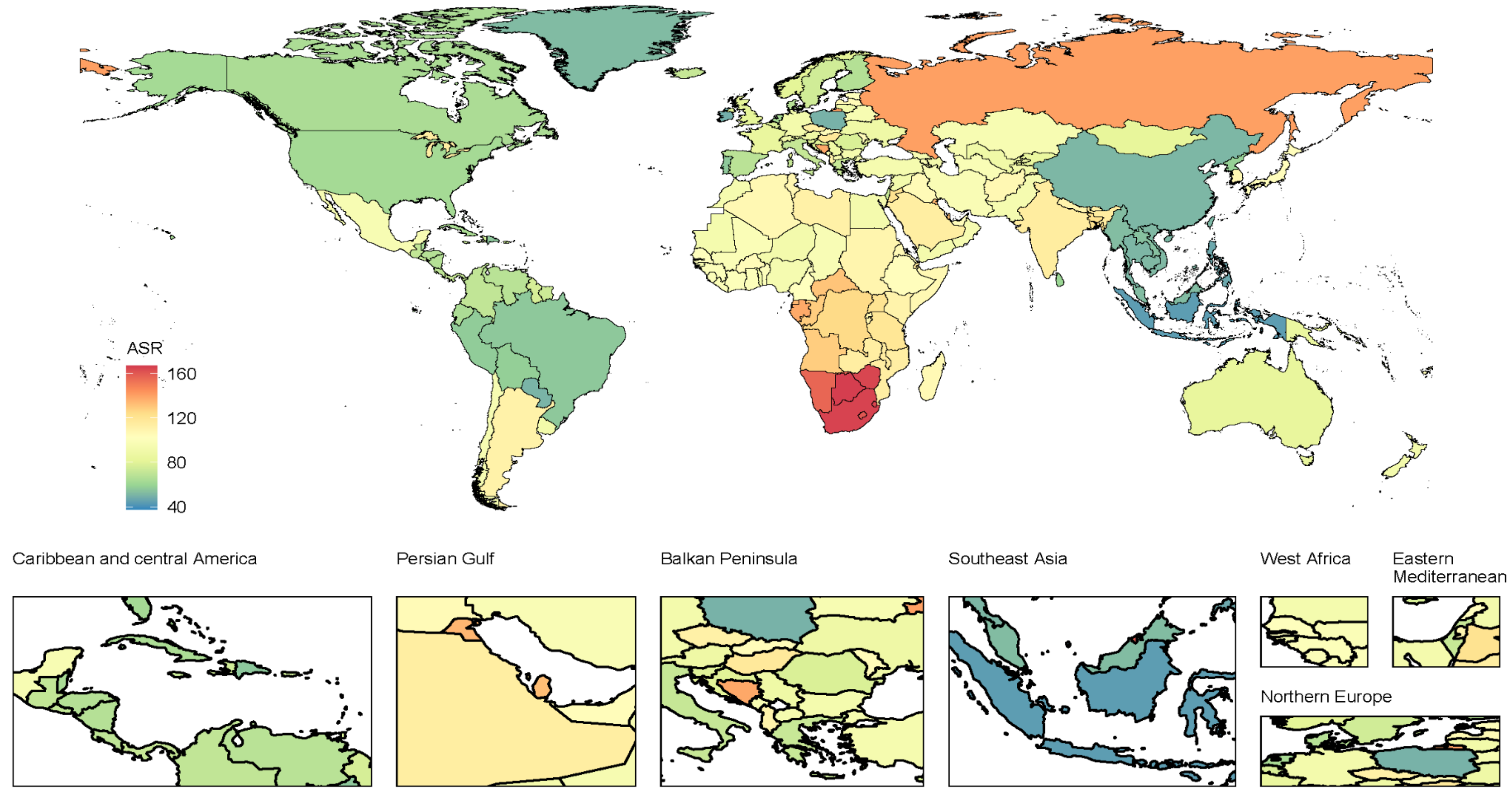

Figure S2 Age-standardized rates per 100,000 cases of prevalence for in 2021 across 204 countries and territories

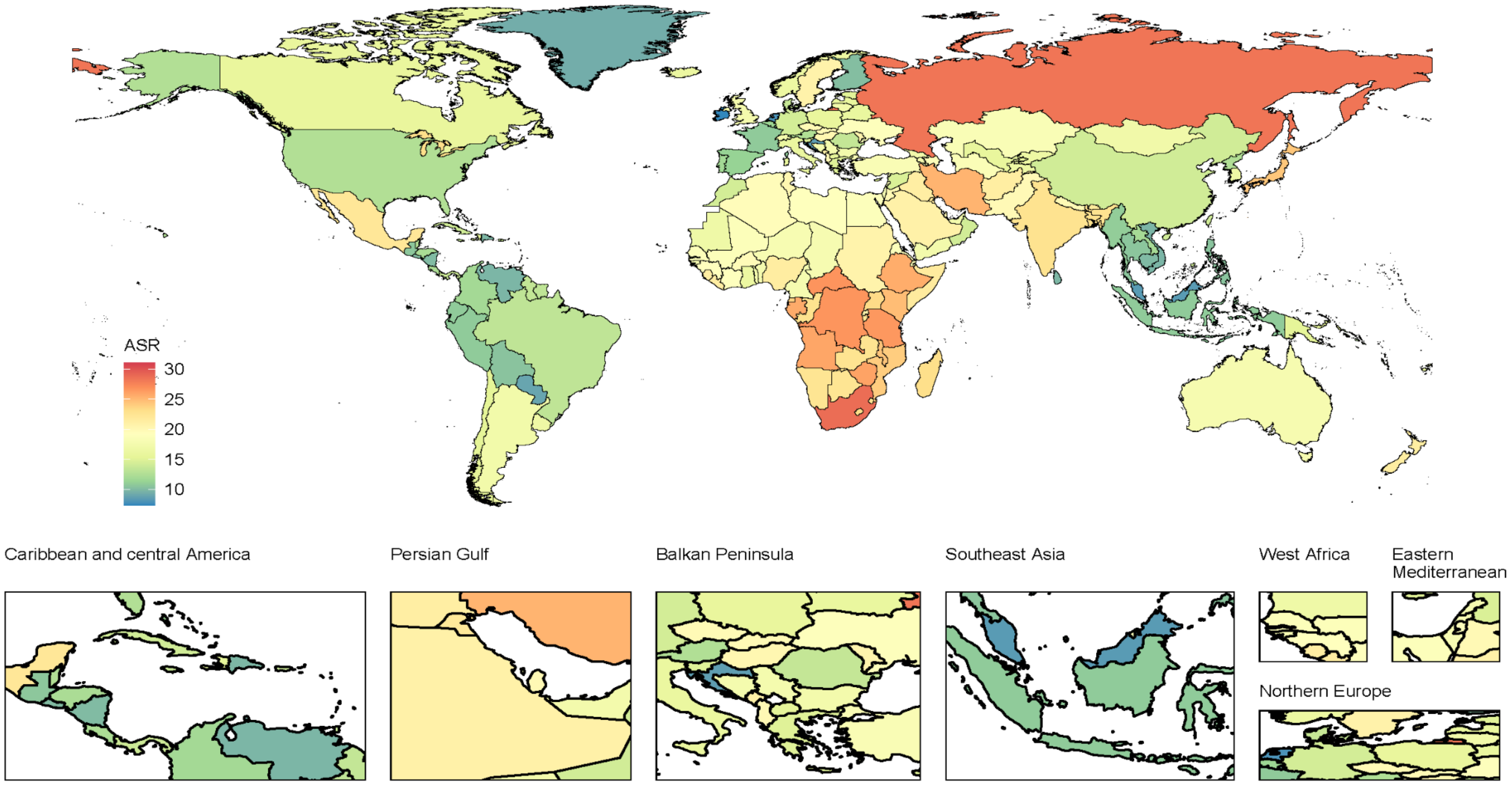

Figure S3 Age-standardized rates per 100,000 cases of incidence for in 1990 across 204 countries and territories

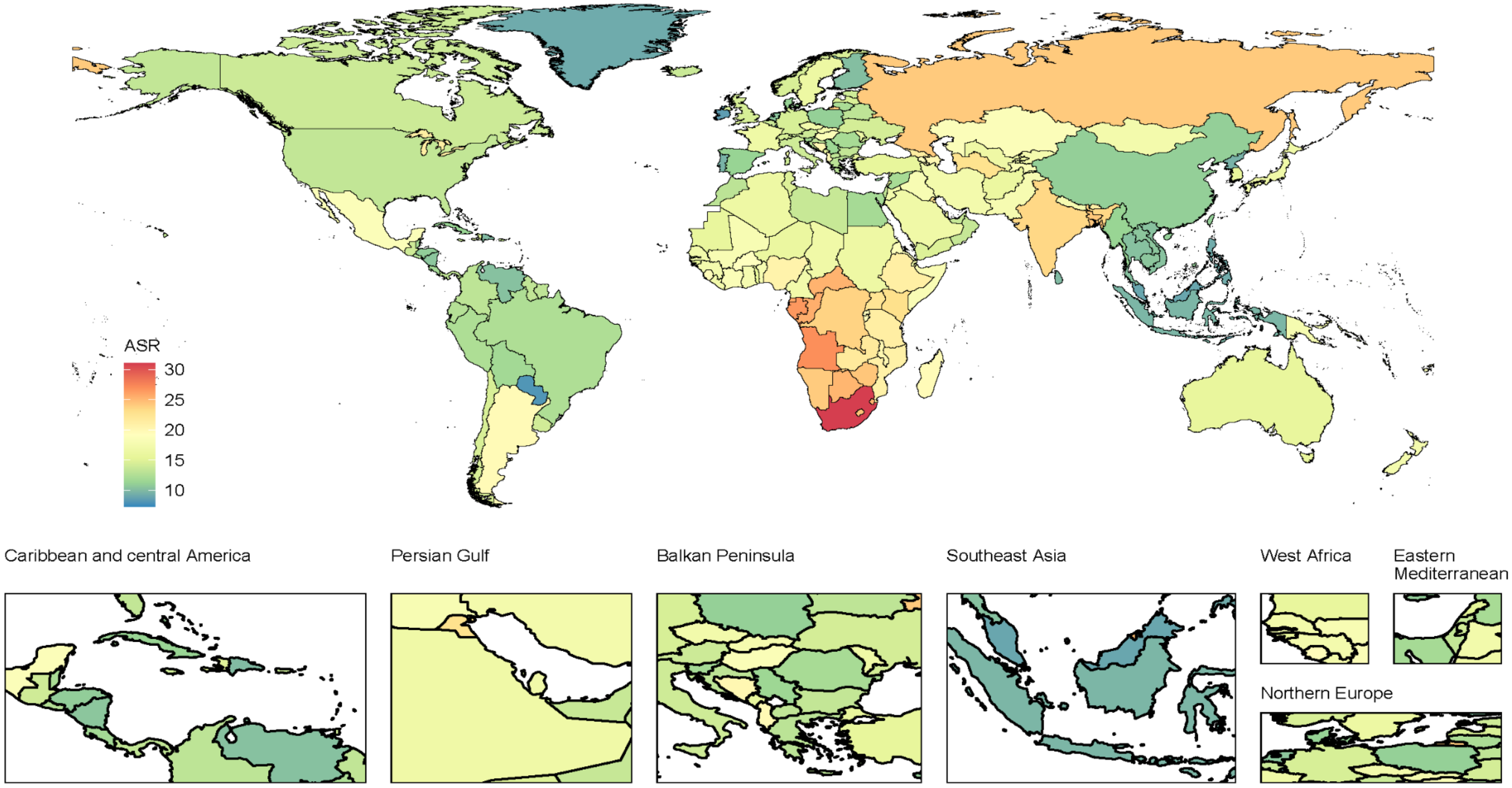

Figure S4 Age-standardized rates per 100,000 cases of incidence for in 2021 across 204 countries and territories

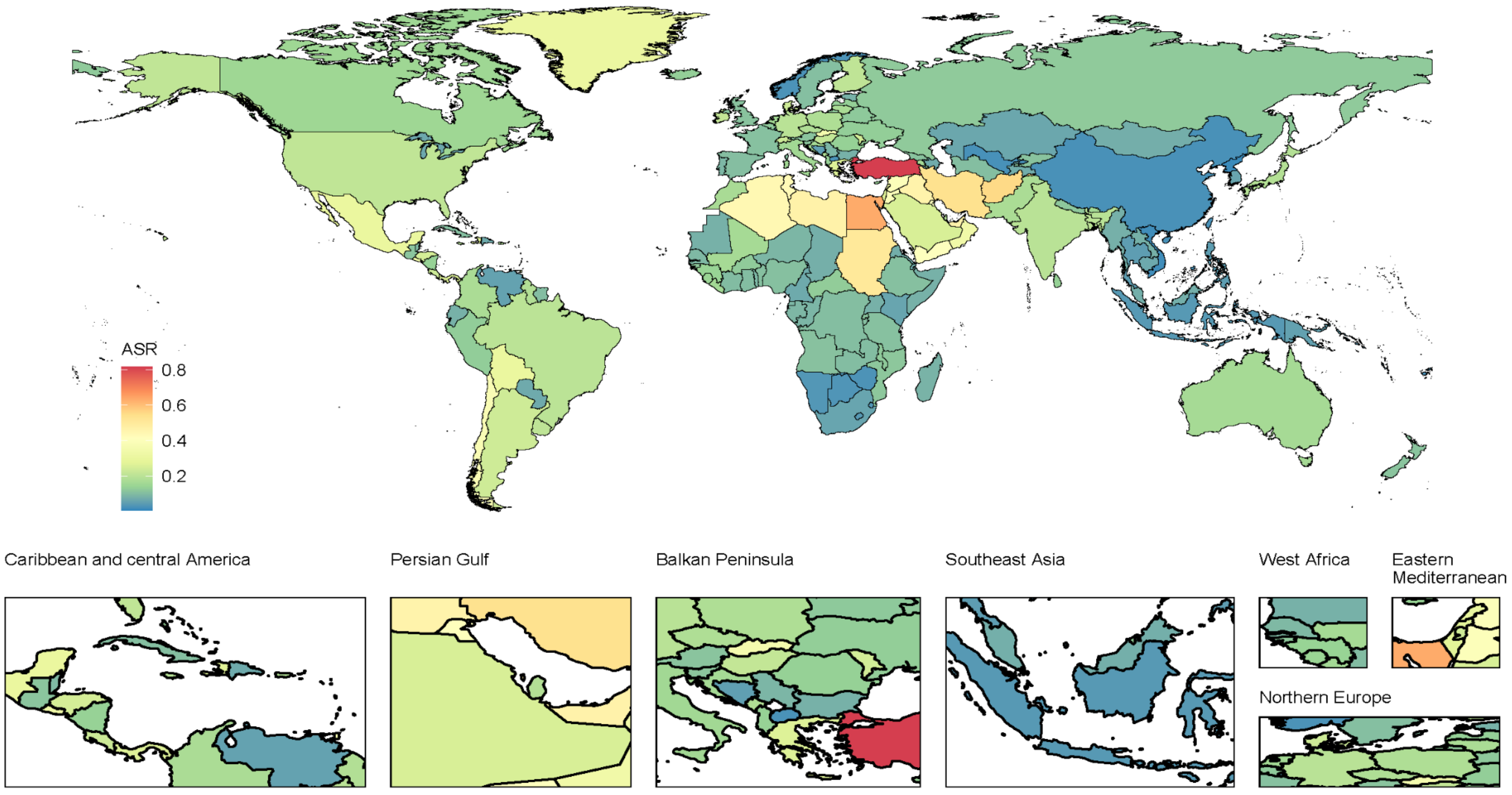

Figure S5 Age-standardized rates per 100,000 cases of deaths for in 1990 across 204 countries and territories

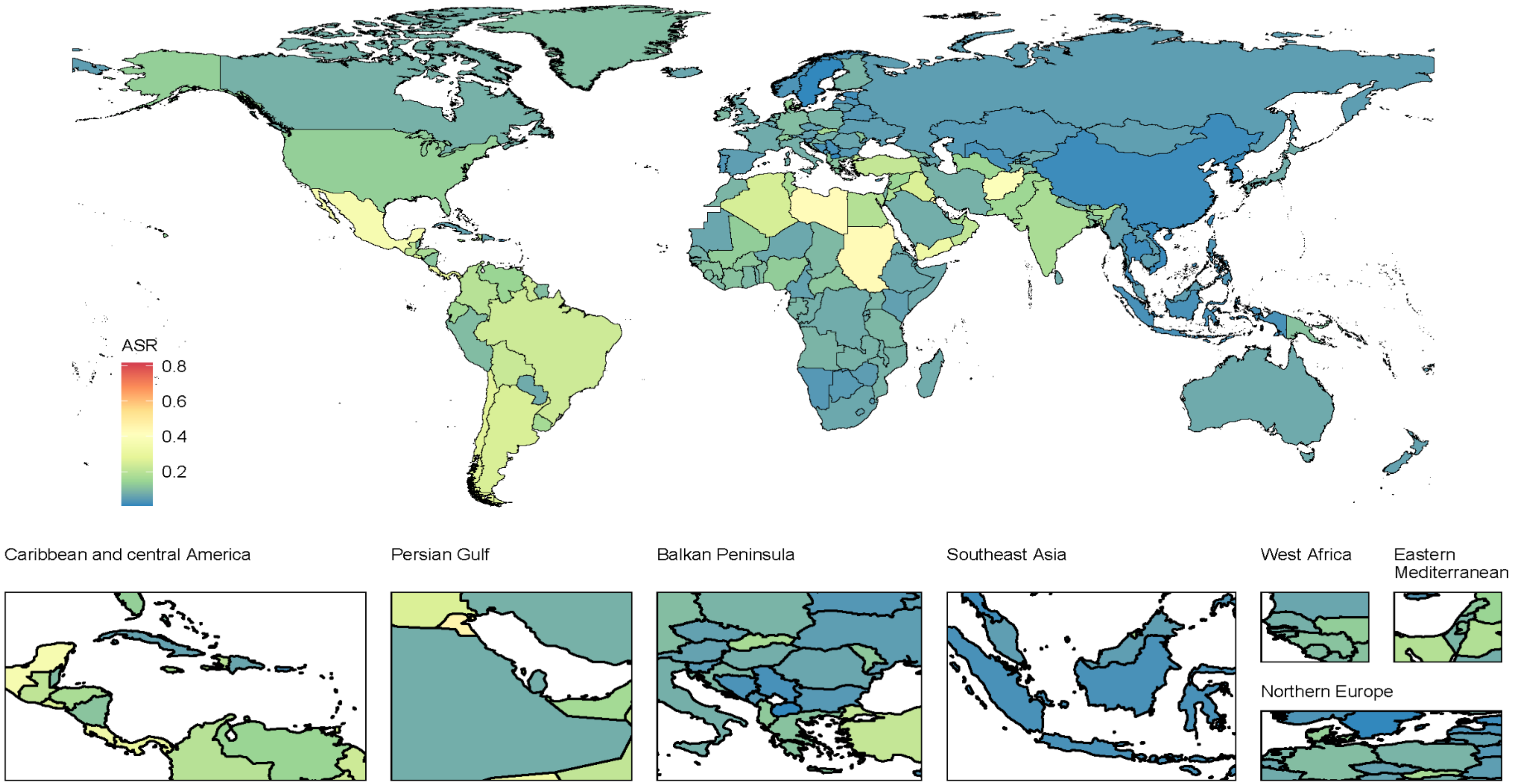

Figure S6 Age-standardized rates per 100,000 cases of deaths for in 2021 across 204 countries and territories

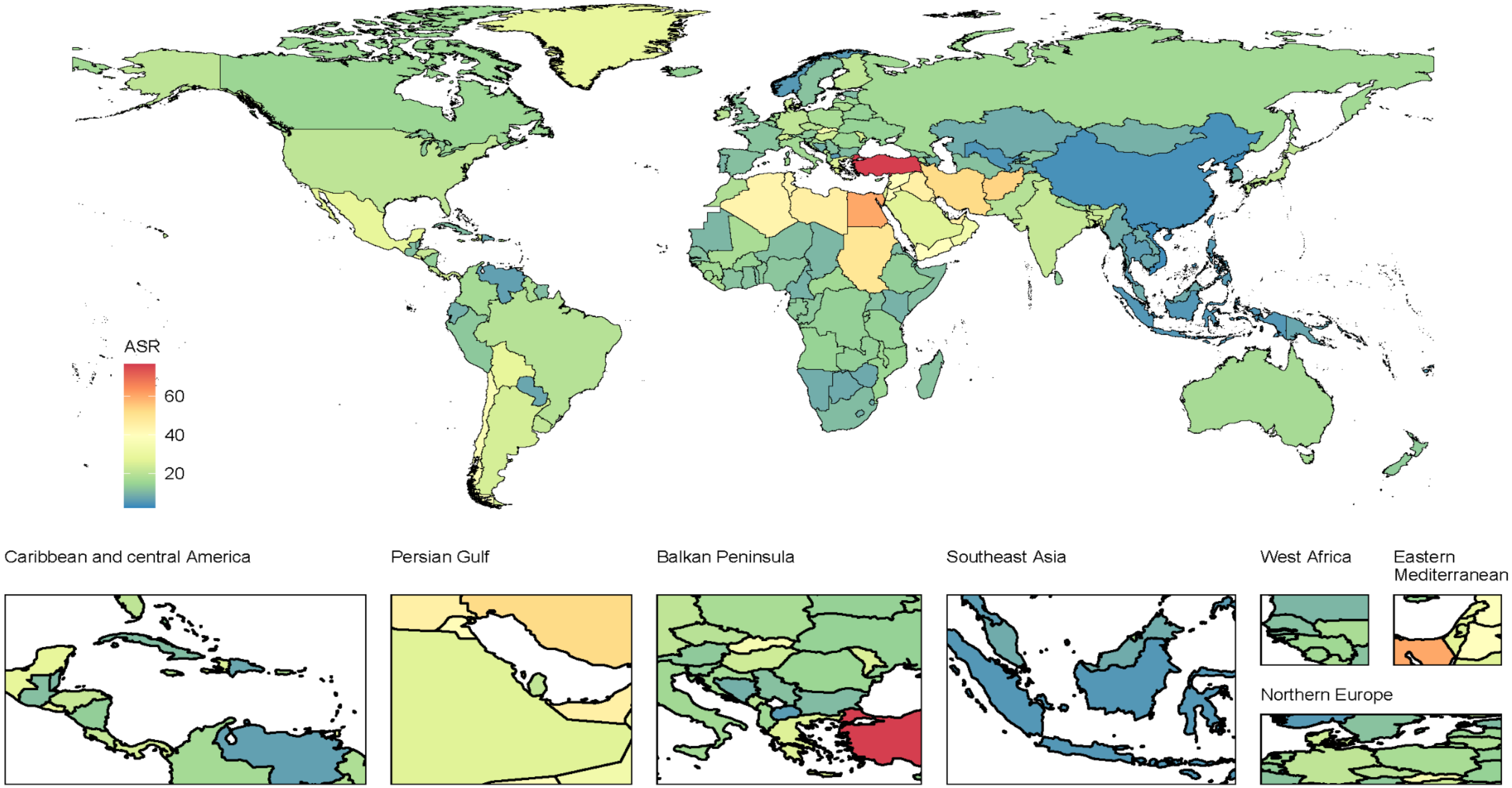

Figure S7 Age-standardized rates per 100,000 cases of DALYs for in 1990 across 204 countries and territories

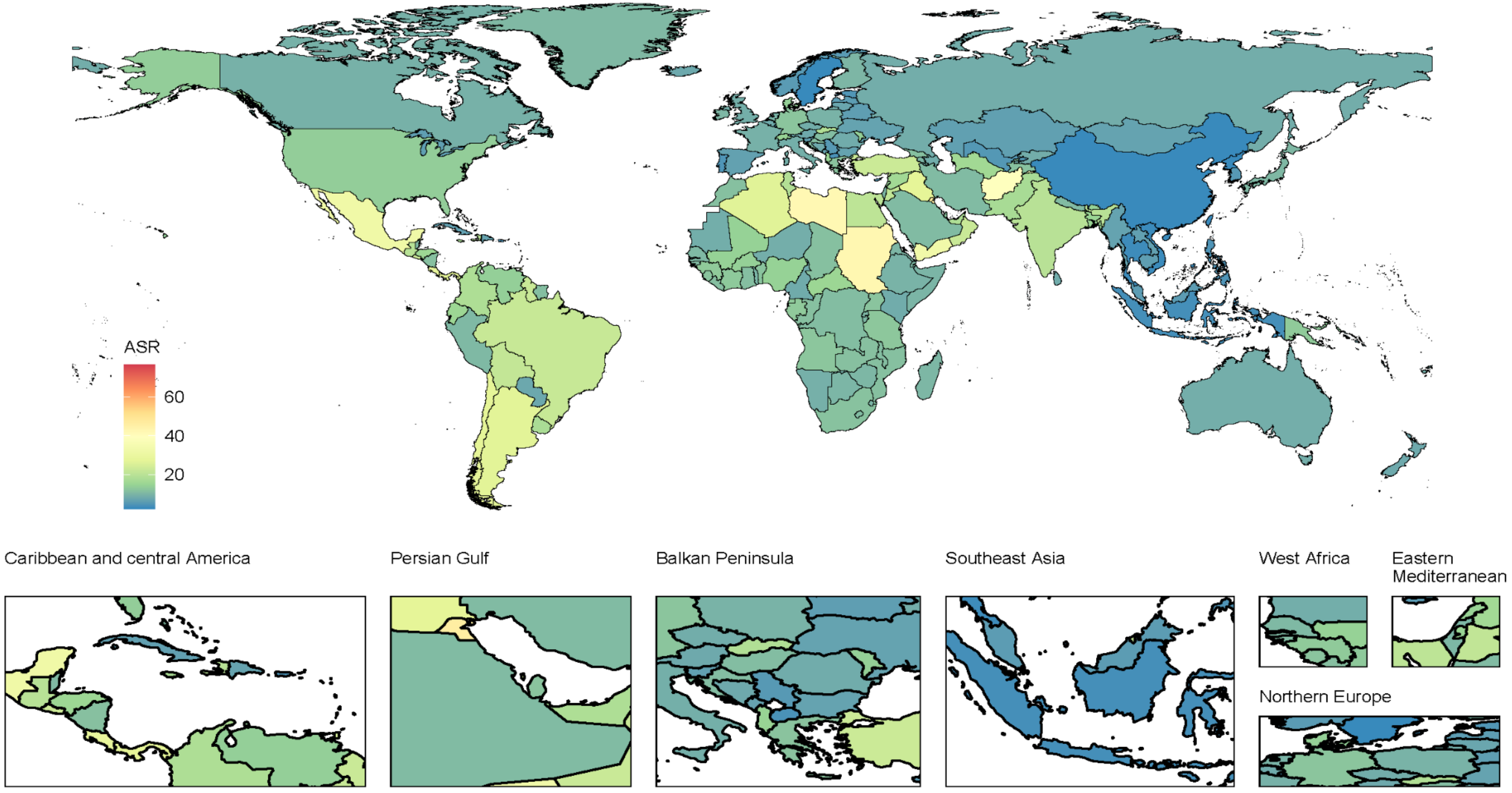

Figure S8 Age-standardized rates per 100,000 cases of DALYs for in 2021 across 204 countries and territories
